# Anti-*Vibrio* Indole-Diterpenoids and C-25 Epimeric Steroids From the Marine-Derived Fungus *Penicillium janthinellum*

**DOI:** 10.3389/fchem.2019.00080

**Published:** 2019-02-15

**Authors:** Xing-Chen Guo, Lan-Lan Xu, Rui-Yun Yang, Meng-Yue Yang, Lian-Dong Hu, Hua-Jie Zhu, Fei Cao

**Affiliations:** ^1^Key Laboratory of Pharmaceutical Quality Control of Hebei Province, Key Laboratory of Medicinal Chemistry and Molecular Diagnostics of Education Ministry of China, College of Pharmaceutical Sciences, Hebei University, Baoding, China; ^2^State Key Laboratory for Chemistry and Molecular Engineering of Medicinal Resources, College of Chemistry and Pharmaceutical Sciences, Guangxi Normal University, Guilin, China

**Keywords:** *Penicillium janthinellum*, indole-diterpenoid, steroid, absolute configuration, anti-*Vibrio* activity

## Abstract

A systematic chemical exploration of the marine-derived fungus *Penicillium janthinellum* led to the isolation of four indole-diterpenoid derivatives (**1**–**4**), including new penijanthines C and D (**1** and **2**), and a pair of new steroidal epimers, penijanthoids A and B (**5** and **6**). The calculated ECD spectra and Snatzke's method for the new compound **1** were carried out to determine its absolute configuration. The absolute configuration of **3** was established by X-ray diffraction and calculated ECD methods for the first time. DP4plus approach was used to elucidate the absolute configurations of the C-25 epimeric steroids **5** and **6**. 25-Epimeric **5** and **6** represent the first examples of steroids forming a five-membered lactone between C-23 and C-27 from marine fungi. Compounds **1**, **2**, **5**, and **6** displayed significant anti-*Vibrio* activity (Minimum inhibitory concentration, MIC values ranging from 3.1 to 50.0 μM) against three pathogenic *Vibrio* spp.

## Introduction

**Figure 1:**
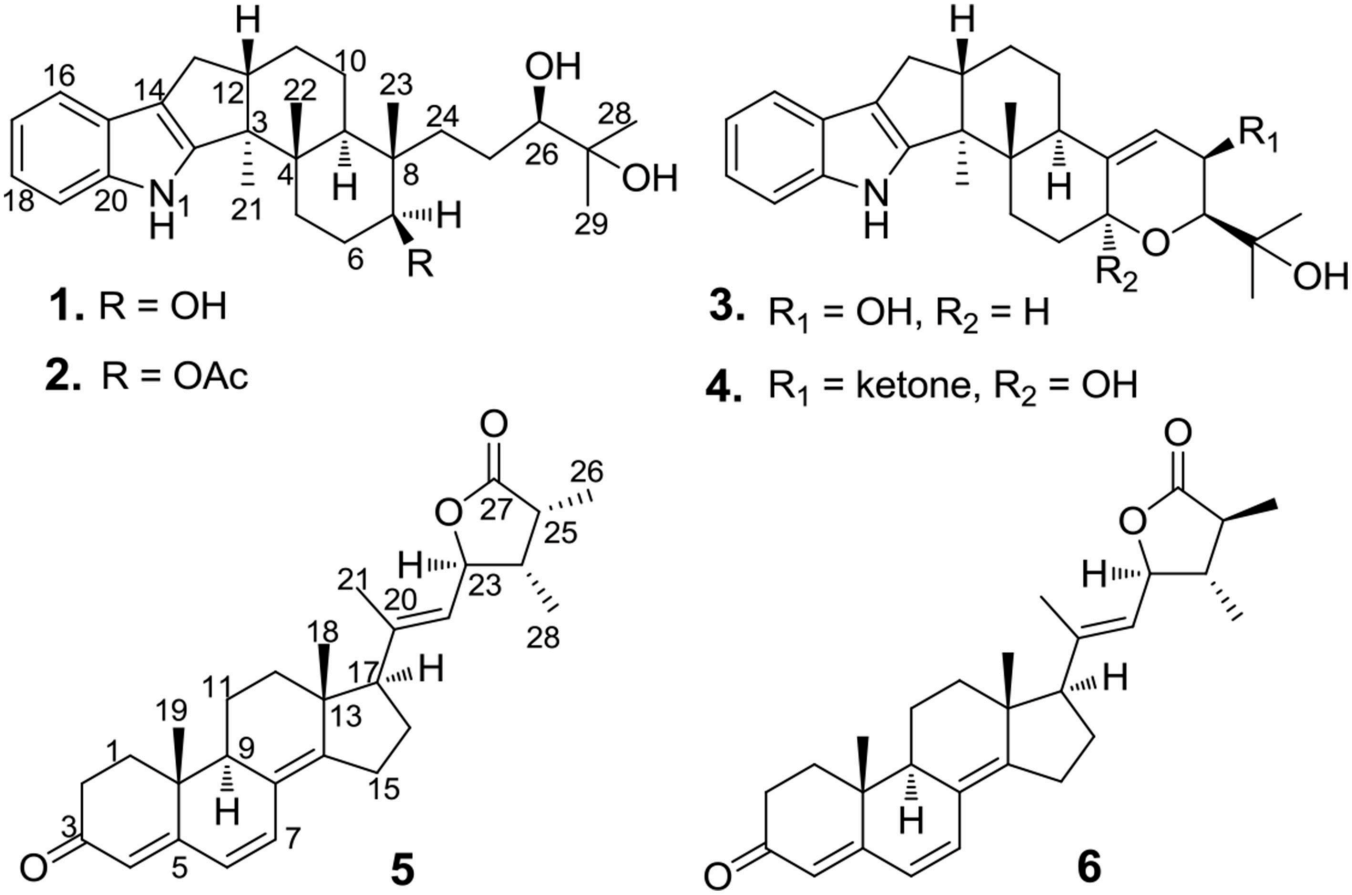
Chemical structures of **1**–**6**.

*Vibrio* spp., such as *Vibrio anguillarum, Vibrio parahemolyticus*, and *Vibrio alginolyticus*, is a class of Gram-negative halophilic bacteria that occurs usually in marine and coastal environments throughout the world, which could lead vibriosis in crustaceans and cause serious damage to mariculture production (Vezzulli et al., 2015; Moreno et al., 2017). However, there was no effective vaccine to prevent vibriosis due to lacking adaptive immunity in crustacean species (Buchmann, 2014). In the past few decades, searching for marine-derived bioactive substances as anti-*Vibrio* agents has drawn the attention of chemists and pharmacologists (Meng et al., 2015; Wang et al., 2015). In our continuing efforts to explore anti-*Vibrio* natural products from marine-derived fungi (Xu et al., 2017; Yang et al., 2018), the Bohai Sea fungus *Penicillium janthinellum* was selected for further chemical exploration due to the anti-*Vibrio* activity of its EtOAc extract. As a result, two new indole-diterpenoids, penijanthines C and D (**1** and **2**), and two known analogs, PC-M6 (**3**) (Yamaguchi et al., 1993), 7-hydroxy-13-dehydroxypaxilline (**4**) (Mantle and Weedon, 1994), along with two new steroids, penijanthoids A and B (**5** and **6**), were obtained (Figure 1). Compounds **1**–**6** displayed anti-*Vibrio* activity against three pathogenic *V. anguillarum, V. parahemolyticus*, and *V. alginolyticus*.

## Materials and Methods

### General Experimental Procedures

Optical rotations (OR) values of the new compounds were determined using a JASCO-1020 polarimeter. Electronic circular dichroism (ECD) experiments, including Mo_2_(AcO)_4_ ICD experiments, were carried out on a JASCO J-815 circular dichroism spectrometer. Ultraviolet–visible (UV) data were provided in MeOH by a Perkin-Elmer model 241 spectrophotometer. Infrared radiation (IR) data of the new compounds (using KBr pellets) were measured on a Nicolet NEXUS 470 spectrophotometer. 1D NMR (^1^H NMR and ^13^C NMR) and 2D NMR (HSQC, ^1^H-^1^H COZY, HMBC and NOESY) data were recorded on a Bruker AV-600 spectrometer. HR-ESI-MS spectra were performed with a Thermo Scientific LTQ Orbitrap XL spectrometer. Semi-preparation HPLC, which had the Shimadzu LC-20AT system with a SPD-M20A detector and a Waters RP-18 column, was used for chemical separation. Further chromatographic separation was taken on 200–300 mesh silica gel and 18–110 μm Sephadex LH-20 columns.

### Isolation of the Fungal Material

The strain in our research, which was derived from the marine sediment collected from the Bohai Sea in June 2016, was deposited at Hebei University, China. According to its 16S rRNA amplification and sequencing of the ITS region, the strain was identified as *Penicillium janthinellum* (Gene Bank KY979507). The fungus *Penicillium janthinellum* was cultivated using solid medium in forty Erlenmeyer flasks (80 g raw rice, 60 mL H_2_O, 2.0 g sea salt in each Erlenmeyer flask) at 28°C for 4 weeks. Mixed solvent of CH_2_Cl_2_-MeOH (v/v = 1:1) was used to extract fermented rice, and the solution was evaporated to give the crude extract, which was dissolved and extracted with EtOAc for five times to provide the EtOAc extract (12.0 g). The EtOAc extract, which was eluted with EtOAc-petroleum ether (PE) on the silica gel column chromatography (CC), was separated into different fractions ranged from Fr.1 to Fr.8. Fr.2 (1.46 g), which was eluted with 40% EtOAc in PE, was applied on a Sephadex LH-20 and waters RP-18 (XBridge OBD, 5 μm, 10 × 250 mm, 70%-MeOH in water) columns to produce **5** (10.5 mg) and **6** (7.6 mg). Fr.4 (4.34 g, 60% EtOAc in PE) was separated by repeatedly silica gel CC and HPLC to provide **1** (20.5 mg), **2** (5.8 mg), **3** (16.4 mg), and **4** (8.2 mg).

Penijanthine C (**1**)

Yellow powder; [α]${}_{\text{D}}^{20}$ −80.0 (*c* 0.20, CH_2_Cl_2_); UV (MeOH) λ_max_ (log ε) 230 (2.60), 280 (0.85) nm; CD (MeOH) λ_max_ (Δε) 230 (13.1), 291 (−1.2) nm; IR (KBr) *v*_max_ 3,473, 2,945, 1,628, 1,302, 1,240, 1,054, 931, 820 cm^−1^; ^1^H and ^13^C NMR data, see Table 1; HRESIMS *m/z* 440.3163 [M + H]^+^ (calcd. for C_28_H_42_O_3_N, 440.3159).

**Table 1:** ^1^H (600 MHz) and ^13^C (150 MHz) NMR Data for **1** and **2** (DMSO-*d*_6_).

**Position**	**1**		**2**	

	***δ***_**H**_**, mult. (*****J*** **in Hz)**	***δ***_**C**_**, type**	***δ***_**H**_**, mult. (*****J*** **in Hz)**	***δ***_**C**_**, type**
1	10.56, s		10.55, s	
2		151.3, C		151.0, C
3		52.6, C		52.5, C
4		38.6, C		38.5, C
5	1.86, m	32.1, CH_2_	1.89, m	31.6, CH_2_
	1.70, m		1.69, m	
6	1.70, m	27.2, CH_2_	1.69, m	23.5, CH_2_
	1.64, t (12.6)			
7	3.34, m	71.4, CH	4.56, m	75.4, CH
8		48.6, C		39.9, C
9	1.64, t (12.6)	40.8, CH	1.66, m	40.1, CH
10	1.53, m	22.3, CH_2_	1.50, m	22.0, CH_2_
	1.35, dt (12.6, 12.0)		1.33, m	
11	1.69, m	25.0, CH_2_	1.69, m	24.9, CH_2_
	1.53, m		1.50, m	
12	2.63, m	48.5, CH	2.60, m	48.5, CH
13	2.56, dd (12.6, 6.0)	27.1, CH_2_	2.51, dd (12.6, 6.6)	27.0, CH_2_
	2.24, dd (12.6, 11.2)		2.20, dd (12.6, 11.2)	
14		115.8, C		115.9, C
15		124.4, C		124.4, C
16	7.27, d (7.8)	117.5, CH	7.21, d (7.8)	117.6, CH
17	6.89, dd (7.8, 7.2)	118.3, CH	6.84, dd (7.8, 7.2)	118.4, CH
18	6.94, dd (7.8, 7.2)	119.2, CH	6.88, dd (7.8, 7.2)	119.3, CH
19	7.27, d (7.8)	111.8, CH	7.21, d (7.8)	111.8, CH
20		140.2, C		140.2, C
21	0.94, s	14.7, CH_3_	0.91, s	14.6, CH_3_
22	1.02, s	18.6, CH_3_	1.00, s	18.6, CH_3_
23	0.75, s	17.1, CH_3_	0.83, s	17.5, CH_3_
24	1.85, m	34.8, CH_2_	1.50, m	35.2, CH_2_
	1.06, m		1.08, m	
25	1.44, t (11.8)	23.6, CH_2_	1.67, m	23.8, CH_2_
	1.15, m		1.36, m	
26	3.00, t (7.2)	78.6, CH	2.90, m	78.0, CH
27		71.7, C		71.6, C
28	1.03, s	24.6, CH_3_	0.94, s	24.6, CH_3_
29	1.08, s	26.4, CH_3_	0.99, s	26.4, CH_3_
-OAc			1.94, s	21.0, CH_3_
				169.9, C
7-OH	4.26, s			
26-OH	4.21, s		4.12, s	
27-OH	4.10, s		4.04, s	

Penijanthine D (**2**)

Yellow powder; [α]${}_{\text{D}}^{20}$ −27.0 (*c* 0.20, CH_2_Cl_2_); UV (MeOH) λ_max_ (log ε) 240 (3.50), 280 (3.20) nm; CD (MeOH) λ_max_ (Δε) 230 (11.4), 291 (−1.7) nm; IR (KBr) *v*_max_ 3,435, 2,943, 1,641, 1,629, 1,312, 1,233, 1,054, 930, 819 cm^−1^; ^1^H and ^13^C NMR data, see Table 1; HRESIMS *m/z* 504.3072 [M + Na]^+^(calcd. for C_30_H_43_O_4_NNa, 504.3084).

Penijanthoid A (**5**)

Colorless powder; [α]${}_{\text{D}}^{25}$ +424 (*c* 3.0, CH_2_Cl_2_); UV (MeOH) λ_max_ (log ε) 350 (1.50) nm; CD (MeOH) λ_max_ (Δε) 223 (7.1), 247 (3.4), 363 (24.3) nm; IR (KBr) *v*_max_ 2,932, 2,249, 1,725, 1,650, 1,600, 1,468, 1,395, 1,176 cm^−1^; ^1^H and ^13^C NMR data, see Table 2; HRESIMS *m/z* 421.2732 [M + H]^+^ (calcd. for C_28_H_36_O_3_, 421.2737) (Figure S24).

**Table 2:** ^1^H (600 MHz) and ^13^C (150 MHz) NMR Data for **5** and **6** (CDCl_3_).

**Position**	**5**		**6**	
	***δ***_**H**_**, mult. (*****J*** **in Hz)**	***δ***_**C**_**, type**	***δ***_**H**_**, mult. (*****J*** **in Hz)**	***δ***_**C**_**, type**
1	2.03, m 1.81, m	34.1, CH_2_	2.03, m 1.81, m	34.1, CH_2_
2	2.54, m	34.1, CH_2_	2.55, m	34.1, CH_2_
3		199.4, C		199.4, C
4	5.75, s	123.4, CH	5.75, s	123.4, CH
5		164.0, C		164.0, C
6	6.06, d (9.5)	125.0, CH	6.06, d (9.5)	125.0, CH
7	6.60, d (9.5)	133.5, CH	6.61, d (9.5)	133.5, CH
8		125.4, C		125.4, C
9	2.18, m	44.6, CH	2.18, m	44.6, CH
10		36.8, C		36.8, C
11	1.60, m 1.55, m	19.0, CH_2_	1.58, m	19.1, CH_2_
12	1.93, m 1.91, m	35.0, CH_2_	1.94, m 1.92, m	35.0, CH_2_
13		44.8, C		44.7, C
14		153.6, C		153.6, C
15	2.54, m	25.3, CH_2_	2.55, m	25.3, CH_2_
16	2.12, m 2.01, m	25.0, CH_2_	2.14, m 2.03, m	25.0, CH_2_
17	2.14, m	58.1, CH	2.14, m	58.2, CH
18	0.84, s	19.7, CH_3_	0.85, s	19.7, CH_3_
19	1.00, s	16.6, CH_3_	1.00, s	16.6, CH_3_
20		141.5, C		142.2, C
21	1.81, s	18.8, CH_3_	1.83, s	18.9, CH_3_
22	5.32, d (8.4)	124.1, CH	5.27, d (8.7)	123.6, CH
23	4.87, dd (8.4, 7.7)	81.4, CH	4.72, dd (8.7, 9.3)	81.1, CH
24	2.33, m	40.8, CH	1.80, m	46.1, CH
25	2.73, m	38.7, CH	2.21, m	43.0, CH
26	1.20, d (7.6)	10.4, CH_3_	1.24, d (7.0)	13.0, CH_3_
27		179.9, C		178.9, C
28	1.03, d (7.0)	12.2, CH_3_	1.10, d (6.5)	14.4, CH_3_

Penijanthoid B (**6**)

Colorless powder; [α]${}_{\text{D}}^{25}$ +263 (*c* 3.0, CH_2_Cl_2_); UV (MeOH) λ_max_ (log ε) 350 (0.86) nm; CD (MeOH) λ_max_ (Δε) 213 (1.4), 223 (−1.9), 230 (−2.3), 247 (−14.6), 283 (1.6), 358 (13.6) nm; IR (KBr) *v*_max_ 2,932, 2,249, 1,725, 1,650, 1,600, 1,468, 1,395, 1,176 cm^−1^; ^1^H and ^13^C NMR data, see Table 2; HRESIMS *m/z* 421.2732 [M + H]^+^ (calcd. for C_28_H_36_O_3_, 421.2737) (Figure S31).

### Computational Section

Conformational search of the new compounds **1**, **5**, and **6** for quantum calculations was taken using MMFF94S force field with low energetics from 0–10.0 kcal/mol. Optimization for these geometries were carried out in the gas phase at the B3LYP/6-311+G(d) level. The optimized conformations with the relative energy between 0 and 2.5 kcal/mol was selected for ECD calculations, which were computed at the B3LYP/6-311++G(2d,p) level (gas phase) (Zhu, 2009, 2015; Zhu et al., 2014). For the DP4plus applications of **5** and **6**, unshielding tensor values of the optimized conformers were computed at the mPW1PW91/6-311+G(d,p)//mPW1PW91/6-311+G(d,p) level in the gas phase. All of the quantum chemical calculations were performed using Gaussian 09 package (Frisch et al., 2009).

### X-Ray Crystallographic Study of PC-M6 (3)

The crystal of **3** was acquired from a mixed solvent of methanol and dichlorine in a refrigerator for 14 days. The detail X-ray diffraction data of single-crystal **3** were collected by Bruker Smart APEXII with the crystal system of Mo target. The wavelength of radiation is 0.71073 Å. The block crystals of **3** are monoclinic, space group C2 with cell dimensions *a* = 19.2301(8) Å, *b* = 7.0166(3) Å, *c* = 17.2255(8) Å, *V* = 2322.67(18) Å^3^, *Z* = 4, *F*_(000)_ = 912, and goodness of fit on *F*^2^ = 1.042. The Final R indices [*I* > *2*σ(*I*)] were *R1* = 0.0390, *wR2* = 0.0871. R indices (all data) were *R1* = 0.0524, *wR2* = 0.0951. The detail data of the crystal for **3** has been uploaded to the Cambridge Crystallographic Data Center. The relevant single crystal data can be viewed in the database and copies can be downloaded free of charge. The CCDC number for supplementary publication is NO. CCDC 1839742. CCDC's mailing address is as follows, 12 Union Road, Cambridge CB2 1EZ, U.K. (Fax, + 44(0)-1223–336033; email, deposit@ccdc.cam.ac.uk).

### Anti-*Vibrio* Activity Assays

The conventional broth dilution assay described by on the related literature (Appendio et al., 2008) was used to test the anti-*Vibrio* activity of these compounds. Three pathogenic *Vibrio* strains, *Vibrio anguillarum, Vibrio parahemolyticus* and *Vibrio alginolyticus* were incubated about 16–18 h at 37°C as the tested strains. The overnight cultures were used to prepare the turbidity of the bacterial suspensions, which had a concentration of 10^5^-10^6^ colony formingunits/mL and had the absorbance of 0.4–0.6 at 600 nm. The 96-well plates, which contained 2 μL of test solutions/positive control ciprofloxacin and 198 μL of bacterial culture, were used to test the minimum inhibitory concentration (MIC) of anti-*Vibrio* activity for these compounds. Finally, the different concentrations of tested compounds from 25.0 to 0.195 μM were prepared and incubated overnight for 24 h at 37°C to measure the MIC values of anti-*Vibrio* activity. Ciprofloxacin had the MIC values of 0.078, 0.312, and 0.625, respectively, against *V. anguillarum, V. parahemolyticus*, and *V. alginolyticus*.

## Results and Discussion

**Figure 2:**
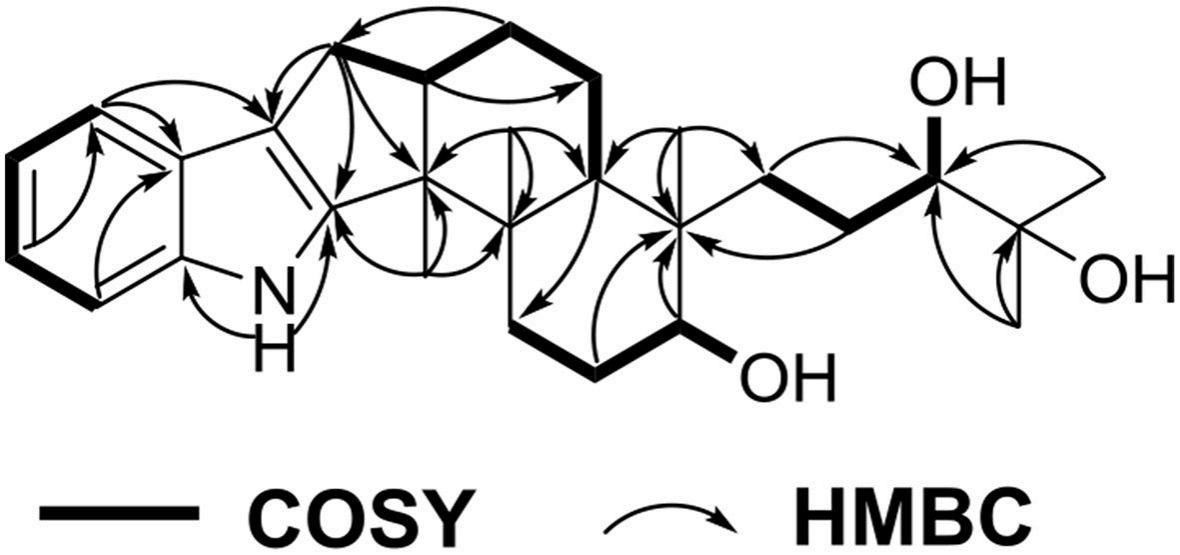
COZY and key HMBC correlations for **1**.

Penijanthine C (**1**) was isolated as a yellow amorphous powder. The molecular formula of C_28_H_41_NO_3_ for **1** was determined by high resolution mass spectrometry *m/z* = 440.3163 [M + H]^+^ (calcd. 440.3159) (Figure S10), suggesting nine degrees of unsaturation in **1**. In the ^13^C NMR spectroscopic data (Table 1) of **1**, 28 carbon signals which contain five methyls, seven methylenes, eight methines including four olefinic carbons, and two oxygen-bearing carbons, and eight quaternary carbons with four sp^2^ and two oxygenated sp^3^ were observed. The above ^13^C NMR signals agreed well with the ^1^H NMR spectroscopic data (Table 1) of **1**, which displayed a 1,2-disubstituted aromatic unit [δ_H_ 7.27 (2H, d, *J* = 7.8 Hz, H-16 and H-19), 6.94 (1H, dd, *J* = 7.8, 7.2 Hz, H-18), and 6.89 (1H, dd, *J* = 7.8, 7.2 Hz, H-17)], two oxymethine protons [δ_H_ 3.34 (1H, m, H-7) and 3.00 (1H, t, *J* = 7.2 Hz, H-26)], and five methyls [δ_H_ 1.08 (3H, s, H-29), 1.03 (3H, s, H-28), 1.02 (3H, s, H-22), 0.94 (3H, s, H-21), and 0.75 (3H, s, H-23)]. The above characteristic ^1^H and ^13^C NMR data of **1** suggested an indole-diterpenoid framework for **1**. In fact, **1** could be identified as an indole-diterpenoid analog of emindole SB, which was previously isolated from the fungus *Penicillium camemberti* (Fan et al., 2013), by careful comparison of their 1D NMR data. The structural difference between them was that the 26,27-trisubstituted double bond in emindole SB was replaced by a *vic*-diol moiety [δ_H_ 3.00 (1H, t, *J* = 7.2 Hz, H-26); δ_C_ 78.6 (CH, C-26) and 71.7 (C, C-27)] in **1** (Figures S4, S5). The long-range couplings of H_3_-28/C-26, H_3_-28/C-27, H_3_-29/C-26, H_3_-29/C-27, and H_2_-24/C-26 in the HMBC spectrum of **1**, as well as the proton spin system of H_2_-25/H-26 from the ^1^H-^1^H COZY spectrum in **1**, supported the above deduction (Figure 2). The assignment of the planar structure for **1** was consequently confirmed by the 2D NMR data of HSQC, ^1^H-^1^H COZY, and HMBC in **1** (Figures S6–S8).

**Figure 3:**
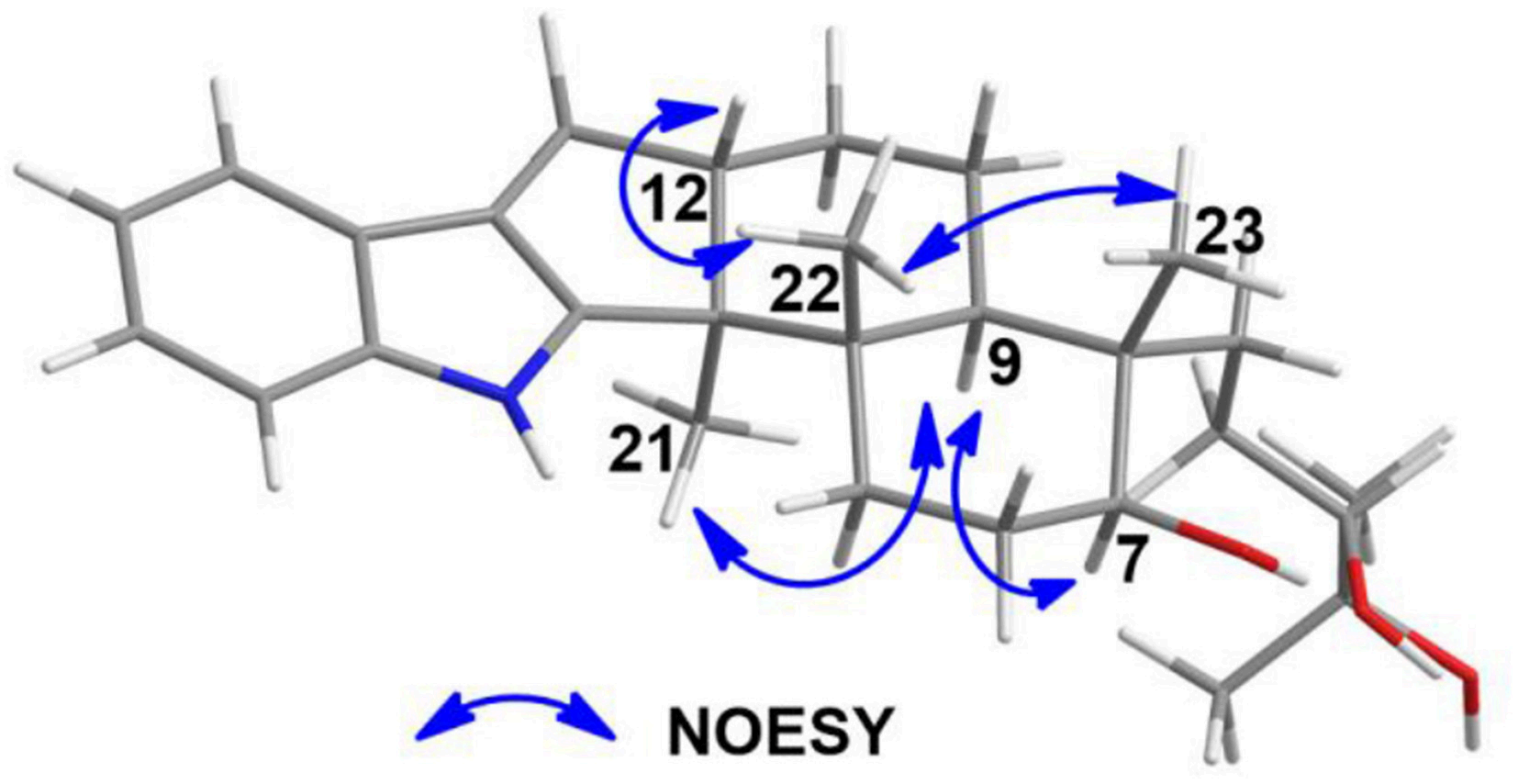
Selected NOESY correlations for **1**.

The analysis of the NOESY data allowed the relative configuration of the five rings for the indole-diterpenoid nuclei in **1**. The NOESY cross-peaks between the H-12 and H_3_-22, H_3_-22, and H_3_-23 as well as H-9 had NOE with both H_3_-21 and H-7 were observed in the NOESY experiment of **1**, suggesting that H-12, H_3_-22, and H_3_-23 were place on the opposite direction to H-7, H-9, and H_3_-21 in the molecule of **1** (Figure 3). However, the NOESY experiment was unable to conclusively determine the configuration at C-26 in **1** (Figure S9).

**Figure 4:**
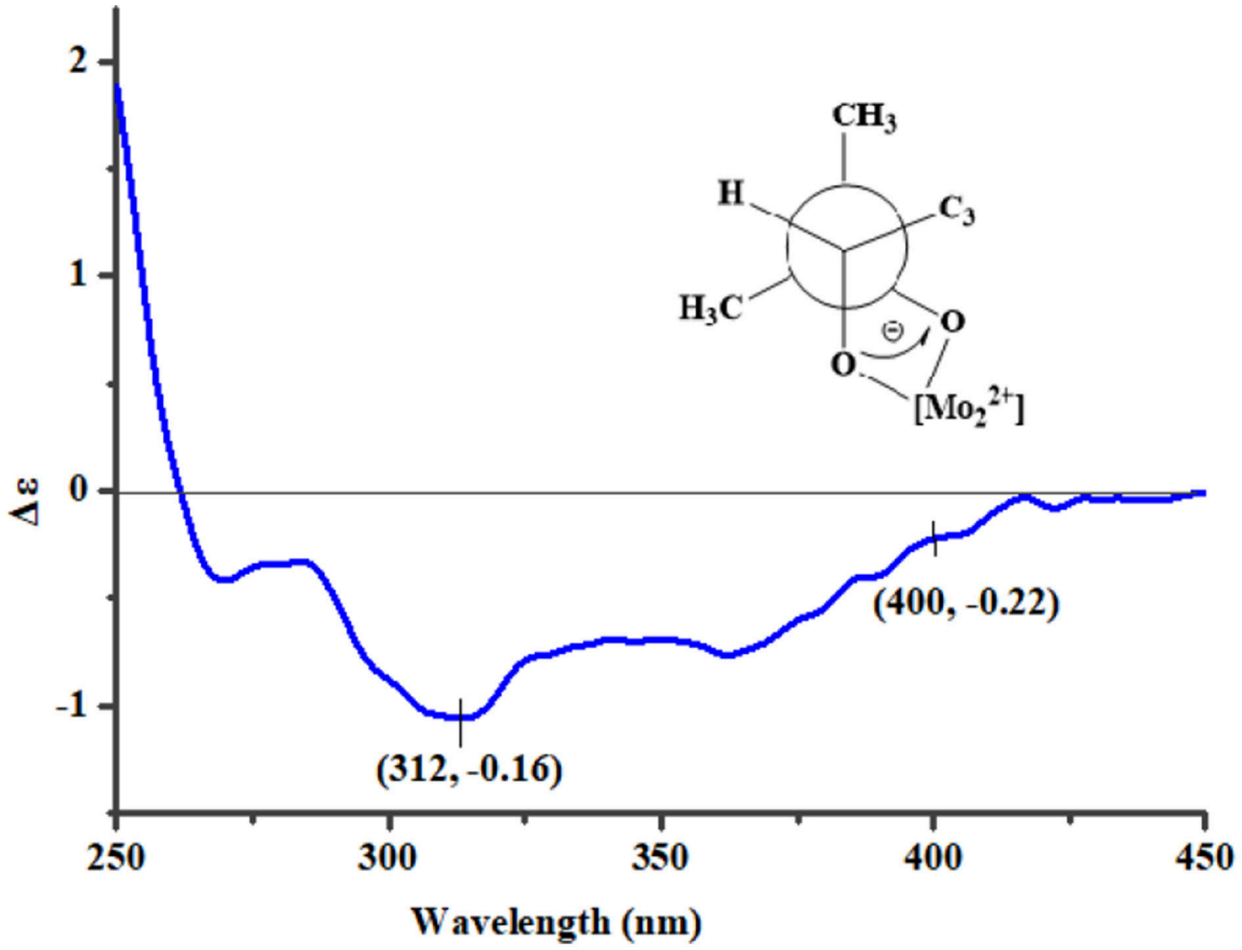
The ICD spectrum of Mo-complexes of **1**.

**Figure 5:**
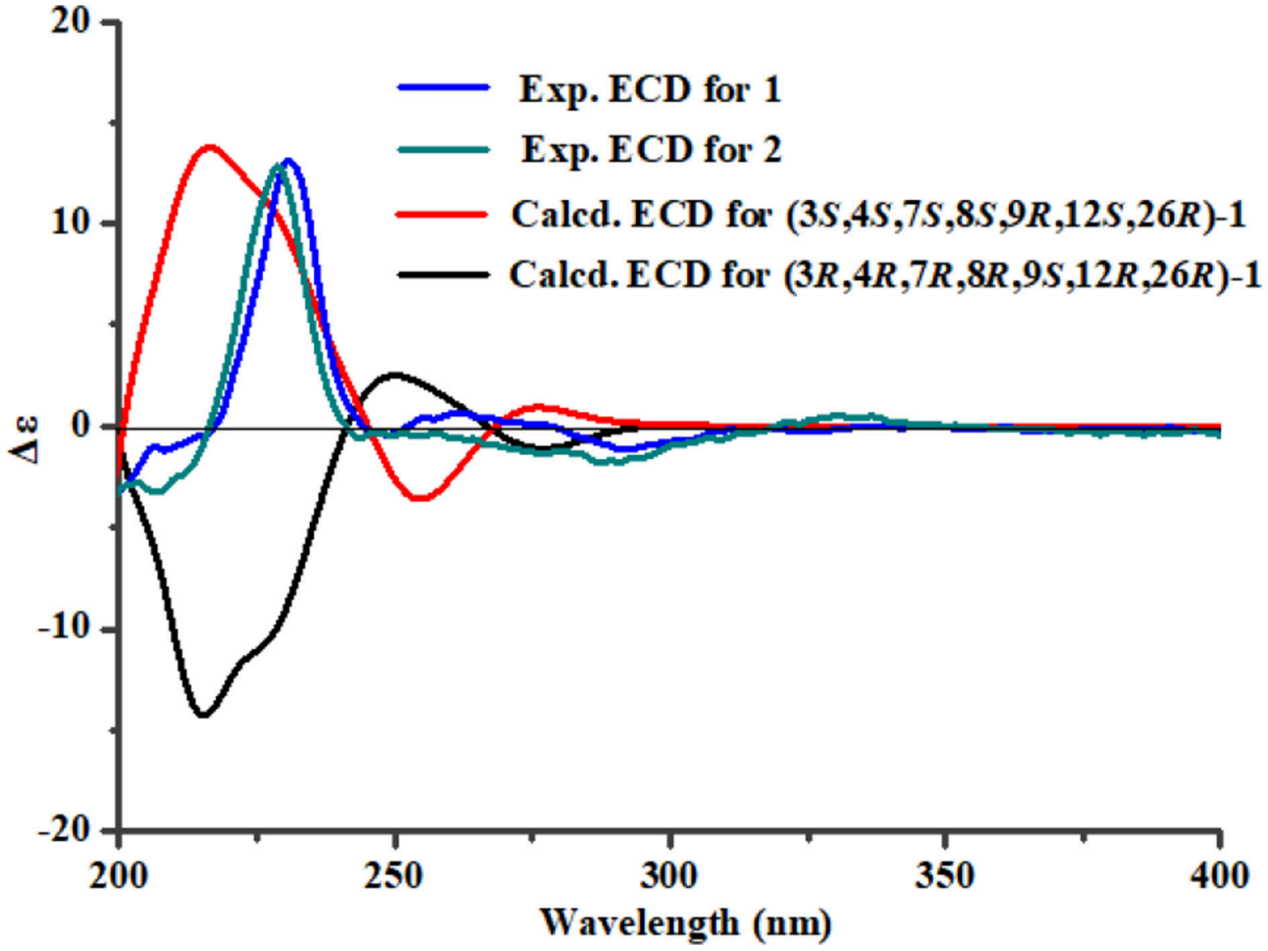
The predicted ECD spectra of **1** and the experimental ECD spectra of **1** and **2**.

To assign the stereochemistry of 26-stereogenic carbon in **1**, induced circular dichroism (ICD) procedure (Snatzke's method) was applied (Frelek et al., 1997; Di Bari et al., 2001). The Mo-complexes of compound **1** (0.5 mg) and Mo_2_(OAc)_4_ (dimolybdenum tetraacetate) was prepared to measure the its ICD spectrum. Compared with the ICD spectrum of the reference Mo_2_ complex (Frelek et al., 1997; Di Bari et al., 2001), the Cotton effect bands II (near 400 nm) and IV (around 312 nm) in the ICD data of Mo-complexes of **1** were negative (Figure 4), suggesting the 26*R* absolute configuration for **1**. The absolute configuration of the five rings in the indole-diterpenoid nuclei of **1** was investigated by quantum chemical calculation. Based on the relative configuration of **1**, two possible structures of (3*S*,4*S*,7*S*,8*S*,9*R*,12*S*,26*R*)-**1** and (3*R*,4*R*,7*R*,8*R*,9*S*,12*R*,26*R*)-**1** of **1** were used for ECD calculations. Time-dependent density functional theory/electronic circular dichroism (TDDFT-ECD) method at the B3LYP/6-311++G(2d,p)//B3LYP/6-311+G(d) level in the gas phase was taken. ECD simulations were calculated by Boltzmann statistics for the structures of (3*S*,4*S*,7*S*,8*S*,9*R*,12*S*,26*R*)-**1** and (3*R*,4*R*,7*R*,8*R*,9*S*,12*R*,26*R*)-**1** with a standard deviation of σ 0.2 eV. The calculated ECD curve of (3*S*,4*S*,7*S*,8*S*,9*R*,12*S*,26*R*)-**1** agreed better with the experimental ECD data of **1** (Figure 5), indicating an obvious assignment of 3*S*,4*S*,7*S*,8*S*,9*R*,12*S*,26*R* absolute configuration for **1**.

Penijanthine D (**2**) had the molecular formula of C_30_H_43_NO_4_, which was determined by the high resolution mass data *m/z* = 504.3072 [M + H]^+^ (calcd. 504.3084) of **2** (Figure S17). Compound **2** was also defined as an indole-diterpenoid analog by the strikingly similar NMR data of **2** (Figures S11–S15) compared with those of **1** (Table 1), with the appearance of the additional acetoxy signals [δ_H_ 1.94 (3H, s); δ_C_ 169.9 and 21.0] in **2**. This additional acetoxy group was connected at C-7 in **2** was through the key HMBC correlation between H-7 and C-*C*OCH_3_. The NOESY (Figure S16) and ECD (Figure 5) experiments of **2** indicated the stereochemistry of **2** was the same as **1**. Therefore, compound **2** was assigned as the 7-acetylation derivative of **1**.

**Figure 6:**
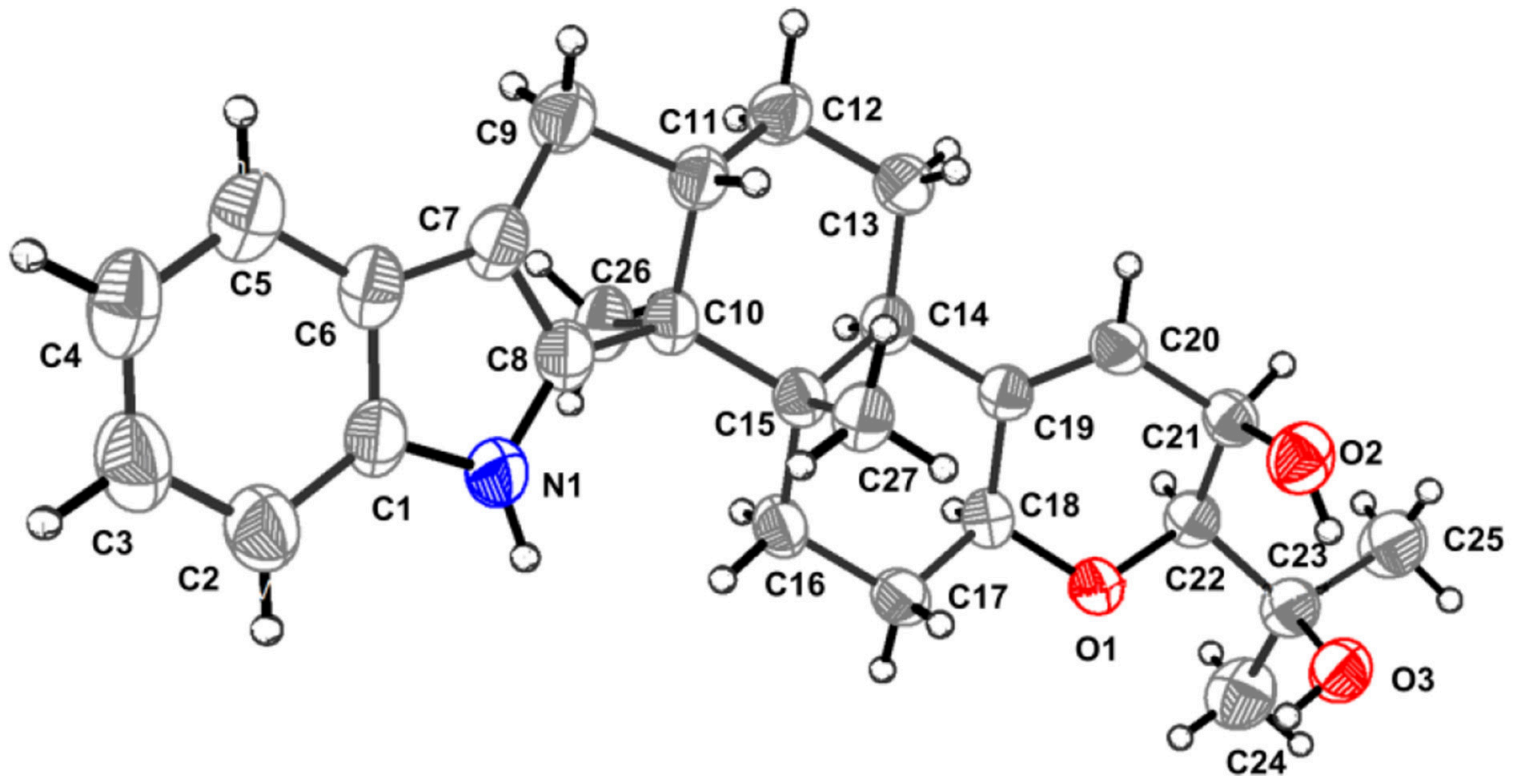
Perspective ORTEP drawing for **3**.

The known PC-M6 (**3**) (Yamaguchi et al., 1993) and 7-hydroxy-13-dehydroxypaxilline (**4**) (Mantle and Weedon, 1994) were determined by comparing their ^1^H, ^13^C NMR and positive Mass data with the corresponding data in the literature. The stereochemistry of PC-M6 (**3**) was further verified by the data of X-ray diffraction (Figure 6) and calculated ECD (Figure S1) for the first time. The present work affords four indole-diterpenoids (**1**–**4**), which consist of a common cyclic diterpene backbone and an indole moiety. According to the literature, over 100 indole-diterpenoids with unique chemical scaffolds were produced by various fungal sources (Li et al., 2002; Zhao et al., 2018). The complexity of these intriguing structures may encourage further investigations on the chemistry and biological activity of this cluster of metabolites.

**Figure 7:**
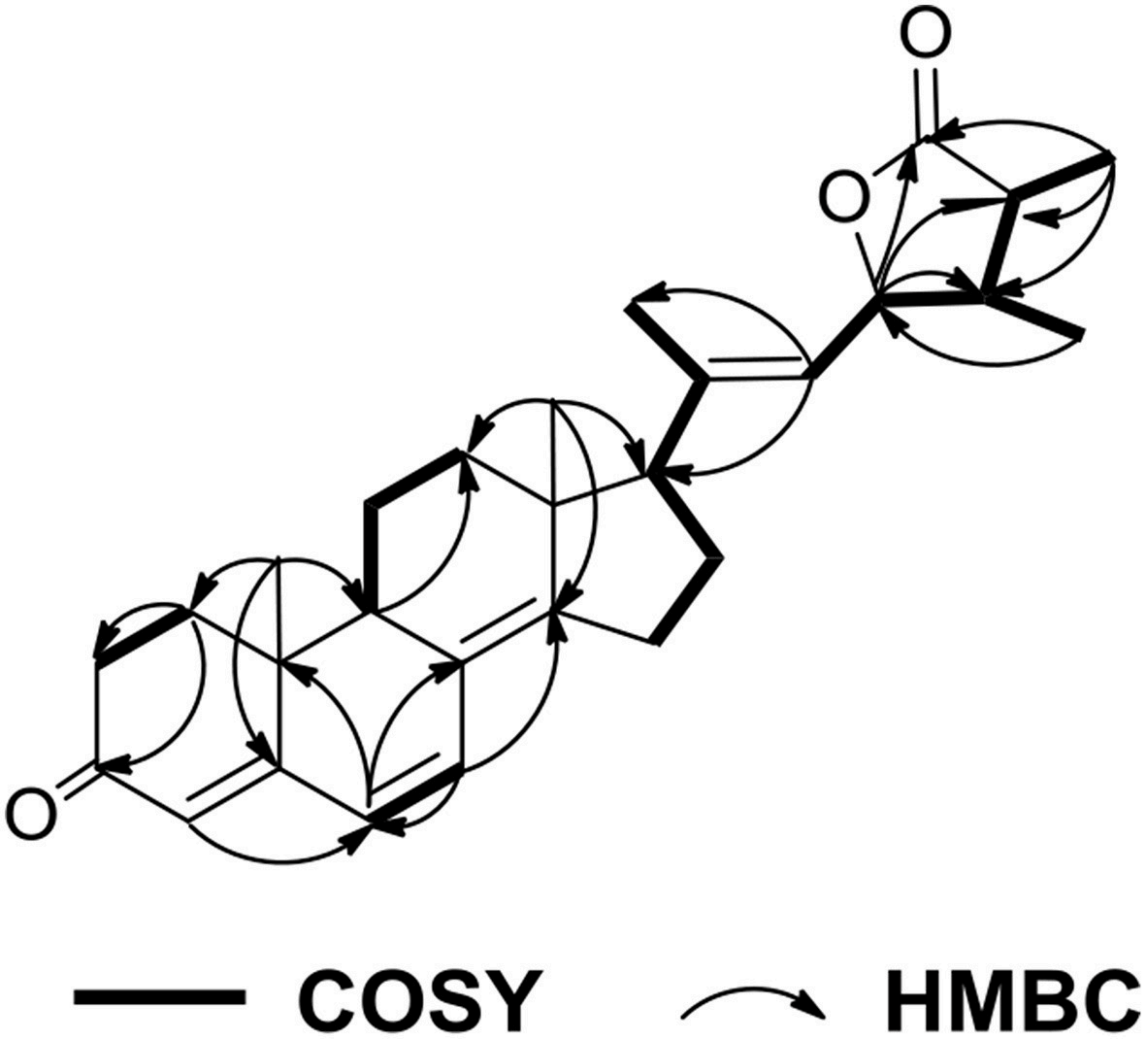
COZY and key HMBC correlations for **5**.

Penijanthoid A (**5**), which had the molecular formula of C_28_H_36_O_3_, was also isolated as a colorless powder. According to the ^1^H NMR spectrum of **5** (Table 2), the downfield region gave four olefinic protons [δ_H_ 6.60 (1H, d, *J* = 9.5 Hz, H-7), 6.06 (1H, d, *J* = 9.5 Hz, H-6), 5.75 (1H, s, H-4), and 5.32 (1H, d, *J* = 8.4 Hz, H-22)], the highfield region displayed five CH_3_ signals [δ_H_ 1.81 (1H, s, H-21), 1.20 (1H, d, *J* = 7.6 Hz, H-26), 1.03 (1H, d, *J* = 7.0 Hz, H-28), 1.00 (1H, s, H-19), and 0.84 (1H, s, H-18)] and numerous CH_2_ and CH signals in the range of δ_H_ 2.80–1.25 ppm. The above spectroscopic features suggested that **5** was a steroidal derivative, which was structural similar to the known steroid (22*E*)-ergosta-4,6,8(14),22-tetraen-3-one (Chen et al., 2015). In fact, the main difference between compound **5** and (22*E*)-ergosta-4,6,8(14),22-tetraen-3-one was located on the side chains of them. Then, a five-membered lactone ring was proven to be present in the side chain of **5** by the HMBC correlations from H-23 (δ_H_ 4.87) and H_3_-26 (δ_H_ 1.20) to C-27 (δ_C_ 179.9), C-24 (δ_C_ 40.8), C-25 (δ_C_ 38.7), and from H_3_-28 (δ_H_ 1.03) to C-23 (δ_C_ 81.4), and the COSY (Figures S18–S22) cross-peaks of H-22/H-23/H-24/H-25/H_3_-26 in **5** (Figure 7).

**Figure 8:**
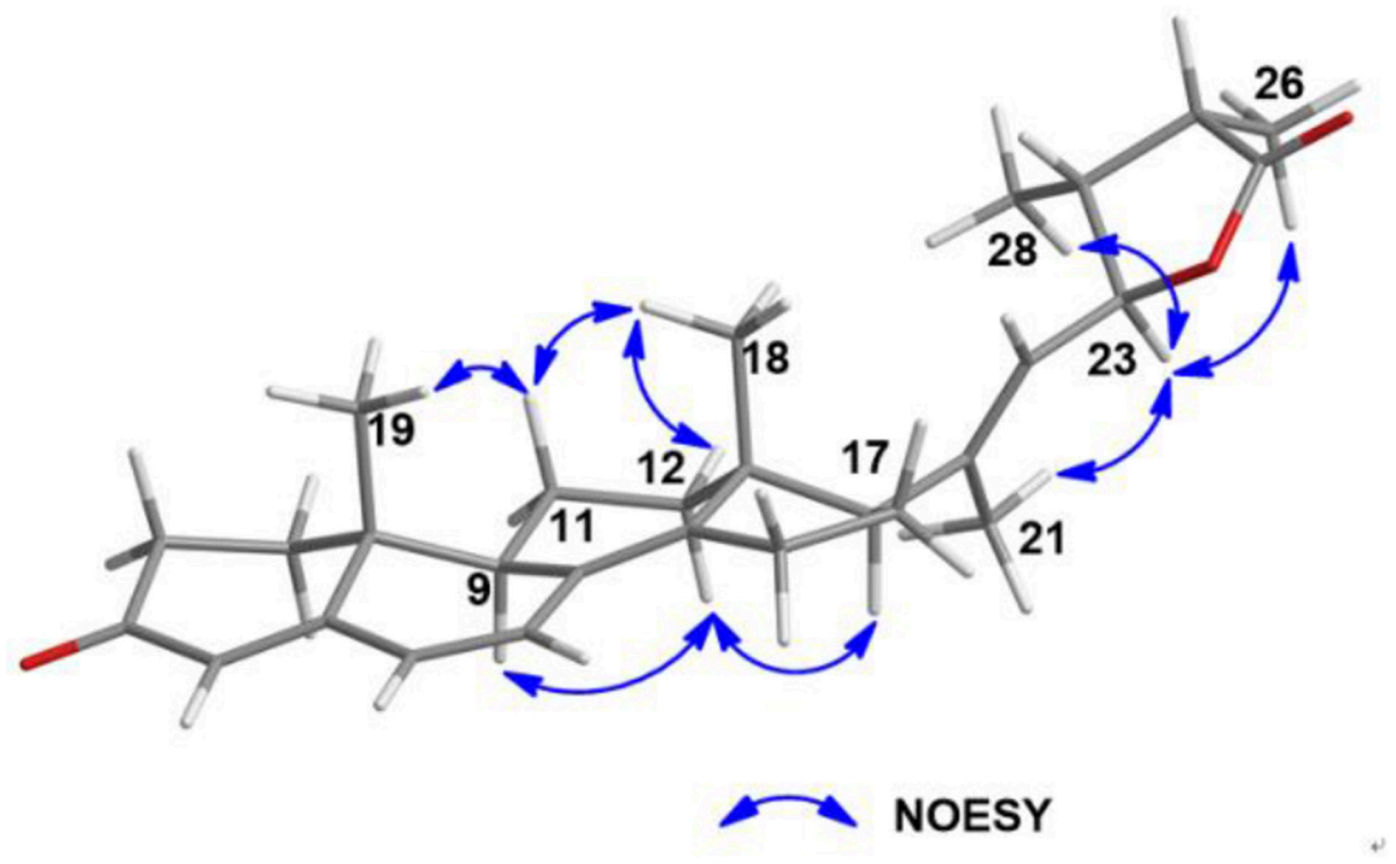
Selected NOESY correlations for **5**.

Further, NOESY experiment of **5** (Figure 8) was used to define its relative configuration. The NOE correlation between H_3_-21 and H-23 in the NOESY spectrum of **5** indicated the *E* orientational double bond between C-20 and C-22 in **5**. The NOESY cross-peaks of H-11α*/*H_3_-18, H-11α*/*H_3_-19, H-12β*/*H_3_-18, H-12α*/*H-9, and H-12α*/*H-17 suggested that **5** had the same relative configuration in tetracyclic nucleus as that of the compound (22*E*)-ergosta-4,6,8(14),22-tetraen-3-one in the literature (Chen et al., 2015). Furthermore, the NOE interactions of H-23/H_3_-26 and H-23/H_3_-28 indicated the same orientation of these protons (Figure S23). Besides, the carbonyl of cyclohexanone mainly contributed to the positive ECD effect at 363 nm (Δε + 24.3) (Figure S2) of **5**. By applying octant rule to cyclohexanones (Ochi et al., 1991), the absolute configuration of tetracyclic nucleus in **5** could be assigned as 9*R*,10*R*,13*R*,17*R*. In order to further determine the absolute configuration of five-membered lactone ring in **5**, two possible absolute configurations of **5**, [(9*R*,10*R*,13*R*,17*R*,23*S*,24*S*,25*R*)-**5** and (9*R*,10*R*,13*R*,17*R*,23*R*,24*R*,25*S*)-**5**], were used for GIAO NMR shift calculations at the mPW1PW91/6-311+G(d,p) level in the gas phase. When the parameter of DP4plus probability was used (Grimblat et al., 2015), the configuration of (9*R*,10*R*,13*R*,17*R*,23*S*,24*S*,25*R*)-**5** was more likely than (9*R*,10*R*,13*R*,17*R*,23*R*,24*R*,25*S*)-**5** (97.6 *vs*. 2.4% in both the unscaled shift data and shielding tensor data) (Figure S32). Therefore, the absolute configuration of **5** was suggested to be (9*R*,10*R*,13*R*,17*R*,23*S*,24*S*,25*R*).

Penijanthoid B (**6**) was also obtained with the same molecular formula of C_28_H_36_O_3_ as **5**, indicating that **5** and **6** may be a pair of epimers. The above deduction was further confirmed by the fact that the NMR data of **6** were almost the same as **5** (Table 2) and the detailed analysis of the HSQC, ^1^H-^1^H COZY and HMBC spectra of **6** (Figures S25–S29). The NOESY (Figure S30) correlations between H-23 and H_3_-21/H-25/H_3_-28 demonstrated that **6** was the C-25 epimer of **5**. The absolute configuration (9*R*,10*R*,13*R*,17*R*,23*S*,24*S*,25*S*) of **6** was also assigned by ECD spectrum (Figure S3) and DP4plus (Figure S33) approaches. Among the various classes of biologically active natural produces obtained from marine-derived fungi, a large number of steroidal compounds have been described (Gautschi et al., 2004; Zhang et al., 2007; Wang et al., 2008; Qiao et al., 2010). However, **5** and **6** represent the first examples of steroids forming a five-membered lactone between C-23 and C-27 from marine fungi.

Vibriosis, which is also known as bacterial canker, is one of the bacterial diseases which cause serious damage and great losses to mariculture production (Vezzulli et al., 2015; Moreno et al., 2017). Research and development of effective anti-*Vibrio* drugs for controlling vibriosis is needed for mariculture. Thus, the anti-*Vibrio* activities against *V. anguillarum, V. parahemolyticus*, and *V. alginolyticus* of the new compounds **1**, **2**, **5**, **6** were carried out. Compound **1** displayed strongest anti-*Vibrio* activity against *V. Anguillarum* (MIC = 3.1 μM), *V. parahemolyticus* (MIC = 6.3 μM), and *V. Alginolyticus* (MIC = 3.1 μM), respectively. Compound **2** showed moderate anti-*Vibrio* activity against three pathogenic *Vibrio* spp. with the same MIC values of 12.5 μM, suggesting that the presence of an acetoxy group at C-7 in **2** may decrease the anti-*Vibrio* activity. A literature survey showed that the other known indole diterpenoid analogs, such as 6-hydroxylpaspalinine, paspalitrem C, emindole SB and so on, were also showed anti-*Vibrio* activity against three pathogenic *Vibrio* spp. (Hu et al., 2017). These finding suggested that it was worth ongoing to seek new anti-*Vibrio* compunds from indole diterpenoid derivatives. However, compounds **5** and **6** only exhibited weak anti-*Vibrio* activity against three pathogenic *Vibrio* spp. (MICs, 25.0–50.0 μM).

## Conclusion

Four indole-diterpenoids and two steroidal epimers were isolated from the marine-derived fungus *Penicillium janthinellum*. Snatzke's, X-ray diffraction, and calculated ECD methods were used to assign the absolute configurations of these compounds. The absolute configurations of steroidal epimers were suggested by DP4plus approach. Compounds **1** and **2** exhibited potential anti-*Vibrio* activity and represented a promising new class of anti-*Vibrio* agents.

## Author Contributions

L-LX and R-YY: contribute to fermentation, extraction, and isolation. X-CG: contribute to manuscript preparation. M-YY and L-DH: contribute to quantum chemistry calculation and bioactivities test. H-JZ and FC: were the project leaders organizing and guiding the experiments and manuscript writing.

### Conflict of Interest Statement

The authors declare that the research was conducted in the absence of any commercial or financial relationships that could be construed as a potential conflict of interest.
