# Supplementary material for: Anti-Vibrio Indole-Diterpenoids and C-25 Epimeric Steroids From the Marine-Derived Fungus Penicillium janthinellum
Source: Front Chem. 2019 Feb 15;7:80. doi: 10.3389/fchem.2019.00080 (PMC6413715; doi:10.3389/fchem.2019.00080)

**Anti-*Vibrio* Indole-Diterpenoids and C-25 Epimeric Steroids from the Marine-Derived Fungus *Penicillium janthinellum***

Xing-Chen Guo^1†^, Lan-Lan Xu^1†^, Rui-Yun Yang^2^, Meng-Yue Yang^1^, Lian-Dong Hu^1^, Fei Cao^1*^, and Hua-Jie Zhu^1*^

^1^Key Laboratory of Pharmaceutical Quality Control of Hebei Province, Key Laboratory of Medicinal Chemistry and Molecular Diagnostics of Education Ministry of China, College of Pharmaceutical Sciences, Hebei University, Baoding 071002, People’s Republic of China.

^2^State Key Laboratory for Chemistry and Molecular Engineering of Medicinal Resources, College of Chemistry and Pharmaceutical Sciences, Guangxi Normal University, Guilin 541004, China.

* To whom correspondence should be addressed. E-mail: caofei542927001@163.com (F. Cao); zhuhuajie@hotmail.com (H. J. Zhu)

**Lists of Supplementary Materials**

**Fig. S1** The predicted ECD spectrum and its experimental ECD spectrum of **3**.

**Fig. S2** The experimental ECD spectrum of **5**.

**Fig. S3** The experimental ECD spectrum of **6**.

**Fig. S4** ^1^H NMR (600 MHz, DMSO-*d*_6_) spectrum of **1**.

**Fig. S5** ^13^C and DEPT NMR (150 MHz, DMSO-*d*_6_) spectra of **1**.

**Fig. S6** HSQC (DMSO-*d*_6_) spectrum of **1**.

**Fig. S7** ^1^H-^1^H COSY (DMSO-*d*_6_) spectrum of **1**.

**Fig. S8** HMBC (DMSO-*d*_6_) spectrum of **1**.

**Fig. S9** NOESY (DMSO-*d*_6_) spectrum of **1.**

**Fig. S10** HRESIMS for compound **1**.

**Fig. S11** ^1^H NMR (600 MHz, DMSO-*d*_6_) spectrum of **2**.

**Fig. S12** ^13^C NMR (150 MHz, DMSO-*d*_6_) spectrum of **2**.

**Fig. S13** HSQC (DMSO-*d*_6_) spectrum of **2.**

**Fig. S14** ^1^H-^1^H COSY (DMSO-*d*_6_) spectrum of **2.**

**Fig. S15** HMBC (DMSO-*d*_6_) spectrum of **2.**

**Fig. S16** NOESY (DMSO-*d*_6_) spectrum of **2.**

**Fig. S17** HRESIMS for compound **2.**

**Fig. S18** _1_H NMR (600 MHz, CDCl_3_) spectrum of compound **5.**

**Fig. S19** _13_C NMR (150 MHz, CDCl_3_) spectrum of compound **5.**

**Fig.S20** HSQC (CDCl_3_) spectrum of compound **5.**

**Fig. S21** ^1^H-^1^H COSY (CDCl_3_) spectrum of compound **5.**

**Fig. S22** HMBC (CDCl_3_) spectrum of compound **5.**

**Fig. S23** NOESY (CDCl_3_) spectrum of compound **5** and Local enlarged image.

**Fig. S24** HRESIMS spectrum of **5**.

**Fig. S25** _1_H NMR (600 MHz, CDCl_3_) spectrum of compound **6**.

**Fig. S26** _13_C NMR (150 MHz, CDCl_3_) spectrum of compound **6**.

**Fig. S27** HSQC (CDCl_3_) spectrum of compound **6**.

**Fig. S28** ^1^H-^1^H COSY (CDCl_3_) spectrum of compound **6**.

**Fig. S29** HMBC (CDCl_3_) spectrum of compound **6**.

**Fig. S30** NOESY (CDCl_3_) spectrum of compound **6** and Local enlarged image.

**Fig. S31** HRESIMS spectrum of **6**.

**Fig. S32** The data of DP4+ method of compound **5** (mPW1PW91/6-311+G(d,p)).

**Fig. S33** The data of DP4+ method of compound **6** (mPW1PW91/6-311+G(d,p)).

**Calculation details section**

1 Lowest energy conformers of compound (3*S*,4*S*,7*S*,8*S*,9*R*,12*S*,26*R*)-**1**.

2 Lowest energy conformers of compound (3*R*,4*R*,7*R*,8*R*,9*S*,12*R*,26*R*)-**1**.

3 Lowest energy conformers of compound (3*S*,4*S*,7*S*,9*S*,10*R*,13*R*,16*S*)-**3**.

4 Lowest energy conformers of compound (23*S*, 25*S*, 26*R*)-**5** for NMR calculation.

5 Lowest energy conformers of compound (23*R*, 25*R*, 26*S*)-**5** for NMR calculation.

6 Lowest energy conformers of compound (23*R*, 25*R*, 26*R*)-**6** for NMR calculation.

7 Lowest energy conformers of compound (23*S*, 25*S*, 26*S*)-**6** for NMR calculation.

**Checkcif Report for Compound 3**

Page1 and 2

**Supplementary Figures**


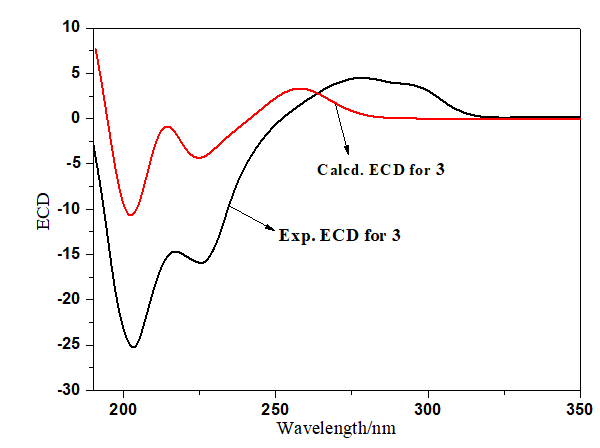


**Fig. S1** The predicted ECD spectrum and its experimental ECD spectrum of **3**.


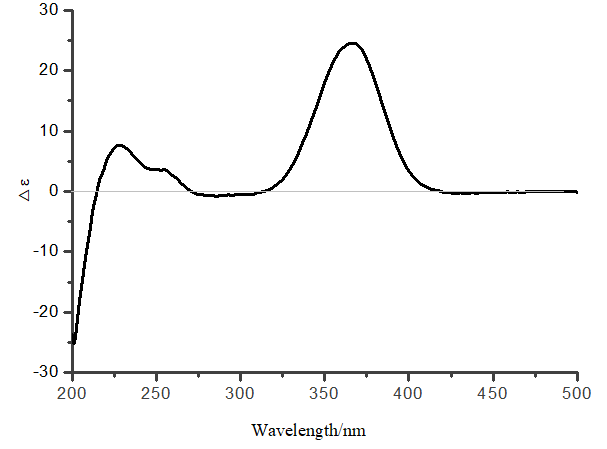


**Fig. S2** The experimental ECD spectrum of **5**.


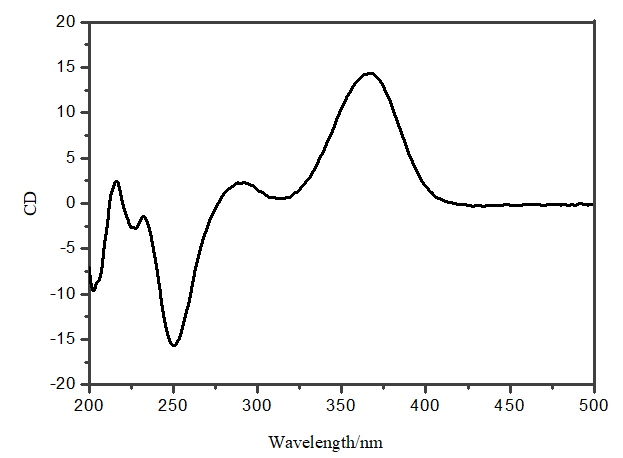


**Fig. S3** The experimental ECD spectrum of **6**.


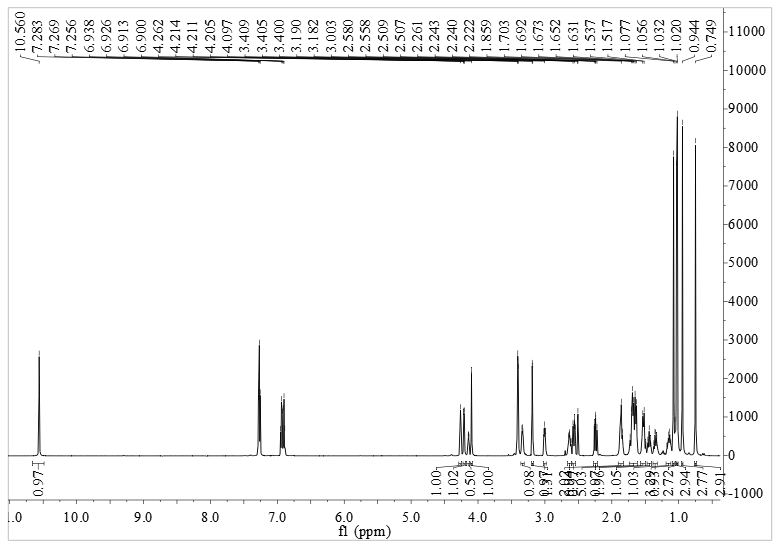


**Fig. S4** ^1^H NMR (600 MHz, DMSO-*d*_6_) spectrum of **1**.


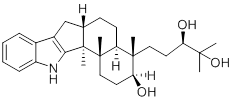

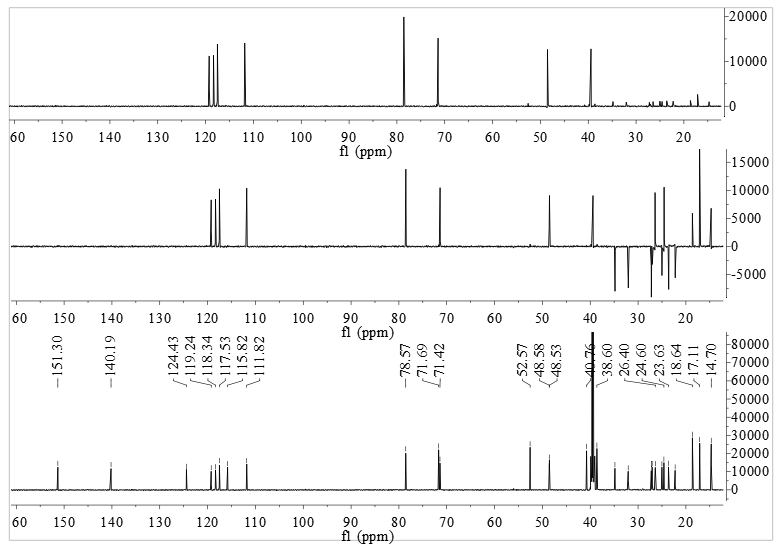


**Fig. S5** ^13^C and DEPT NMR (150 MHz, DMSO-*d*_6_) spectra of **1**.


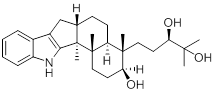

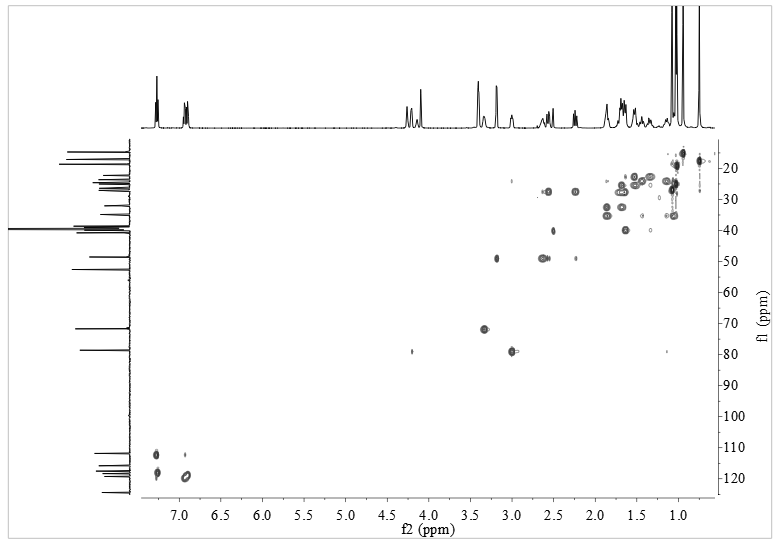


**Fig. S6** HSQC (DMSO-*d*_6_) spectrum of **1**.


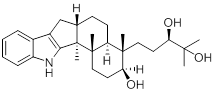

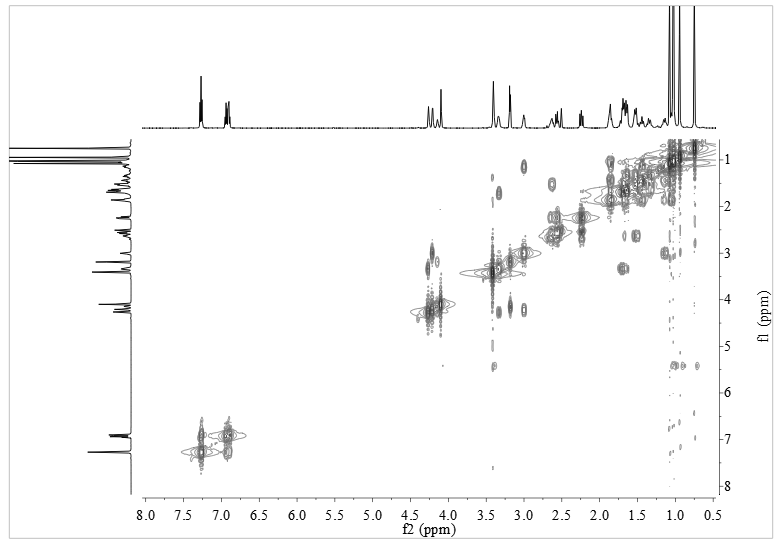


**Fig. S7** ^1^H-^1^H COSY (DMSO-*d*_6_) spectrum of **1**.


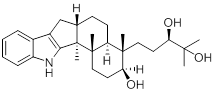

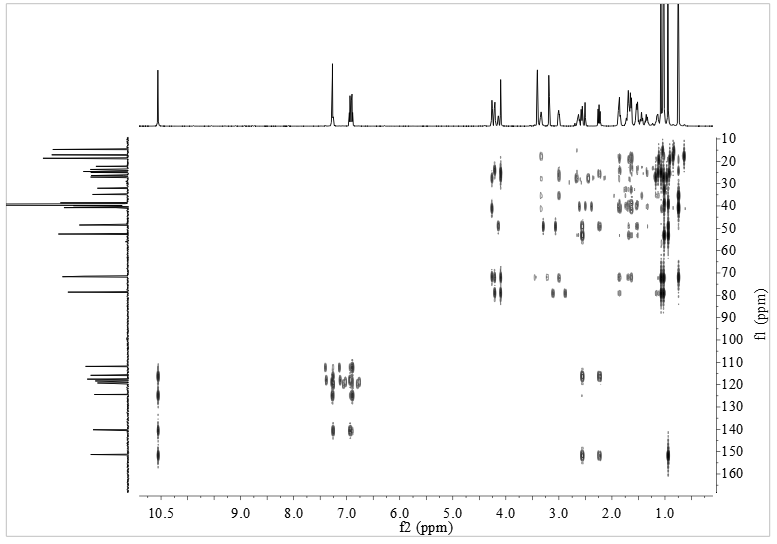


**Fig. S8** HMBC (DMSO-*d*_6_) spectrum of **1**.


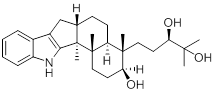

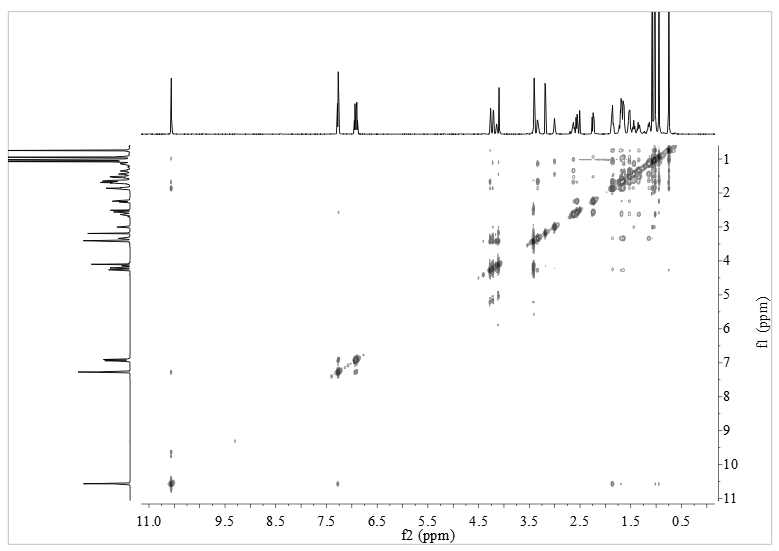


**Fig. S9** NOESY (DMSO-*d*_6_) spectrum of **1.**


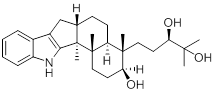

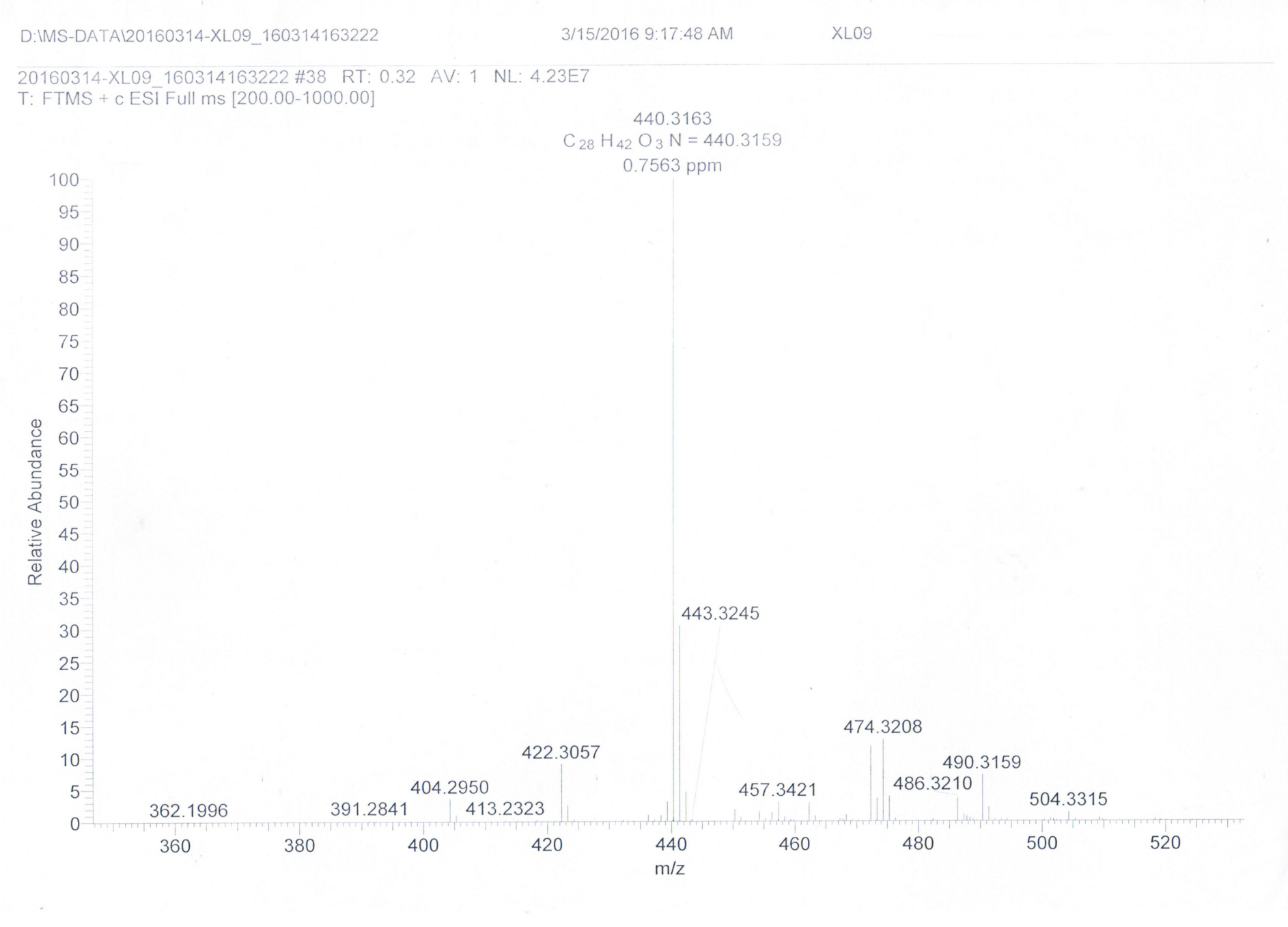


**Fig. S10** HRESIMS for compound **1**.


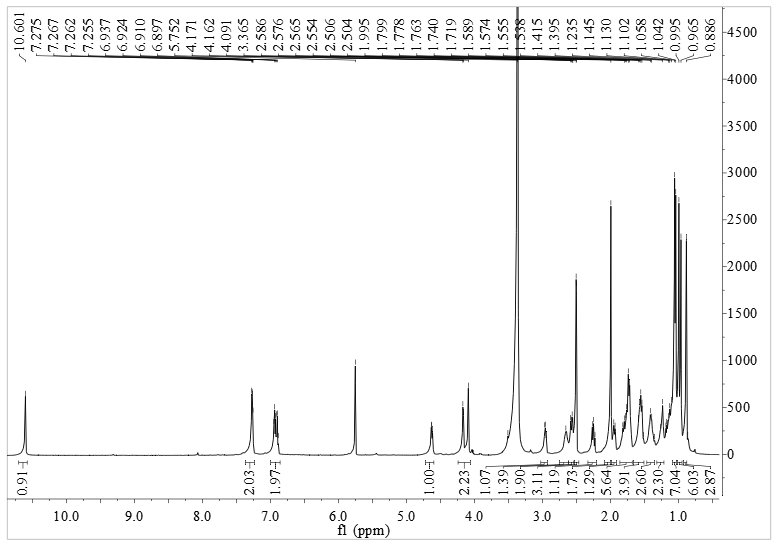


**Fig. S11** ^1^H NMR (600 MHz, DMSO-*d*_6_) spectrum of **2**.


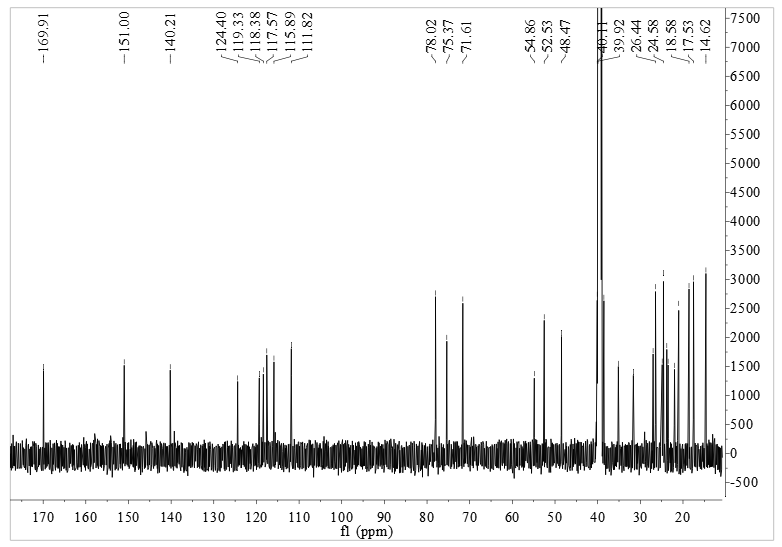


**Fig. S12** ^13^C NMR (150 MHz, DMSO-*d*_6_) spectrum of **2**.


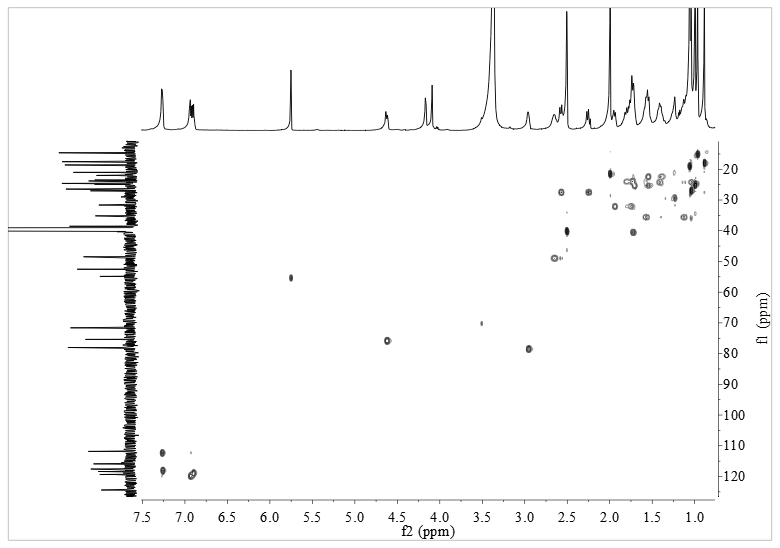


**Fig. S13** HSQC (DMSO-*d*_6_) spectrum of **2.**


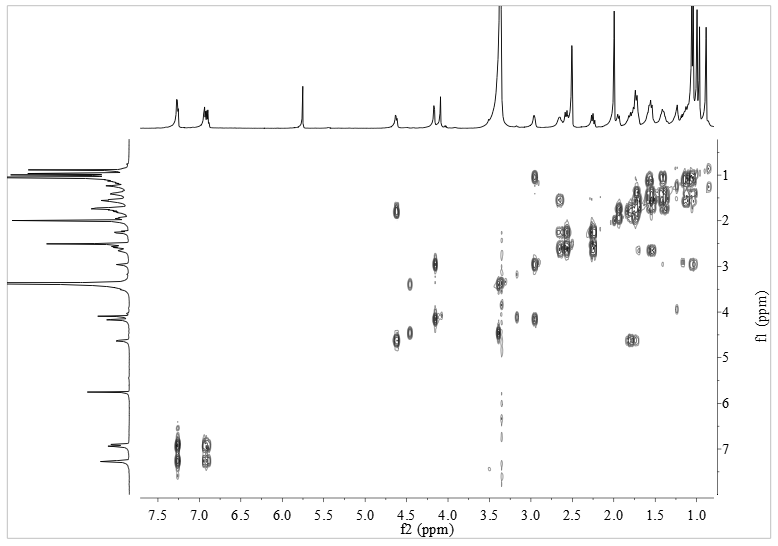


**Fig. S14** ^1^H-^1^H COSY (DMSO-*d*_6_) spectrum of **2.**


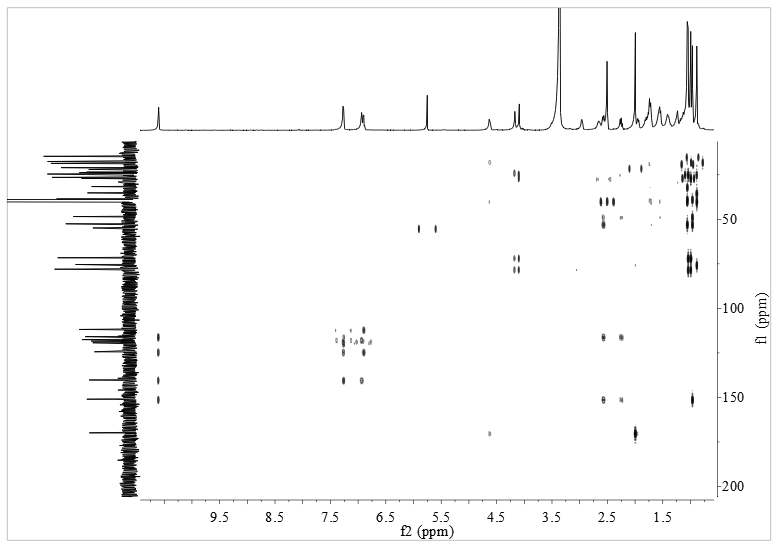


**Fig. S15** HMBC (DMSO-*d*_6_) spectrum of **2.**


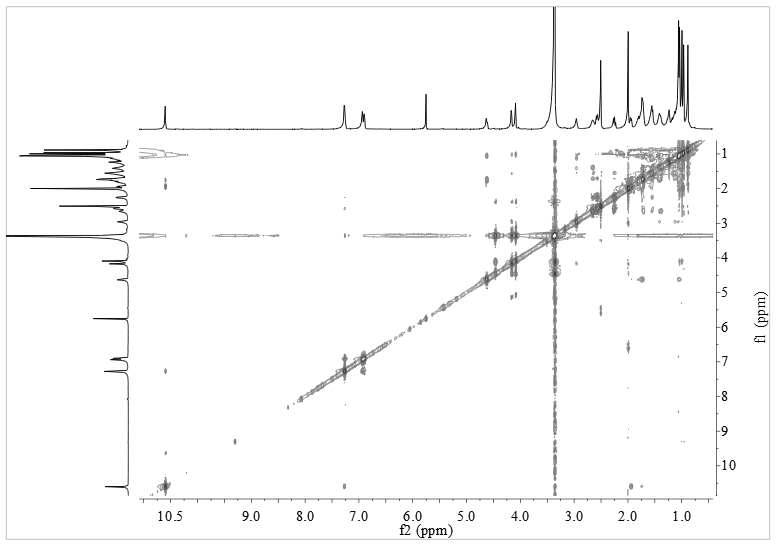


**Fig. S16** NOESY (DMSO-*d*_6_) spectrum of **2.**

**Fig. S17** HRESIMS for compound **2.**


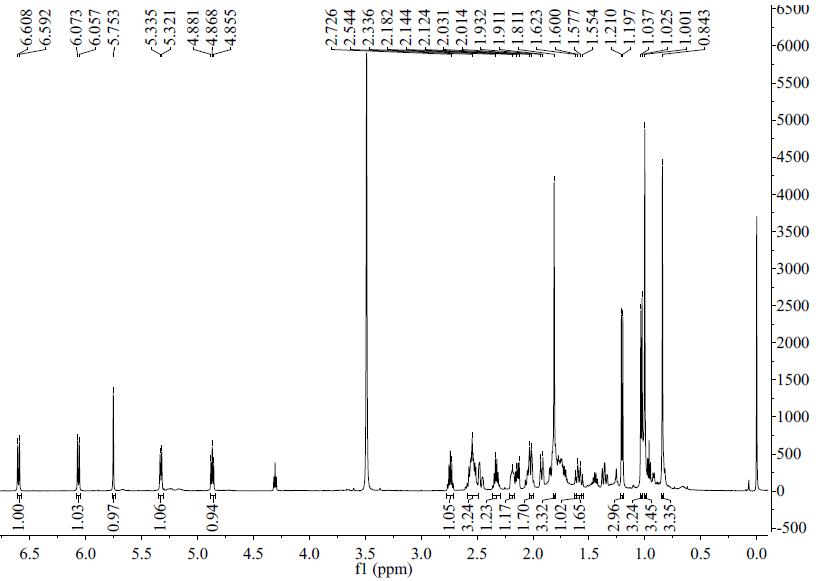


**Fig. S18** _1_H NMR (600 MHz, CDCl_3_) spectrum of compound **5.**


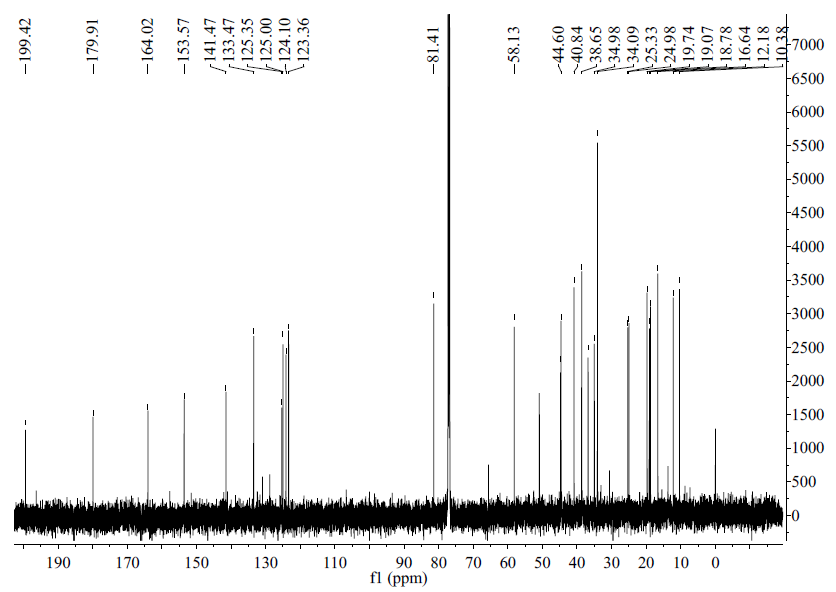


**Fig. S19** _13_C NMR (150 MHz, CDCl_3_) spectrum of compound **5.**


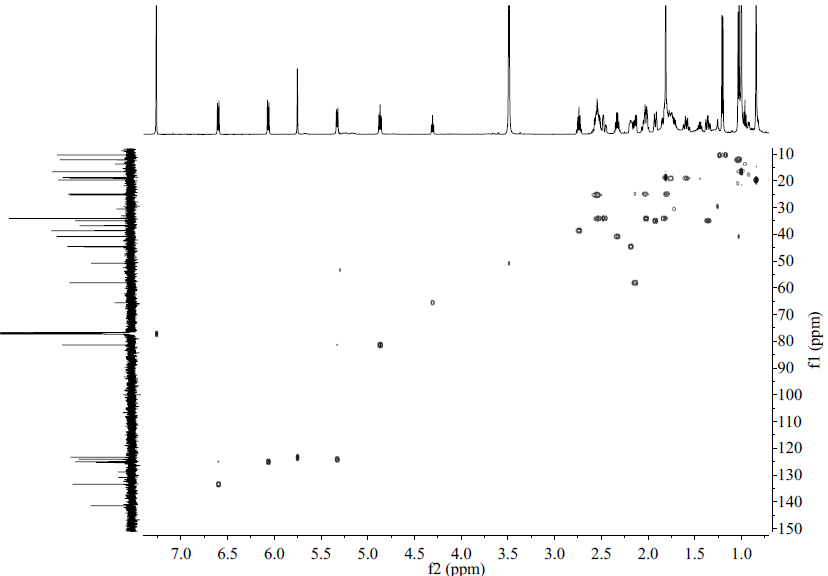


**Fig.S20** HSQC (CDCl_3_) spectrum of compound **5.**


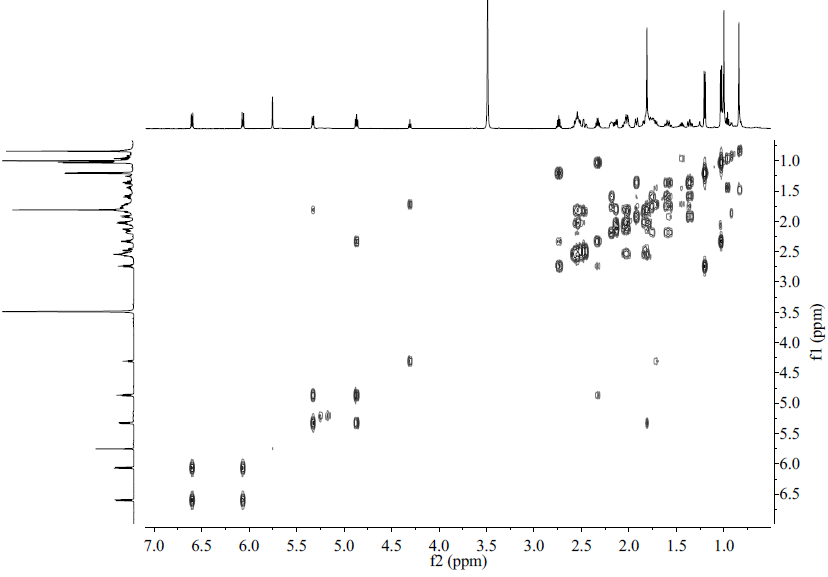


**Fig. S21** ^1^H-^1^H COSY (CDCl_3_) spectrum of compound **5.**


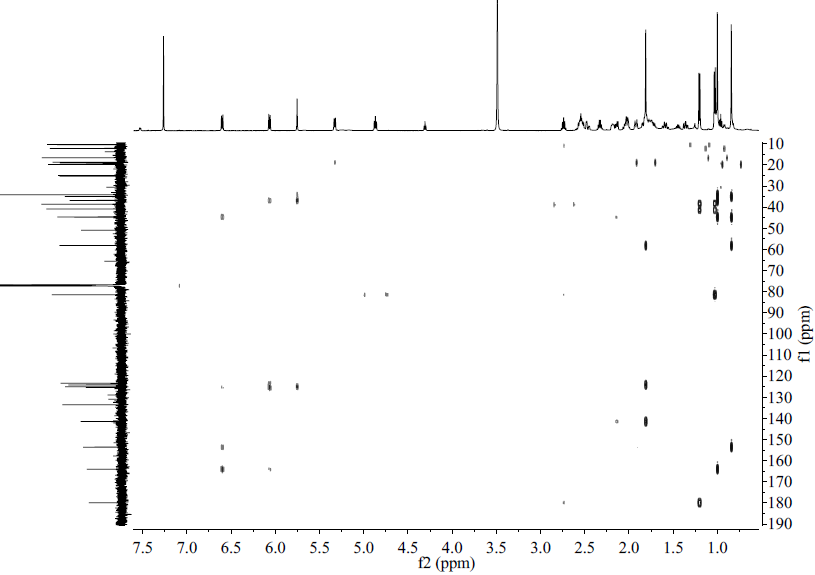


**Fig. S22** HMBC (CDCl_3_) spectrum of compound **5.**


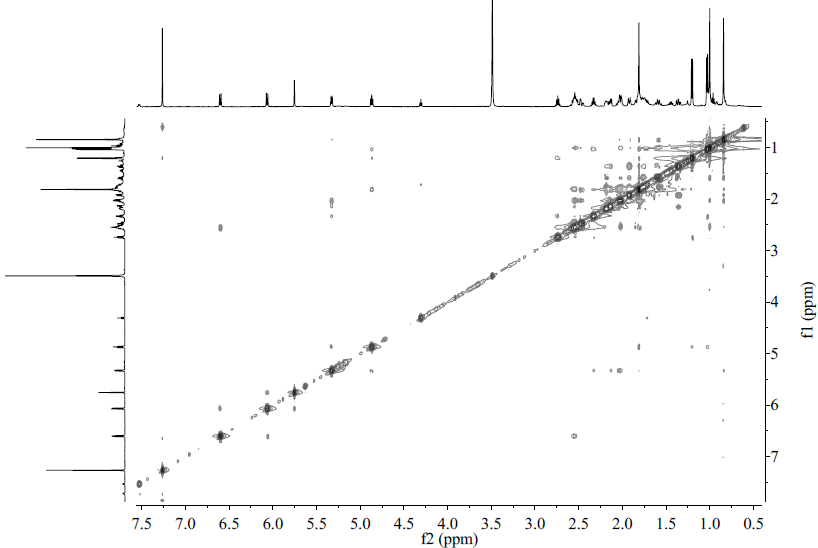

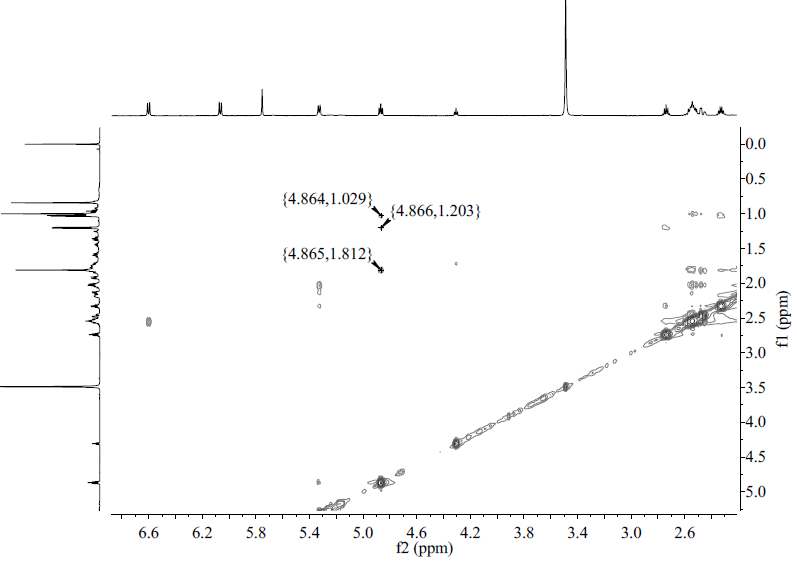


**Fig. S23** NOESY (CDCl_3_) spectrum of compound **5** and Local enlarged image.

**Fig. S24** HRESIMS spectrum of **5.**


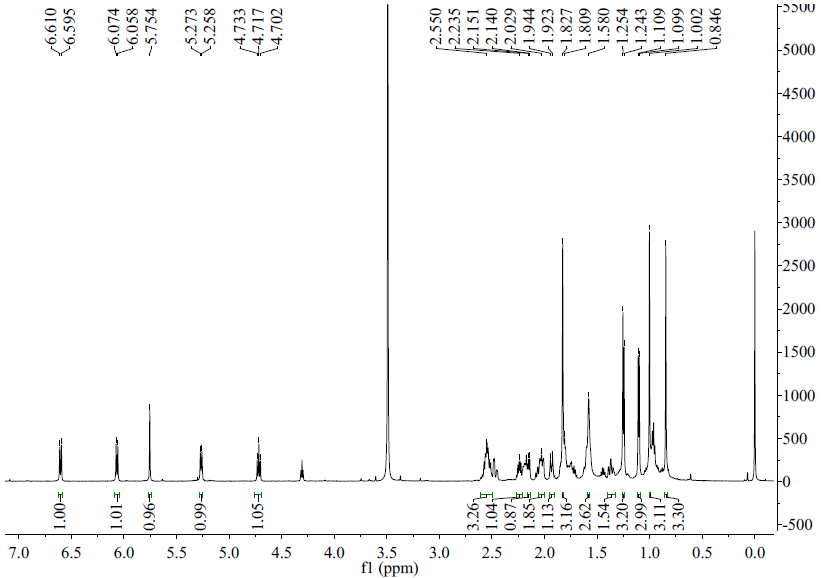


**Fig. S25** _1_H NMR (600 MHz, CDCl_3_) spectrum of compound **6.**


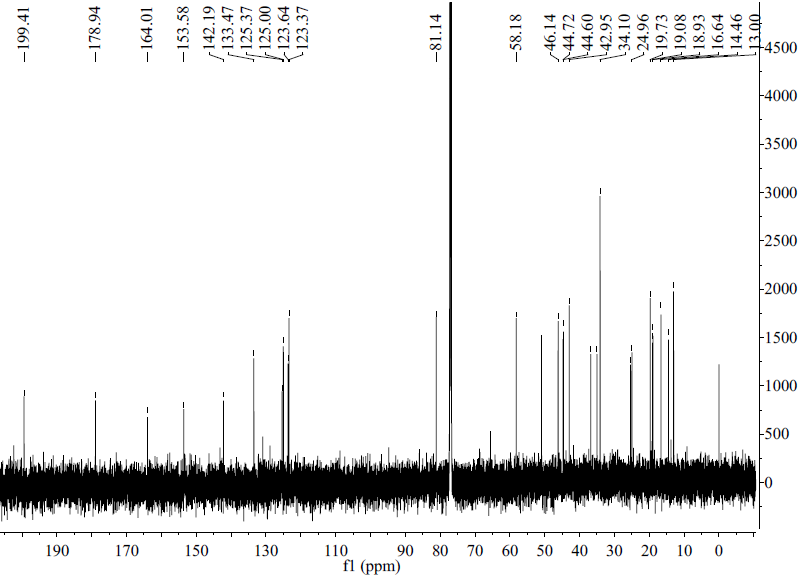


**Fig. S26** _13_C NMR (150 MHz, CDCl_3_) spectrum of compound **6.**


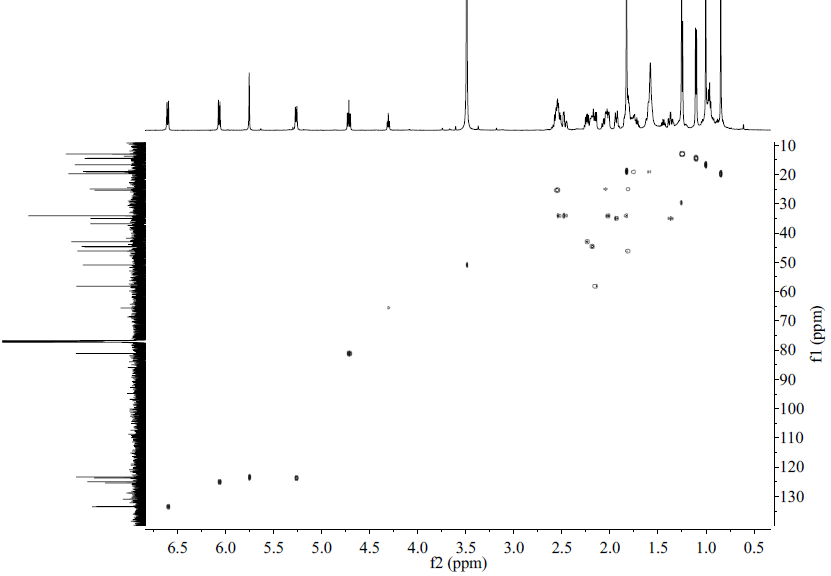


**Fig. S27** HSQC (CDCl_3_) spectrum of compound **6.**

\


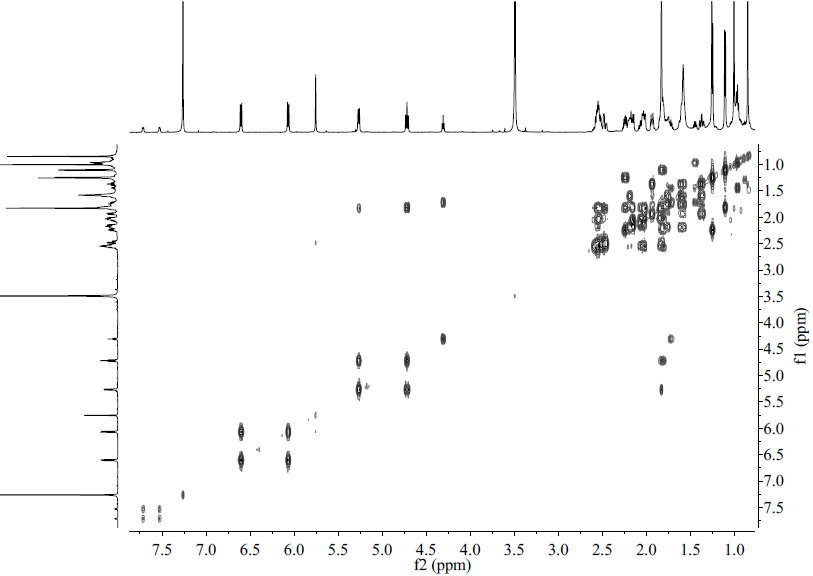


**Fig. S28** ^1^H-^1^H COSY (CDCl_3_) spectrum of compound **6.**


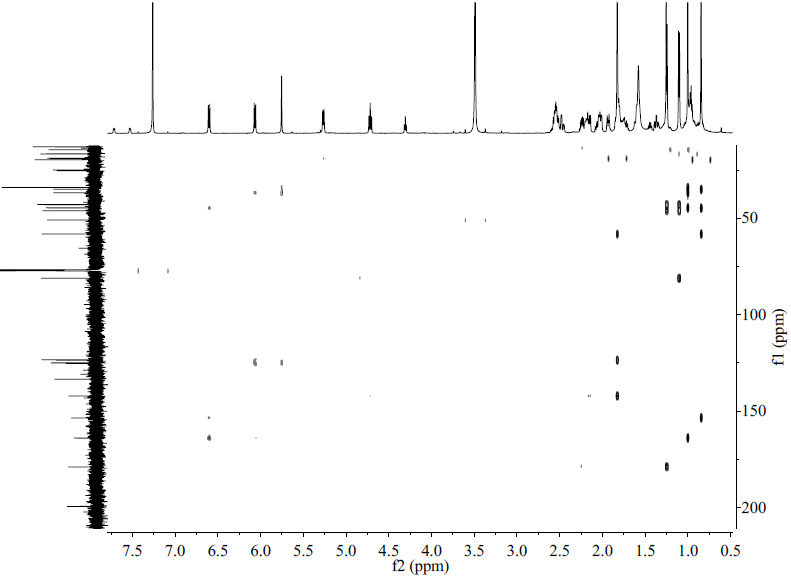


**Fig. S29** HMBC (CDCl_3_) spectrum of compound **6.**


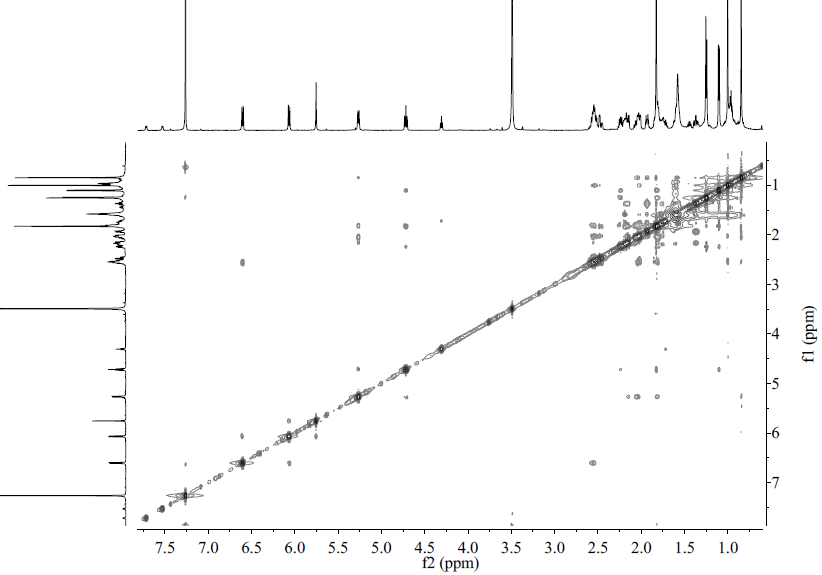

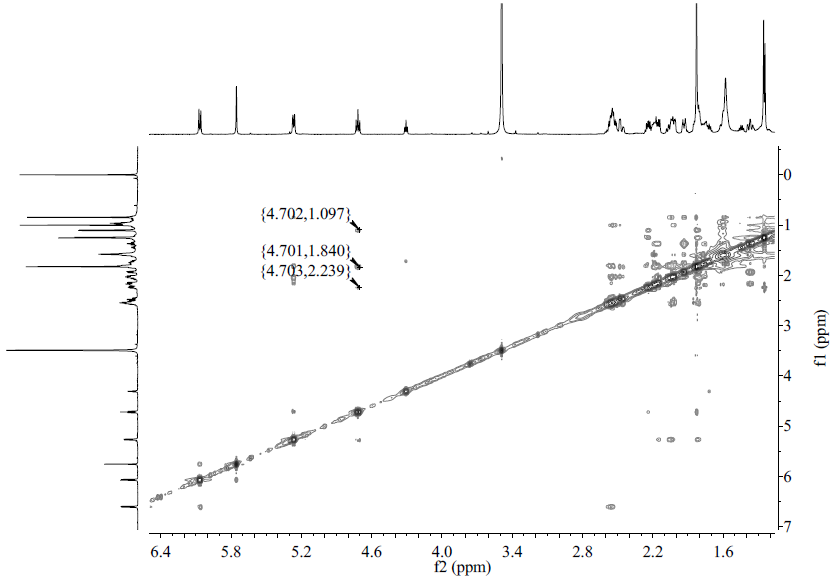


**Fig. S30** NOESY (CDCl_3_) spectrum of compound **6** and Local enlarged image.

**Fig. S31**. HRESIMS spectrum of **6.**


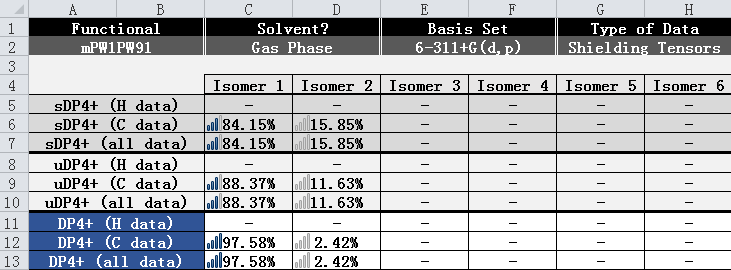

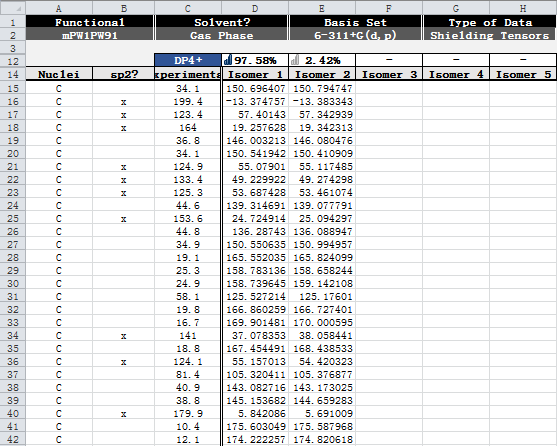


**Fig. S32** The data of DP4+ method of compound 5 (mPW1PW91/6-311+G(d,p)).


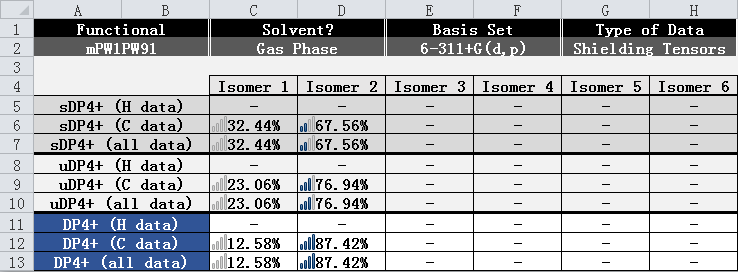

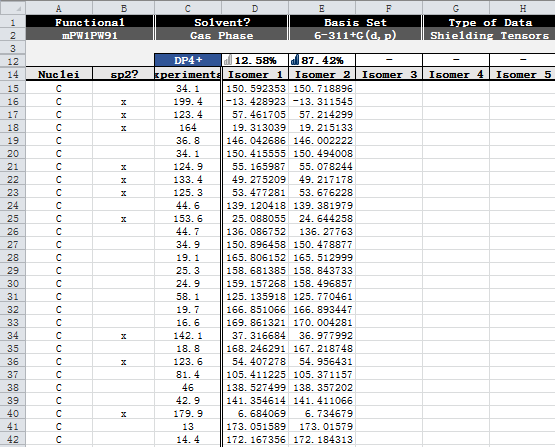


**Fig. S33** The data of DP4+ method of compound **6** (mPW1PW91/6-311+G(d,p)).

**Calculation details section**

Lowest energy conformers of compound **(3S,4S,7S,8S,9R,12S,26R)-1**

**C1** 12.27%


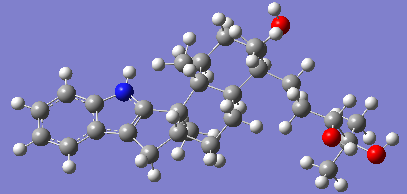


E=-1372.4910074 a.u.

Standard orientation:

----------------------------------------------------------------------------------------------------------

Center Atomic Atomic Coordinates (Angstroms)

Number Number Type X Y Z

-----------------------------------------------------------------------------------------------------------

1 6 0 -7.550115 -1.316073 -0.249088

2 6 0 -7.598252 -0.183043 -1.082074

3 6 0 -6.448964 0.542893 -1.373995

4 6 0 -5.245986 0.107715 -0.818001

5 6 0 -5.175027 -1.030345 0.043056

6 6 0 -6.354578 -1.742620 0.313560

7 7 0 -3.963378 0.624597 -0.952156

8 6 0 -3.111026 -0.124114 -0.163777

9 6 0 -3.800434 -1.140993 0.437747

10 6 0 -1.609581 -0.258586 -0.015095

11 6 0 -1.615596 -1.137731 1.285941

12 6 0 -2.863260 -2.056148 1.185970

13 6 0 -0.728010 1.027725 0.252901

14 6 0 0.723116 0.516013 0.621639

15 6 0 0.731969 -0.494152 1.794880

16 6 0 -0.232016 -1.683443 1.599519

17 6 0 -0.625253 1.901360 -1.020231

18 6 0 0.421565 3.015932 -0.914597

19 6 0 1.802963 2.459420 -0.573920

20 6 0 1.838224 1.621191 0.737963

21 6 0 5.081521 -0.615185 0.143820

22 6 0 3.687612 -0.068146 -0.162650

23 6 0 3.252942 0.976125 0.875954

24 1 0 -1.866499 -0.449750 2.098991

25 6 0 -1.398641 1.871526 1.369354

26 6 0 1.688349 2.536858 1.973256

27 6 0 5.711724 -1.519508 -0.953653

28 6 0 -1.148018 -1.077558 -1.255666

29 1 0 1.033098 -0.056335 -0.257525

30 1 0 2.102212 1.801893 -1.395550

31 8 0 6.921217 -2.076328 -0.366271

32 6 0 4.847486 -2.729871 -1.294372

33 8 0 2.795520 3.493611 -0.598861

34 8 0 4.995308 -1.336492 1.370009

35 6 0 6.082692 -0.724634 -2.205662

36 1 0 -8.465311 -1.862278 -0.043769

37 1 0 -8.547237 0.128740 -1.506232

38 1 0 -6.490399 1.414809 -2.020460

39 1 0 -6.332457 -2.615851 0.958394

40 1 0 -3.732903 1.471698 -1.443466

41 1 0 -2.662321 -2.982693 0.630563

42 1 0 -3.217843 -2.355787 2.177539

43 1 0 0.468309 0.010396 2.731071

44 1 0 1.745173 -0.874906 1.938344

45 1 0 -0.251556 -2.288300 2.513354

46 1 0 0.127325 -2.345594 0.803233

47 1 0 -0.365343 1.277676 -1.880317

48 1 0 -1.602294 2.345952 -1.248965

49 1 0 0.124332 3.763336 -0.169755

50 1 0 0.493451 3.551667 -1.866566

51 1 0 5.771437 0.236399 0.278407

52 1 0 3.692277 0.372566 -1.163997

53 1 0 2.997858 -0.916305 -0.184557

54 1 0 3.347226 0.532262 1.869203

55 1 0 3.973748 1.799038 0.847133

56 1 0 -1.126309 1.555428 2.375972

57 1 0 -2.487340 1.807347 1.296637

58 1 0 -1.142202 2.927785 1.292700

59 1 0 2.590446 3.140058 2.111839

60 1 0 0.840331 3.219014 1.918235

61 1 0 1.570729 1.949284 2.885861

62 1 0 -1.444426 -0.585437 -2.184154

63 1 0 -1.630154 -2.057155 -1.256503

64 1 0 -0.072001 -1.247561 -1.301554

65 1 0 7.624038 -1.414897 -0.406618

66 1 0 5.399052 -3.402651 -1.954164

67 1 0 3.929065 -2.427332 -1.802095

68 1 0 4.575688 -3.279603 -0.392047

69 1 0 2.524148 4.204361 -0.004770

70 1 0 5.815663 -1.844330 1.452281

71 1 0 6.717160 0.134755 -1.960404

72 1 0 6.628636 -1.362480 -2.905180

73 1 0 5.200044 -0.341370 -2.721713

-----------------------------------------------------------------------------------------------------------

**C2** 11.77%


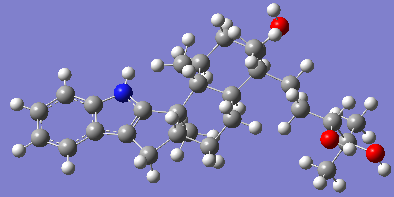


E= -1372.4909687 a.u.

Standard orientation:

----------------------------------------------------------------------------------------------------------

Center Atomic Atomic Coordinates (Angstroms)

Number Number Type X Y Z

----------------------------------------------------------------------------------------------------------

1 6 0 -7.546627 -1.316088 -0.251855

2 6 0 -7.594427 -0.182812 -1.084506

3 6 0 -6.445059 0.543461 -1.375417

4 6 0 -5.242390 0.108406 -0.818708

5 6 0 -5.171809 -1.029859 0.042128

6 6 0 -6.351360 -1.742521 0.311530

7 7 0 -3.959679 0.625253 -0.952334

8 6 0 -3.107822 -0.123189 -0.163134

9 6 0 -3.797437 -1.140312 0.437723

10 6 0 -1.606401 -0.257785 -0.014048

11 6 0 -1.612765 -1.137473 1.286599

12 6 0 -2.860558 -2.055686 1.186048

13 6 0 -0.724730 1.028274 0.254561

14 6 0 0.726452 0.516338 0.622956

15 6 0 0.735428 -0.494746 1.795383

16 6 0 -0.229263 -1.683492 1.599923

17 6 0 -0.622197 1.902603 -1.018100

18 6 0 0.424823 3.016901 -0.912075

19 6 0 1.806294 2.459924 -0.572038

20 6 0 1.841972 1.621409 0.739641

21 6 0 5.077083 -0.626447 0.156451

22 6 0 3.693153 -0.064340 -0.163437

23 6 0 3.256595 0.976257 0.877849

24 1 0 -1.863575 -0.449752 2.099900

25 6 0 -1.395291 1.871363 1.371547

26 6 0 1.691743 2.536565 1.975288

27 6 0 5.708856 -1.505527 -0.955648

28 6 0 -1.144895 -1.076434 -1.254876

29 1 0 1.036170 -0.055333 -0.256664

30 1 0 2.104905 1.802299 -1.393875

31 8 0 6.920438 -1.981303 -0.311905

32 6 0 4.832262 -2.700449 -1.336288

33 8 0 2.798868 3.493658 -0.597750

34 8 0 4.958883 -1.376506 1.363911

35 6 0 6.096417 -0.677743 -2.183301

36 1 0 -8.461853 -1.862555 -0.047327

37 1 0 -8.543178 0.128944 -1.509219

38 1 0 -6.486275 1.415531 -2.021691

39 1 0 -6.329518 -2.615901 0.956186

40 1 0 -3.729602 1.474331 -1.440463

41 1 0 -2.659576 -2.982180 0.630496

42 1 0 -3.215541 -2.355507 2.177441

43 1 0 0.472628 0.009230 2.732148

44 1 0 1.748519 -0.876156 1.937787

45 1 0 -0.248840 -2.288578 2.513655

46 1 0 0.129666 -2.345665 0.803402

47 1 0 -0.362489 1.279391 -1.878610

48 1 0 -1.599327 2.347341 -1.246393

49 1 0 0.127842 3.764078 -0.166928

50 1 0 0.496737 3.553042 -1.863816

51 1 0 5.777574 0.206387 0.319864

52 1 0 3.713248 0.383917 -1.160829

53 1 0 2.994484 -0.905060 -0.200187

54 1 0 3.349281 0.529159 1.869790

55 1 0 3.977804 1.798765 0.852576

56 1 0 -1.122855 1.554662 2.377964

57 1 0 -2.484051 1.807388 1.298911

58 1 0 -1.138678 2.927613 1.295512

59 1 0 2.594067 3.139214 2.114805

60 1 0 0.844108 3.219257 1.920217

61 1 0 1.573402 1.948666 2.887616

62 1 0 -1.441185 -0.583957 -2.183215

63 1 0 -1.627136 -2.056022 -1.256030

64 1 0 -0.068848 -1.246334 -1.300665

65 1 0 7.312829 -2.685832 -0.840933

66 1 0 5.375019 -3.374180 -2.008948

67 1 0 3.928827 -2.383167 -1.862568

68 1 0 4.535561 -3.262501 -0.449410

69 1 0 2.531411 4.201862 0.001166

70 1 0 5.812944 -1.807204 1.507448

71 1 0 6.737326 0.159544 -1.898132

72 1 0 6.650208 -1.295095 -2.898026

73 1 0 5.221932 -0.282786 -2.705814

------------------------------------------------------------------------------------------------------

**C3** 7.26%


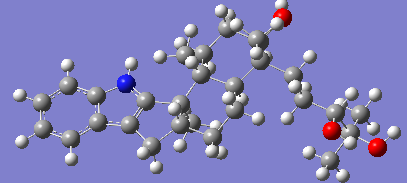


E= -1372.4905127 a.u.

Standard orientation:

---------------------------------------------------------------------

Center Atomic Atomic Coordinates (Angstroms)

Number Number Type X Y Z

---------------------------------------------------------------------

1 6 0 -7.546447 -1.323363 -0.265668

2 6 0 -7.596021 -0.184411 -1.090391

3 6 0 -6.447966 0.546524 -1.374930

4 6 0 -5.244946 0.110523 -0.819803

5 6 0 -5.172737 -1.033211 0.033594

6 6 0 -6.350849 -1.750656 0.296417

7 7 0 -3.963143 0.630958 -0.948876

8 6 0 -3.110793 -0.119766 -0.162060

9 6 0 -3.798773 -1.142539 0.430951

10 6 0 -1.609140 -0.252407 -0.012069

11 6 0 -1.615805 -1.138992 1.284042

12 6 0 -2.860935 -2.059910 1.175629

13 6 0 -0.729803 1.033045 0.266708

14 6 0 0.723223 0.518550 0.629852

15 6 0 0.731554 -0.493937 1.800418

16 6 0 -0.231832 -1.682899 1.598796

17 6 0 -0.631659 1.920419 -0.997790

18 6 0 0.418288 3.030689 -0.877269

19 6 0 1.796273 2.451525 -0.562184

20 6 0 1.840098 1.622941 0.746467

21 6 0 5.070232 -0.634672 0.154632

22 6 0 3.702579 -0.039945 -0.179965

23 6 0 3.255076 0.975444 0.881426

24 1 0 -1.870605 -0.455837 2.099860

25 6 0 -1.403494 1.865766 1.390077

26 6 0 1.697209 2.541362 1.979568

27 6 0 5.718290 -1.512699 -0.953610

28 6 0 -1.145826 -1.063278 -1.257416

29 1 0 1.028218 -0.053814 -0.250834

30 1 0 2.062176 1.776582 -1.389668

31 8 0 6.892222 -2.121947 -0.346738

32 6 0 4.838021 -2.687234 -1.370608

33 8 0 2.793044 3.480292 -0.478523

34 8 0 4.920379 -1.400966 1.347019

35 6 0 6.153624 -0.682350 -2.161202

36 1 0 -8.460565 -1.873513 -0.066097

37 1 0 -8.545036 0.128068 -1.514002

38 1 0 -6.490515 1.422963 -2.015214

39 1 0 -6.327683 -2.628315 0.935181

40 1 0 -3.736056 1.487688 -1.424868

41 1 0 -2.656580 -2.982672 0.615104

42 1 0 -3.217188 -2.366363 2.164520

43 1 0 0.468893 0.008990 2.737579

44 1 0 1.743942 -0.877253 1.942084

45 1 0 -0.252776 -2.291269 2.510303

46 1 0 0.130249 -2.341850 0.800941

47 1 0 -0.377646 1.306334 -1.866693

48 1 0 -1.608624 2.370394 -1.215373

49 1 0 0.145659 3.767489 -0.117405

50 1 0 0.470525 3.580240 -1.827050

51 1 0 5.775905 0.192478 0.348499

52 1 0 3.753729 0.437730 -1.163093

53 1 0 2.993650 -0.868628 -0.259215

54 1 0 3.333186 0.503042 1.862304

55 1 0 3.976291 1.797675 0.889110

56 1 0 -1.122941 1.548837 2.394024

57 1 0 -2.491919 1.790908 1.321771

58 1 0 -1.157281 2.924467 1.316328

59 1 0 2.557028 3.209696 2.044619

60 1 0 0.806559 3.166117 1.961592

61 1 0 1.667825 1.951974 2.898961

62 1 0 -1.440225 -0.564380 -2.183051

63 1 0 -1.628950 -2.042345 -1.266185

64 1 0 -0.069997 -1.234279 -1.302435

65 1 0 7.609125 -1.474712 -0.328893

66 1 0 5.395492 -3.347197 -2.038357

67 1 0 3.944172 -2.342640 -1.894957

68 1 0 4.523278 -3.264554 -0.500056

69 1 0 2.748046 4.023148 -1.274396

70 1 0 5.725123 -1.931167 1.440893

71 1 0 6.795527 0.153918 -1.861257

72 1 0 6.713247 -1.307245 -2.861636

73 1 0 5.298932 -0.263557 -2.696540

---------------------------------------------------------------------

**C4** 6.86%


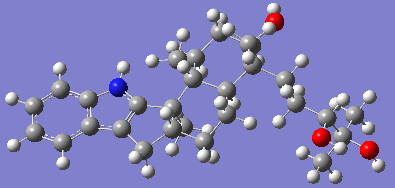


E= -1372.4904587 a.u.

Standard orientation:

---------------------------------------------------------------------

Center Atomic Atomic Coordinates (Angstroms)

Number Number Type X Y Z

---------------------------------------------------------------------

1 6 0 -7.549603 -1.310219 -0.277266

2 6 0 -7.595938 -0.166797 -1.095985

3 6 0 -6.445858 0.562578 -1.376394

4 6 0 -5.244100 0.120496 -0.823323

5 6 0 -5.175148 -1.028075 0.023875

6 6 0 -6.355248 -1.743700 0.282723

7 7 0 -3.960938 0.638410 -0.948926

8 6 0 -3.110820 -0.118703 -0.165825

9 6 0 -3.801691 -1.143028 0.421167

10 6 0 -1.609539 -0.255151 -0.014818

11 6 0 -1.619528 -1.149306 1.276149

12 6 0 -2.866715 -2.066571 1.161651

13 6 0 -0.728036 1.026778 0.272674

14 6 0 0.723845 0.507768 0.634810

15 6 0 0.729565 -0.513740 1.797840

16 6 0 -0.237210 -1.698447 1.588411

17 6 0 -0.626459 1.921591 -0.986500

18 6 0 0.424848 3.029531 -0.857619

19 6 0 1.801451 2.446449 -0.543263

20 6 0 1.841650 1.610434 0.760766

21 6 0 5.052781 -0.671284 0.183351

22 6 0 3.716163 -0.028979 -0.183607

23 6 0 3.255794 0.961699 0.895601

24 1 0 -1.873154 -0.470202 2.095708

25 6 0 -1.402111 1.854088 1.399866

26 6 0 1.696381 2.521804 1.999003

27 6 0 5.740730 -1.470614 -0.955262

28 6 0 -1.146207 -1.059854 -1.264181

29 1 0 1.029304 -0.058856 -0.249409

30 1 0 2.067762 1.775347 -1.373803

31 8 0 6.879834 -2.055183 -0.270338

32 6 0 4.855269 -2.585926 -1.513983

33 8 0 2.799467 3.472848 -0.451497

34 8 0 4.816974 -1.521382 1.304186

35 6 0 6.255949 -0.548575 -2.063336

36 1 0 -8.465269 -1.858852 -0.080586

37 1 0 -8.544046 0.150500 -1.518051

38 1 0 -6.485945 1.442559 -2.011962

39 1 0 -6.334578 -2.624642 0.917060

40 1 0 -3.731358 1.497150 -1.420102

41 1 0 -2.664090 -2.986843 0.596352

42 1 0 -3.224548 -2.377479 2.148602

43 1 0 0.469199 -0.016994 2.738988

44 1 0 1.740679 -0.901812 1.934992

45 1 0 -0.260212 -2.312211 2.496280

46 1 0 0.123607 -2.353687 0.786934

47 1 0 -0.371796 1.312159 -1.858517

48 1 0 -1.602531 2.374053 -1.203129

49 1 0 0.151978 3.762330 -0.093992

50 1 0 0.479731 3.584574 -1.804039

51 1 0 5.765324 0.115103 0.475610

52 1 0 3.815971 0.481758 -1.145614

53 1 0 2.989160 -0.834693 -0.319768

54 1 0 3.324192 0.468239 1.866784

55 1 0 3.976407 1.783604 0.927556

56 1 0 -1.122466 1.531775 2.402361

57 1 0 -2.490558 1.780578 1.330364

58 1 0 -1.154979 2.912914 1.331654

59 1 0 2.554417 3.192043 2.067319

60 1 0 0.804107 3.144272 1.984927

61 1 0 1.669045 1.927252 2.915182

62 1 0 -1.437955 -0.554835 -2.187343

63 1 0 -1.631570 -2.037766 -1.279303

64 1 0 -0.070668 -1.232934 -1.308301

65 1 0 7.280778 -2.731550 -0.828826

66 1 0 5.424926 -3.219351 -2.203587

67 1 0 4.009172 -2.182109 -2.075316

68 1 0 4.468449 -3.211968 -0.708523

69 1 0 2.764758 4.014021 -1.249020

70 1 0 5.645087 -1.990845 1.475225

71 1 0 6.897063 0.232091 -1.647337

72 1 0 6.848133 -1.117685 -2.787046

73 1 0 5.441559 -0.071617 -2.613793

---------------------------------------------------------------------

**C5** 5.34%


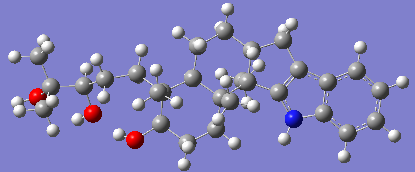


E=- 1372.490222 a.u.

Standard orientation:

---------------------------------------------------------------------

Center Atomic Atomic Coordinates (Angstroms)

Number Number Type X Y Z

---------------------------------------------------------------------

1 6 0 8.089626 0.040261 -0.166580

2 6 0 7.882913 -1.350132 -0.231514

3 6 0 6.599738 -1.884609 -0.264066

4 6 0 5.524135 -0.996968 -0.235585

5 6 0 5.709247 0.417711 -0.154964

6 6 0 7.018958 0.923471 -0.126916

7 7 0 4.158742 -1.249252 -0.271849

8 6 0 3.492757 -0.043944 -0.166963

9 6 0 4.393002 0.985311 -0.111893

10 6 0 2.065960 0.428649 -0.364876

11 6 0 2.253141 1.866760 0.235733

12 6 0 3.687751 2.318214 -0.147230

13 6 0 0.882638 -0.288307 0.404125

14 6 0 -0.414416 0.599141 0.173274

15 6 0 -0.191175 2.059980 0.644773

16 6 0 1.032754 2.738725 -0.006774

17 6 0 0.633151 -1.704757 -0.162987

18 6 0 -0.658076 -2.343607 0.352243

19 6 0 -1.865142 -1.475158 0.027461

20 6 0 -1.792231 -0.032295 0.630927

21 6 0 -5.346838 0.252948 -0.715735

22 6 0 -4.399882 0.556440 0.441228

23 6 0 -2.938294 0.809294 -0.025234

24 1 0 2.312975 1.720552 1.318378

25 6 0 1.281441 -0.447650 1.896056

26 6 0 -1.974454 -0.044289 2.159276

27 6 0 -6.854273 0.206163 -0.358080

28 6 0 1.865829 0.472835 -1.907989

29 1 0 -0.523475 0.654073 -0.915750

30 1 0 -1.891480 -1.348720 -1.065594

31 8 0 -7.441937 -0.262477 -1.601450

32 6 0 -7.172763 -0.785692 0.762274

33 8 0 -3.031060 -2.193993 0.425334

34 8 0 -4.945116 -1.007658 -1.271261

35 6 0 -7.401460 1.601459 -0.048210

36 1 0 9.104381 0.424964 -0.145327

37 1 0 8.738484 -2.017220 -0.259046

38 1 0 6.445189 -2.958443 -0.317037

39 1 0 7.192448 1.993925 -0.072175

40 1 0 3.741406 -2.164481 -0.259578

41 1 0 3.734706 2.782852 -1.141952

42 1 0 4.076430 3.055993 0.562484

43 1 0 -0.066932 2.090745 1.732504

44 1 0 -1.073642 2.665793 0.433303

45 1 0 1.174073 3.733249 0.432797

46 1 0 0.858406 2.897193 -1.077530

47 1 0 0.578832 -1.667972 -1.255305

48 1 0 1.483426 -2.354756 0.081134

49 1 0 -0.620638 -2.537767 1.428373

50 1 0 -0.810095 -3.317965 -0.122066

51 1 0 -5.237546 1.019751 -1.496086

52 1 0 -4.768115 1.437607 0.973172

53 1 0 -4.451243 -0.270362 1.147867

54 1 0 -2.879315 0.656306 -1.108118

55 1 0 -2.733958 1.868259 0.130758

56 1 0 1.095146 0.439272 2.500636

57 1 0 2.347889 -0.673014 1.981149

58 1 0 0.748229 -1.270009 2.371321

59 1 0 -2.866664 -0.604517 2.439266

60 1 0 -1.139309 -0.505262 2.681102

61 1 0 -2.081050 0.971980 2.550740

62 1 0 2.053485 -0.505450 -2.354728

63 1 0 2.582403 1.162417 -2.358674

64 1 0 0.871037 0.791066 -2.219971

65 1 0 -8.361504 -0.512350 -1.453000

66 1 0 -8.257013 -0.890621 0.880651

67 1 0 -6.776988 -0.448040 1.722426

68 1 0 -6.754702 -1.770209 0.546746

69 1 0 -3.754998 -1.966345 -0.180388

70 1 0 -5.625617 -1.270800 -1.906785

71 1 0 -7.184755 2.292333 -0.866223

72 1 0 -8.487990 1.563092 0.077893

73 1 0 -6.982425 2.010943 0.873950

---------------------------------------------------------------------

**C6** 4.92%


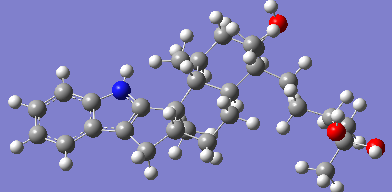


E= -1372.4901462 a.u.

Standard orientation:

---------------------------------------------------------------------

Center Atomic Atomic Coordinates (Angstroms)

Number Number Type X Y Z

---------------------------------------------------------------------

1 6 0 -7.548101 -1.306925 -0.230121

2 6 0 -7.588441 -0.198959 -1.096611

3 6 0 -6.435603 0.514622 -1.404109

4 6 0 -5.236796 0.092344 -0.829367

5 6 0 -5.173771 -1.019869 0.065127

6 6 0 -6.356807 -1.720354 0.350816

7 7 0 -3.951929 0.601624 -0.971966

8 6 0 -3.105819 -0.126301 -0.157892

9 6 0 -3.801419 -1.122973 0.469735

10 6 0 -1.605588 -0.261277 0.001986

11 6 0 -1.621599 -1.101617 1.328470

12 6 0 -2.870823 -2.019724 1.247924

13 6 0 -0.721416 1.029912 0.237503

14 6 0 0.725485 0.523549 0.628443

15 6 0 0.721793 -0.446300 1.834806

16 6 0 -0.241379 -1.640860 1.668133

17 6 0 -0.608450 1.865884 -1.059377

18 6 0 0.437799 2.983155 -0.978055

19 6 0 1.817069 2.437916 -0.613909

20 6 0 1.843350 1.627440 0.716554

21 6 0 5.121217 -0.559422 0.062050

22 6 0 3.660785 -0.133047 -0.119394

23 6 0 3.257859 0.978625 0.860828

24 1 0 -1.876559 -0.389659 2.119374

25 6 0 -1.394629 1.907323 1.326183

26 6 0 1.700919 2.572483 1.929179

27 6 0 5.668160 -1.576160 -0.971652

28 6 0 -1.140090 -1.116725 -1.211785

29 1 0 1.038227 -0.078266 -0.229665

30 1 0 2.126093 1.765305 -1.419292

31 8 0 7.020073 -1.874935 -0.591579

32 6 0 4.851612 -2.876037 -1.002045

33 8 0 2.807544 3.473196 -0.646237

34 8 0 5.318366 -1.181281 1.352725

35 6 0 5.769354 -0.957439 -2.364250

36 1 0 -8.466040 -1.843988 -0.013441

37 1 0 -8.534295 0.102934 -1.534616

38 1 0 -6.471194 1.367182 -2.076186

39 1 0 -6.340625 -2.574455 1.020913

40 1 0 -3.716723 1.432011 -1.488844

41 1 0 -2.669259 -2.961545 0.719230

42 1 0 -3.231088 -2.290853 2.245599

43 1 0 0.448924 0.088907 2.750943

44 1 0 1.733350 -0.823537 2.001672

45 1 0 -0.268880 -2.218383 2.599244

46 1 0 0.123345 -2.325667 0.893956

47 1 0 -0.342740 1.217624 -1.899203

48 1 0 -1.583104 2.304579 -1.307818

49 1 0 0.135431 3.750592 -0.255775

50 1 0 0.515325 3.492446 -1.943897

51 1 0 5.770674 0.323618 0.000222

52 1 0 3.530060 0.209053 -1.150273

53 1 0 3.029640 -1.016413 0.009063

54 1 0 3.353576 0.601624 1.884556

55 1 0 3.979367 1.797129 0.768470

56 1 0 -1.129670 1.618374 2.342889

57 1 0 -2.482970 1.844356 1.248882

58 1 0 -1.134490 2.960400 1.222040

59 1 0 2.602788 3.180470 2.045452

60 1 0 0.852121 3.252218 1.860382

61 1 0 1.586470 2.008660 2.857272

62 1 0 -1.429585 -0.650036 -2.155427

63 1 0 -1.626380 -2.093915 -1.187588

64 1 0 -0.064565 -1.292475 -1.247691

65 1 0 7.005684 -2.061699 0.357845

66 1 0 5.356253 -3.599774 -1.645598

67 1 0 3.842705 -2.719048 -1.393887

68 1 0 4.765598 -3.308534 -0.002740

69 1 0 2.502367 4.219117 -0.115086

70 1 0 5.396715 -0.500386 2.028530

71 1 0 6.331958 -0.021005 -2.335569

72 1 0 6.301836 -1.646075 -3.023875

73 1 0 4.787276 -0.763689 -2.800597

---------------------------------------------------------------------

**C7** 4.69%


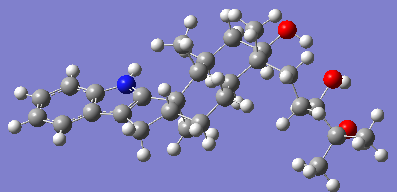


E= -1372.4900999 a.u.

Standard orientation:

---------------------------------------------------------------------

Center Atomic Atomic Coordinates (Angstroms)

Number Number Type X Y Z

---------------------------------------------------------------------

1 6 0 -7.541630 -0.993258 -0.467522

2 6 0 -7.385707 -0.170129 -1.598042

3 6 0 -6.145952 0.362782 -1.932914

4 6 0 -5.060641 0.050298 -1.114215

5 6 0 -5.197884 -0.770421 0.047594

6 6 0 -6.463803 -1.294911 0.354235

7 7 0 -3.726689 0.423330 -1.220570

8 6 0 -3.044883 -0.095882 -0.137529

9 6 0 -3.893417 -0.835510 0.640248

10 6 0 -1.588372 -0.277068 0.237450

11 6 0 -1.821006 -0.718074 1.727322

12 6 0 -3.138323 -1.538443 1.740908

13 6 0 -0.611572 0.967166 0.233120

14 6 0 0.737756 0.483586 0.910407

15 6 0 0.516798 -0.110617 2.323264

16 6 0 -0.541900 -1.233536 2.367003

17 6 0 -0.305877 1.432410 -1.212232

18 6 0 0.850332 2.434894 -1.307080

19 6 0 2.116445 1.859854 -0.685910

20 6 0 1.943632 1.487684 0.819246

21 6 0 4.411594 -0.552087 -0.551709

22 6 0 3.788291 -0.516419 0.849445

23 6 0 3.261610 0.843038 1.358723

24 1 0 -2.082612 0.199908 2.262000

25 6 0 -1.300218 2.145239 0.975175

26 6 0 1.774749 2.766524 1.668258

27 6 0 5.328174 -1.774170 -0.825321

28 6 0 -1.090481 -1.463618 -0.638712

29 1 0 1.064729 -0.356998 0.289281

30 1 0 2.331318 0.929462 -1.228021

31 8 0 5.783116 -1.498519 -2.176929

32 6 0 6.541049 -1.849037 0.106121

33 8 0 3.183454 2.774984 -0.912378

34 8 0 5.154718 0.655622 -0.769022

35 6 0 4.532056 -3.080370 -0.817503

36 1 0 -8.522708 -1.395708 -0.235881

37 1 0 -8.246232 0.050748 -2.221294

38 1 0 -6.029953 0.994666 -2.808724

39 1 0 -6.598941 -1.927572 1.226275

40 1 0 -3.360624 1.065700 -1.902893

41 1 0 -2.976444 -2.601723 1.515171

42 1 0 -3.623647 -1.497007 2.721618

43 1 0 0.216110 0.676846 3.023141

44 1 0 1.462360 -0.500571 2.707454

45 1 0 -0.720267 -1.526258 3.408387

46 1 0 -0.173993 -2.130713 1.855107

47 1 0 -0.056266 0.569664 -1.837183

48 1 0 -1.207232 1.878440 -1.652289

49 1 0 0.603646 3.389673 -0.835452

50 1 0 1.059660 2.663387 -2.356538

51 1 0 3.622473 -0.607846 -1.311550

52 1 0 3.006372 -1.280724 0.892584

53 1 0 4.549134 -0.826206 1.572854

54 1 0 3.153139 0.741062 2.442379

55 1 0 4.060732 1.577247 1.236039

56 1 0 -1.131408 2.145065 2.051741

57 1 0 -2.381694 2.113422 0.819182

58 1 0 -0.959894 3.111361 0.604177

59 1 0 2.676715 3.376638 1.594054

60 1 0 0.942868 3.393022 1.356699

61 1 0 1.624790 2.522865 2.723514

62 1 0 -1.227275 -1.251362 -1.700868

63 1 0 -1.676624 -2.359323 -0.423223

64 1 0 -0.041449 -1.719250 -0.487311

65 1 0 6.502515 -2.097975 -2.408238

66 1 0 7.234522 -2.622922 -0.241048

67 1 0 6.257210 -2.115173 1.126319

68 1 0 7.073820 -0.897361 0.134686

69 1 0 4.011635 2.270272 -0.868177

70 1 0 5.648846 0.521493 -1.591293

71 1 0 3.686564 -3.023066 -1.506960

72 1 0 5.165396 -3.914977 -1.133997

73 1 0 4.154307 -3.317784 0.179901

---------------------------------------------------------------------

**C8** 4.12%


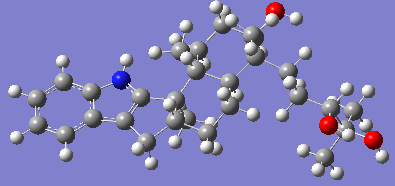


E= -1372.4899783 a.u.

Standard orientation:

---------------------------------------------------------------------

Center Atomic Atomic Coordinates (Angstroms)

Number Number Type X Y Z

---------------------------------------------------------------------

1 6 0 -7.545141 -1.318002 -0.259405

2 6 0 -7.591106 -0.185424 -1.093109

3 6 0 -6.441129 0.540745 -1.381791

4 6 0 -5.239742 0.106360 -0.821815

5 6 0 -5.171056 -1.031113 0.040379

6 6 0 -6.351216 -1.743698 0.307409

7 7 0 -3.956926 0.623393 -0.952896

8 6 0 -3.107124 -0.123681 -0.160618

9 6 0 -3.797691 -1.140488 0.439764

10 6 0 -1.606145 -0.257028 -0.007397

11 6 0 -1.615652 -1.133481 1.295047

12 6 0 -2.862147 -2.053477 1.192908

13 6 0 -0.726795 1.030562 0.260778

14 6 0 0.725464 0.518924 0.628483

15 6 0 0.732267 -0.487045 1.805656

16 6 0 -0.232408 -1.676870 1.613781

17 6 0 -0.626954 1.907002 -1.010605

18 6 0 0.418773 3.020416 -0.904366

19 6 0 1.795031 2.457467 -0.578418

20 6 0 1.839115 1.628029 0.740120

21 6 0 5.087529 -0.610709 0.142895

22 6 0 3.697699 -0.057088 -0.166859

23 6 0 3.250229 0.970811 0.883329

24 1 0 -1.869497 -0.443906 2.105690

25 6 0 -1.399894 1.872301 1.377714

26 6 0 1.694016 2.549880 1.968931

27 6 0 5.702326 -1.514548 -0.959423

28 6 0 -1.142542 -1.078349 -1.245834

29 1 0 1.032040 -0.056893 -0.249709

30 1 0 2.075011 1.784010 -1.402127

31 8 0 6.927907 -1.965901 -0.326147

32 6 0 4.825135 -2.722928 -1.292817

33 8 0 2.692301 3.574936 -0.579205

34 8 0 4.991106 -1.330406 1.368472

35 6 0 6.062009 -0.716591 -2.215114

36 1 0 -8.460807 -1.864380 -0.056567

37 1 0 -8.538898 0.125969 -1.520229

38 1 0 -6.480778 1.412472 -2.028601

39 1 0 -6.330948 -2.616414 0.953034

40 1 0 -3.725975 1.473732 -1.438514

41 1 0 -2.658515 -2.981102 0.640093

42 1 0 -3.219916 -2.351367 2.183894

43 1 0 0.469351 0.022976 2.738801

44 1 0 1.743667 -0.871734 1.952838

45 1 0 -0.253259 -2.277783 2.530264

46 1 0 0.128338 -2.342482 0.820775

47 1 0 -0.374022 1.284519 -1.873920

48 1 0 -1.604582 2.353843 -1.231957

49 1 0 0.143823 3.765898 -0.153756

50 1 0 0.487791 3.561550 -1.853019

51 1 0 5.791105 0.226350 0.274547

52 1 0 3.706881 0.389279 -1.165868

53 1 0 3.001662 -0.899293 -0.201817

54 1 0 3.326623 0.507031 1.867957

55 1 0 3.995263 1.778005 0.913398

56 1 0 -1.120554 1.563519 2.384712

57 1 0 -2.488262 1.797502 1.309512

58 1 0 -1.153146 2.930018 1.294134

59 1 0 2.556728 3.214831 2.045205

60 1 0 0.810805 3.183628 1.936130

61 1 0 1.650199 1.966618 2.891666

62 1 0 -1.435639 -0.586435 -2.175389

63 1 0 -1.626063 -2.057328 -1.246882

64 1 0 -0.066567 -1.249732 -1.289069

65 1 0 7.308885 -2.690858 -0.835538

66 1 0 5.360269 -3.412372 -1.955429

67 1 0 3.912033 -2.424688 -1.813446

68 1 0 4.545453 -3.261904 -0.386416

69 1 0 3.598962 3.261932 -0.483192

70 1 0 5.846533 -1.760356 1.506667

71 1 0 6.704358 0.130846 -1.964189

72 1 0 6.606875 -1.348466 -2.923574

73 1 0 5.175636 -0.341670 -2.732542

---------------------------------------------------------------------

Lowest energy conformers of compound **(3R,4R,7R,8R,9S,12R,26R)-1**

**C1** 54.98%


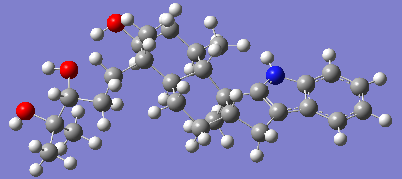


E= -1372.4930986 a.u.

Standard orientation:

---------------------------------------------------------------------

Center Atomic Atomic Coordinates (Angstroms)

Number Number Type X Y Z

---------------------------------------------------------------------

1 6 0 7.646706 -0.914416 -0.560722

2 6 0 7.512137 0.112321 -1.513501

3 6 0 6.277006 0.697366 -1.769288

4 6 0 5.174596 0.229525 -1.054055

5 6 0 5.289789 -0.801109 -0.070666

6 6 0 6.551617 -1.372368 0.159641

7 7 0 3.841336 0.614909 -1.116987

8 6 0 3.137956 -0.105501 -0.172158

9 6 0 3.972527 -0.981398 0.466936

10 6 0 1.674674 -0.347899 0.138502

11 6 0 1.883811 -1.061075 1.522877

12 6 0 3.197883 -1.878164 1.399785

13 6 0 0.700067 0.880191 0.351184

14 6 0 -0.658536 0.287082 0.913386

15 6 0 -0.457141 -0.558586 2.194702

16 6 0 0.593912 -1.678056 2.038121

17 6 0 0.410286 1.600997 -0.988748

18 6 0 -0.740645 2.611358 -0.912421

19 6 0 -2.016587 1.941338 -0.419479

20 6 0 -1.861259 1.296304 0.993556

21 6 0 -5.114594 -0.354742 -0.065153

22 6 0 -3.677820 -0.571305 0.402662

23 6 0 -3.193924 0.561860 1.340558

24 1 0 2.143213 -0.261829 2.224087

25 6 0 1.383199 1.900759 1.301368

26 6 0 -1.710590 2.383976 2.075633

27 6 0 -5.743160 -1.509949 -0.884816

28 6 0 1.188457 -1.347853 -0.950429

29 1 0 -0.978820 -0.425258 0.146388

30 1 0 -2.239306 1.129872 -1.126422

31 8 0 -7.018923 -0.937279 -1.275421

32 6 0 -4.936725 -1.862510 -2.136475

33 8 0 -3.080583 2.886252 -0.479847

34 8 0 -5.130105 0.849346 -0.845789

35 6 0 -5.998154 -2.742460 -0.013690

36 1 0 8.624885 -1.351480 -0.386675

37 1 0 8.385992 0.452865 -2.059497

38 1 0 6.177843 1.487948 -2.507478

39 1 0 6.670425 -2.161849 0.895553

40 1 0 3.485941 1.373553 -1.674032

41 1 0 3.666740 -2.028549 2.377824

42 1 0 3.036614 -2.877190 0.971257

43 1 0 -1.409089 -1.007685 2.489453

44 1 0 -0.160126 0.082752 3.031515

45 1 0 0.226068 -2.458586 1.361485

46 1 0 0.757229 -2.164171 3.007190

47 1 0 1.318406 2.111579 -1.334471

48 1 0 0.160015 0.866280 -1.760085

49 1 0 -0.936020 3.027764 -1.905319

50 1 0 -0.495583 3.464164 -0.272512

51 1 0 -5.764463 -0.208006 0.809489

52 1 0 -3.609193 -1.536369 0.911806

53 1 0 -3.045326 -0.651858 -0.484437

54 1 0 -3.964433 1.335024 1.379310

55 1 0 -3.130159 0.181841 2.362974

56 1 0 1.214771 1.697624 2.358789

57 1 0 1.035419 2.916556 1.117278

58 1 0 2.464814 1.905934 1.143305

59 1 0 -1.638944 1.939742 3.072473

60 1 0 -0.838651 3.017802 1.937324

61 1 0 -2.585865 3.036078 2.063797

62 1 0 1.353500 -0.947888 -1.952757

63 1 0 0.133668 -1.612221 -0.872142

64 1 0 1.760576 -2.275694 -0.887367

65 1 0 -7.440567 -1.498952 -1.936458

66 1 0 -5.491357 -2.573276 -2.758916

67 1 0 -3.986154 -2.336698 -1.881445

68 1 0 -4.730499 -0.971945 -2.731968

69 1 0 -3.904869 2.391769 -0.612798

70 1 0 -6.018785 0.929823 -1.220008

71 1 0 -6.593420 -2.479924 0.863825

72 1 0 -6.552000 -3.498294 -0.579446

73 1 0 -5.067891 -3.206280 0.322609

---------------------------------------------------------------------

**C2** 41.65%


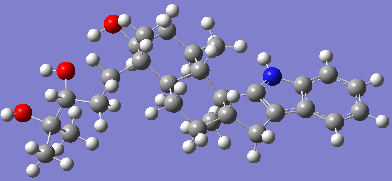


E= -1372.4928366 a.u.

Standard orientation:

---------------------------------------------------------------------

Center Atomic Atomic Coordinates (Angstroms)

Number Number Type X Y Z

---------------------------------------------------------------------

1 6 0 7.648323 -0.913598 -0.560398

2 6 0 7.512750 0.110577 -1.515801

3 6 0 6.277200 0.694248 -1.772603

4 6 0 5.175363 0.227659 -1.055651

5 6 0 5.291546 -0.800443 -0.069751

6 6 0 6.553807 -1.370356 0.161555

7 7 0 3.841888 0.612210 -1.118907

8 6 0 3.139219 -0.106498 -0.172296

9 6 0 3.974582 -0.980299 0.468650

10 6 0 1.676221 -0.348876 0.139670

11 6 0 1.886431 -1.058678 1.525620

12 6 0 3.200881 -1.875304 1.403928

13 6 0 0.701323 0.879407 0.350158

14 6 0 -0.656581 0.287137 0.914729

15 6 0 -0.454177 -0.555907 2.197645

16 6 0 0.597151 -1.675331 2.042788

17 6 0 0.409729 1.596618 -0.991257

18 6 0 -0.740823 2.607504 -0.915923

19 6 0 -2.016613 1.939662 -0.419466

20 6 0 -1.859140 1.296479 0.994229

21 6 0 -5.111431 -0.360145 -0.063417

22 6 0 -3.674580 -0.573698 0.408285

23 6 0 -3.191364 0.562386 1.343554

24 1 0 2.145607 -0.257669 2.224919

25 6 0 1.384833 1.902595 1.297271

26 6 0 -1.707383 2.385791 2.074576

27 6 0 -5.745662 -1.507319 -0.897070

28 6 0 1.189659 -1.351418 -0.946706

29 1 0 -0.977263 -0.426731 0.149332

30 1 0 -2.242459 1.127907 -1.124873

31 8 0 -7.006380 -0.981239 -1.398545

32 6 0 -4.938915 -1.855414 -2.144836

33 8 0 -3.079440 2.886375 -0.477862

34 8 0 -5.132830 0.848090 -0.834991

35 6 0 -6.016287 -2.744819 -0.041621

36 1 0 8.626824 -1.349659 -0.385658

37 1 0 8.386168 0.450124 -2.063109

38 1 0 6.177231 1.482756 -2.512891

39 1 0 6.673344 -2.157924 0.899396

40 1 0 3.485463 1.368040 -1.679109

41 1 0 3.670208 -2.023141 2.382138

42 1 0 3.039960 -2.875404 0.977775

43 1 0 -1.405836 -1.004734 2.493945

44 1 0 -0.156775 0.087239 3.032929

45 1 0 0.229355 -2.457363 1.367893

46 1 0 0.761238 -2.159355 3.012783

47 1 0 1.317384 2.106147 -1.339679

48 1 0 0.158238 0.859903 -1.760251

49 1 0 -0.937413 3.021399 -1.909597

50 1 0 -0.494561 3.461902 -0.278579

51 1 0 -5.756567 -0.217209 0.819748

52 1 0 -3.606589 -1.537607 0.920520

53 1 0 -3.042373 -0.656672 -0.478388

54 1 0 -3.961808 1.335840 1.380208

55 1 0 -3.127102 0.185188 2.367053

56 1 0 1.217295 1.702107 2.355356

57 1 0 1.036680 2.917835 1.110807

58 1 0 2.466308 1.907620 1.138377

59 1 0 -1.632835 1.943088 3.071891

60 1 0 -0.836405 3.020243 1.933264

61 1 0 -2.583277 3.037088 2.063658

62 1 0 1.354045 -0.953724 -1.950024

63 1 0 0.134996 -1.615975 -0.867432

64 1 0 1.762042 -2.278956 -0.881845

65 1 0 -7.680457 -1.059188 -0.711291

66 1 0 -5.501869 -2.559367 -2.760648

67 1 0 -3.984469 -2.316428 -1.882416

68 1 0 -4.738110 -0.964324 -2.741357

69 1 0 -3.904180 2.395044 -0.617645

70 1 0 -5.985208 0.873634 -1.294768

71 1 0 -6.614107 -2.497686 0.843187

72 1 0 -6.562581 -3.490163 -0.624714

73 1 0 -5.090519 -3.206846 0.307462

---------------------------------------------------------------------

**C3** 1.20%


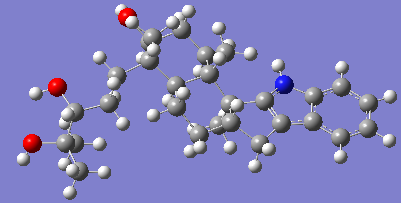


E= -1372.4894933 a.u.

Standard orientation:

---------------------------------------------------------------------

Center Atomic Atomic Coordinates (Angstroms)

Number Number Type X Y Z

---------------------------------------------------------------------

1 6 0 7.532419 -1.292195 -0.271735

2 6 0 7.553250 -0.213660 -1.175176

3 6 0 6.393118 0.488652 -1.481796

4 6 0 5.207139 0.085343 -0.868299

5 6 0 5.164318 -0.996429 0.064304

6 6 0 6.353994 -1.686402 0.347836

7 7 0 3.918473 0.587133 -1.002684

8 6 0 3.090845 -0.112843 -0.146428

9 6 0 3.800811 -1.086861 0.500511

10 6 0 1.594081 -0.245730 0.044904

11 6 0 1.637676 -1.039186 1.399528

12 6 0 2.886844 -1.958374 1.325931

13 6 0 0.707566 1.049110 0.249274

14 6 0 -0.730100 0.548302 0.679315

15 6 0 -0.695726 -0.366102 1.927040

16 6 0 0.265201 -1.566375 1.788058

17 6 0 0.568359 1.839739 -1.072847

18 6 0 -0.476948 2.957235 -0.998619

19 6 0 -1.851305 2.418108 -0.602597

20 6 0 -1.864410 1.637657 0.739604

21 6 0 -5.091196 -0.587496 0.043983

22 6 0 -3.612098 -0.212052 -0.034203

23 6 0 -3.269709 0.967655 0.889236

24 1 0 1.907737 -0.299227 2.159019

25 6 0 1.392577 1.966989 1.295849

26 6 0 -1.738810 2.615620 1.926348

27 6 0 -5.471038 -1.923691 -0.649611

28 6 0 1.114263 -1.146720 -1.130090

29 1 0 -1.043850 -0.094478 -0.147832

30 1 0 -2.176491 1.725203 -1.391303

31 8 0 -6.916096 -1.935804 -0.512853

32 6 0 -5.098289 -1.942710 -2.133425

33 8 0 -2.805147 3.485530 -0.507829

34 8 0 -5.833168 0.486301 -0.527000

35 6 0 -4.898685 -3.134438 0.091987

36 1 0 8.455356 -1.821314 -0.056534

37 1 0 8.489345 0.074230 -1.642649

38 1 0 6.413534 1.318516 -2.182338

39 1 0 6.353061 -2.517714 1.046252

40 1 0 3.673183 1.407631 -1.530687

41 1 0 3.266802 -2.195752 2.324970

42 1 0 2.676755 -2.917572 0.832627

43 1 0 -1.703328 -0.732108 2.138507

44 1 0 -0.400967 0.209787 2.810506

45 1 0 -0.112988 -2.280667 1.047064

46 1 0 0.313846 -2.108159 2.739816

47 1 0 1.537315 2.270623 -1.355981

48 1 0 0.286928 1.161816 -1.884461

49 1 0 -0.558555 3.445874 -1.977639

50 1 0 -0.183233 3.742494 -0.297665

51 1 0 -5.381436 -0.701321 1.100158

52 1 0 -3.010303 -1.088303 0.223442

53 1 0 -3.387277 0.032217 -1.076921

54 1 0 -4.010678 1.752596 0.731358

55 1 0 -3.395215 0.641455 1.927758

56 1 0 1.151657 1.705841 2.325848

57 1 0 1.114281 3.012084 1.166693

58 1 0 2.480045 1.915080 1.197063

59 1 0 -1.689476 2.073964 2.873945

60 1 0 -0.862724 3.259379 1.868596

61 1 0 -2.614172 3.265340 1.962316

62 1 0 1.383287 -0.710008 -2.093960

63 1 0 0.039532 -1.330326 -1.140612

64 1 0 1.608731 -2.118963 -1.080901

65 1 0 -7.288477 -2.627870 -1.071936

66 1 0 -5.507369 -2.834660 -2.621438

67 1 0 -4.014959 -1.969360 -2.272813

68 1 0 -5.490233 -1.059937 -2.640855

69 1 0 -3.058300 3.763994 -1.394678

70 1 0 -6.758757 0.206558 -0.543154

71 1 0 -5.178840 -3.107863 1.147681

72 1 0 -5.293220 -4.062449 -0.334255

73 1 0 -3.809452 -3.183655 0.021960

---------------------------------------------------------------------

**C4** 1.08%


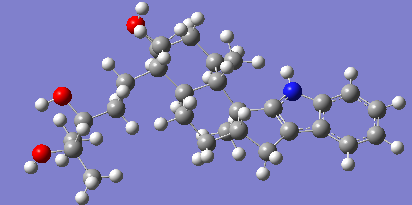


E= -1372.4893927 a.u.

Standard orientation:

---------------------------------------------------------------------

Center Atomic Atomic Coordinates (Angstroms)

Number Number Type X Y Z

---------------------------------------------------------------------

1 6 0 7.557553 -1.288615 -0.205089

2 6 0 7.595834 -0.188388 -1.081441

3 6 0 6.441217 0.519021 -1.396760

4 6 0 5.242861 0.098548 -0.819844

5 6 0 5.181863 -1.005870 0.084652

6 6 0 6.366611 -1.700232 0.378042

7 7 0 3.956659 0.602281 -0.969195

8 6 0 3.111677 -0.120489 -0.149668

9 6 0 3.809244 -1.109305 0.488203

10 6 0 1.611565 -0.257837 0.008766

11 6 0 1.627525 -1.086079 1.342650

12 6 0 2.879759 -2.001310 1.273243

13 6 0 0.722763 1.032748 0.229488

14 6 0 -0.724203 0.527357 0.621212

15 6 0 -0.719831 -0.433370 1.834673

16 6 0 0.247941 -1.626388 1.683088

17 6 0 0.610126 1.854644 -1.076059

18 6 0 -0.440428 2.968103 -1.008083

19 6 0 -1.820935 2.424576 -0.640969

20 6 0 -1.849307 1.626580 0.697823

21 6 0 -5.065410 -0.673277 0.181023

22 6 0 -3.667789 -0.135157 -0.124250

23 6 0 -3.261465 0.976746 0.853978

24 1 0 1.877952 -0.366205 2.127803

25 6 0 1.390601 1.922494 1.311193

26 6 0 -1.708395 2.584354 1.901199

27 6 0 -5.515404 -1.877052 -0.690157

28 6 0 1.152376 -1.126617 -1.198079

29 1 0 -1.031240 -0.081578 -0.233790

30 1 0 -2.128743 1.742970 -1.439447

31 8 0 -6.876683 -2.087563 -0.229779

32 6 0 -5.528423 -1.554170 -2.185582

33 8 0 -2.807395 3.459940 -0.690781

34 8 0 -5.987274 0.401484 0.039870

35 6 0 -4.698154 -3.136561 -0.392522

36 1 0 8.476794 -1.820971 0.017754

37 1 0 8.541391 0.112453 -1.520852

38 1 0 6.475188 1.365708 -2.076315

39 1 0 6.352123 -2.548233 1.055927

40 1 0 3.719768 1.429864 -1.489895

41 1 0 3.239058 -2.261924 2.274125

42 1 0 2.681869 -2.948804 0.753121

43 1 0 -1.731286 -0.810734 2.001719

44 1 0 -0.453302 0.110178 2.747631

45 1 0 -0.110725 -2.319796 0.913362

46 1 0 0.274509 -2.195362 2.619695

47 1 0 1.584226 2.293520 -1.327622

48 1 0 0.347870 1.196325 -1.909280

49 1 0 -0.518292 3.467329 -1.979115

50 1 0 -0.142167 3.744755 -0.294099

51 1 0 -5.088879 -1.030550 1.222992

52 1 0 -2.963305 -0.970969 -0.080971

53 1 0 -3.659497 0.241855 -1.151181

54 1 0 -3.995310 1.779019 0.767303

55 1 0 -3.359388 0.595206 1.876768

56 1 0 1.124354 1.641952 2.329973

57 1 0 1.125254 2.973046 1.196807

58 1 0 2.479526 1.863822 1.236839

59 1 0 -1.587711 2.030685 2.834638

60 1 0 -0.865060 3.270404 1.823681

61 1 0 -2.615789 3.184103 2.016432

62 1 0 1.444422 -0.668546 -2.145111

63 1 0 0.077032 -1.303139 -1.236098

64 1 0 1.640159 -2.102835 -1.162221

65 1 0 -7.336993 -2.676030 -0.839719

66 1 0 -5.998143 -2.368556 -2.749006

67 1 0 -4.516244 -1.436026 -2.579737

68 1 0 -6.081826 -0.634076 -2.378753

69 1 0 -2.538309 4.181619 -0.108988

70 1 0 -6.871114 0.024210 0.149236

71 1 0 -4.701177 -3.354956 0.678079

72 1 0 -5.129308 -3.999727 -0.909543

73 1 0 -3.662100 -3.043589 -0.726626

---------------------------------------------------------------------

**C5** 0.44%


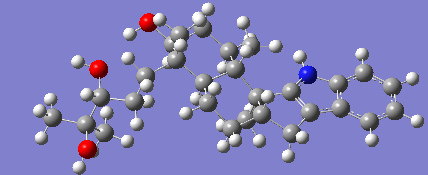


E= -1372.4885366

Standard orientation:

---------------------------------------------------------------------

Center Atomic Atomic Coordinates (Angstroms)

Number Number Type X Y Z

---------------------------------------------------------------------

1 6 0 7.658355 -0.935958 -0.530960

2 6 0 7.542021 0.115504 -1.458866

3 6 0 6.314018 0.715868 -1.713636

4 6 0 5.200182 0.238228 -1.022997

5 6 0 5.296801 -0.817566 -0.064564

6 6 0 6.551845 -1.403842 0.165208

7 7 0 3.870472 0.634712 -1.091298

8 6 0 3.151250 -0.103857 -0.172601

9 6 0 3.972294 -1.001612 0.453594

10 6 0 1.682738 -0.343276 0.115119

11 6 0 1.870926 -1.092913 1.483388

12 6 0 3.180295 -1.916371 1.353911

13 6 0 0.714309 0.885783 0.348738

14 6 0 -0.654724 0.287210 0.880397

15 6 0 -0.474093 -0.590558 2.143059

16 6 0 0.571212 -1.712748 1.969537

17 6 0 0.447599 1.645620 -0.974930

18 6 0 -0.702537 2.657067 -0.889622

19 6 0 -1.982835 1.970405 -0.434505

20 6 0 -1.851248 1.302706 0.969455

21 6 0 -5.146995 -0.342862 -0.063435

22 6 0 -3.680542 -0.526102 0.316027

23 6 0 -3.192218 0.571107 1.288151

24 1 0 2.128717 -0.313461 2.207122

25 6 0 1.393539 1.875533 1.334092

26 6 0 -1.705313 2.369856 2.072126

27 6 0 -5.771557 -1.529960 -0.844768

28 6 0 1.201781 -1.311591 -1.004213

29 1 0 -0.972639 -0.404562 0.093768

30 1 0 -2.180607 1.169427 -1.160193

31 8 0 -5.725564 -2.608912 0.108706

32 6 0 -7.235976 -1.228975 -1.193455

33 8 0 -3.057923 2.905185 -0.505547

34 8 0 -5.230450 0.878284 -0.830400

35 6 0 -4.983807 -1.891706 -2.106766

36 1 0 8.631335 -1.384605 -0.357127

37 1 0 8.424466 0.463238 -1.986227

38 1 0 6.228898 1.525573 -2.432636

39 1 0 6.656539 -2.212647 0.882041

40 1 0 3.527043 1.410149 -1.632474

41 1 0 3.636728 -2.095738 2.332957

42 1 0 3.016502 -2.902527 0.897638

43 1 0 -1.432120 -1.041211 2.413801

44 1 0 -0.183219 0.027933 2.999288

45 1 0 0.204211 -2.473252 1.270258

46 1 0 0.720749 -2.224014 2.927787

47 1 0 1.362476 2.163144 -1.291101

48 1 0 0.207879 0.934122 -1.770942

49 1 0 -0.882274 3.098911 -1.874513

50 1 0 -0.466502 3.491962 -0.223414

51 1 0 -5.740437 -0.229235 0.853176

52 1 0 -3.566497 -1.519544 0.753211

53 1 0 -3.095898 -0.528544 -0.606905

54 1 0 -3.953411 1.353404 1.345945

55 1 0 -3.139986 0.160420 2.299405

56 1 0 1.206214 1.648167 2.383316

57 1 0 1.060279 2.899721 1.169710

58 1 0 2.477493 1.872282 1.192848

59 1 0 -1.658552 1.906539 3.061709

60 1 0 -0.822850 2.994605 1.962577

61 1 0 -2.572179 3.033435 2.056567

62 1 0 1.378749 -0.886605 -1.994218

63 1 0 0.145106 -1.572652 -0.942738

64 1 0 1.768774 -2.243717 -0.960194

65 1 0 -6.079515 -3.408435 -0.299760

66 1 0 -7.718252 -2.118518 -1.610969

67 1 0 -7.324926 -0.441036 -1.946428

68 1 0 -7.797775 -0.937861 -0.301168

69 1 0 -3.874309 2.399607 -0.639091

70 1 0 -6.138868 1.199947 -0.830600

71 1 0 -3.986391 -2.256174 -1.856612

72 1 0 -5.497663 -2.680382 -2.666905

73 1 0 -4.883014 -1.027236 -2.766815

---------------------------------------------------------------------

Lowest energy conformers of compound **(3S,4S,7S,9S,10R,13R,16S)-3**

**C1** 55.71%


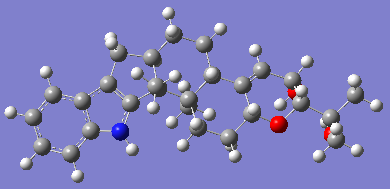


E= -1330.7385871 a.u.

Standard orientation:

---------------------------------------------------------------------

Center Atomic Atomic Coordinates (Angstroms)

Number Number Type X Y Z

---------------------------------------------------------------------

1 6 0 -7.568856 -0.754967 -0.714985

2 6 0 -7.162809 -2.095057 -0.575256

3 6 0 -5.843552 -2.419001 -0.279947

4 6 0 -4.936562 -1.371103 -0.121666

5 6 0 -5.322528 -0.004138 -0.272805

6 6 0 -6.663872 0.287502 -0.568391

7 7 0 -3.581449 -1.403322 0.185148

8 6 0 -3.106704 -0.106213 0.180520

9 6 0 -4.129291 0.766169 -0.073813

10 6 0 -1.840381 0.586745 0.638212

11 6 0 -2.130393 1.984677 -0.022394

12 6 0 -3.665417 2.194290 0.063395

13 6 0 -0.436152 0.065827 0.152736

14 6 0 0.630530 1.178349 0.504201

15 6 0 0.272302 2.595539 0.011753

16 6 0 -1.140277 3.046191 0.432717

17 6 0 -0.013590 -1.222926 0.905238

18 6 0 1.412380 -1.689127 0.588263

19 6 0 2.450464 -0.582598 0.795812

20 6 0 2.031295 0.722340 0.143559

21 8 0 3.686049 -1.081012 0.301004

22 6 0 4.708188 -0.089201 0.237323

23 6 0 4.314600 0.935492 -0.837996

24 6 0 2.897492 1.396222 -0.621192

25 8 0 4.492995 0.430089 -2.183068

26 1 0 -1.953318 1.844081 -1.092459

27 6 0 -0.452101 -0.231356 -1.361949

28 6 0 6.087381 -0.780660 0.058787

29 6 0 -1.939090 0.668079 2.189500

30 1 0 0.639350 1.232301 1.602041

31 1 0 2.555105 -0.394224 1.881206

32 6 0 6.318347 -1.779736 1.192708

33 6 0 7.201724 0.280595 0.049038

34 8 0 6.145721 -1.546843 -1.145893

35 1 0 -8.607129 -0.536621 -0.943594

36 1 0 -7.891504 -2.889776 -0.697627

37 1 0 -5.536001 -3.454890 -0.170320

38 1 0 -6.989125 1.316906 -0.684017

39 1 0 -3.033691 -2.242101 0.276381

40 1 0 -4.019529 2.863843 -0.727151

41 1 0 -3.979631 2.640184 1.017108

42 1 0 1.020334 3.298506 0.392130

43 1 0 0.340558 2.641529 -1.081121

44 1 0 -1.189962 3.207008 1.515610

45 1 0 -1.367412 4.012062 -0.032265

46 1 0 -0.707207 -2.038915 0.668676

47 1 0 -0.095362 -1.059812 1.983187

48 1 0 1.680077 -2.541465 1.219459

49 1 0 1.494670 -2.038501 -0.443818

50 1 0 4.743634 0.450827 1.198003

51 1 0 4.987811 1.794421 -0.787801

52 1 0 2.611448 2.318109 -1.117536

53 1 0 3.758476 -0.169590 -2.369041

54 1 0 0.507366 -0.622796 -1.700726

55 1 0 -0.656362 0.648279 -1.971335

56 1 0 -1.216316 -0.974755 -1.604161

57 1 0 -1.068538 1.125734 2.662613

58 1 0 -2.068468 -0.323426 2.627681

59 1 0 -2.812652 1.254163 2.481054

60 1 0 5.535266 -2.537024 1.196631

61 1 0 6.333628 -1.281181 2.166412

62 1 0 7.279694 -2.276883 1.045799

63 1 0 7.130054 0.934872 -0.823693

64 1 0 8.170691 -0.220517 0.001606

65 1 0 7.185614 0.905941 0.947868

66 1 0 5.851685 -0.972501 -1.869387

---------------------------------------------------------------------

**C2** 41.75%


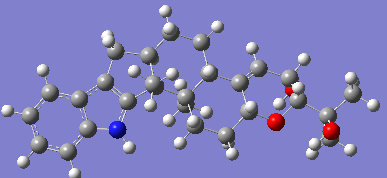


E= -1330.738315 a.u.

Standard orientation:

---------------------------------------------------------------------

Center Atomic Atomic Coordinates (Angstroms)

Number Number Type X Y Z

---------------------------------------------------------------------

1 6 0 -7.568412 -0.764766 -0.688632

2 6 0 -7.159792 -2.103579 -0.544377

3 6 0 -5.838388 -2.424224 -0.255039

4 6 0 -4.931930 -1.374330 -0.107394

5 6 0 -5.320539 -0.008663 -0.263372

6 6 0 -6.663966 0.279633 -0.552692

7 7 0 -3.575112 -1.403110 0.192079

8 6 0 -3.102085 -0.105331 0.178300

9 6 0 -4.127282 0.764295 -0.074929

10 6 0 -1.834707 0.591847 0.626710

11 6 0 -2.129917 1.986050 -0.039433

12 6 0 -3.664840 2.193765 0.053126

13 6 0 -0.431616 0.071105 0.137776

14 6 0 0.635220 1.186577 0.478263

15 6 0 0.272314 2.600056 -0.021197

16 6 0 -1.138667 3.051064 0.404933

17 6 0 -0.004610 -1.212702 0.896145

18 6 0 1.421274 -1.677671 0.576326

19 6 0 2.455775 -0.567540 0.778375

20 6 0 2.036718 0.731577 0.115013

21 8 0 3.697061 -1.063433 0.284660

22 6 0 4.713588 -0.054826 0.200066

23 6 0 4.320574 0.949939 -0.890268

24 6 0 2.898595 1.402156 -0.656837

25 8 0 4.493495 0.474795 -2.232447

26 1 0 -1.958162 1.840294 -1.109639

27 6 0 -0.453209 -0.235204 -1.374952

28 6 0 6.076575 -0.780847 0.116298

29 6 0 -1.926840 0.680820 2.178038

30 1 0 0.647660 1.248175 1.575713

31 1 0 2.563935 -0.374175 1.861523

32 6 0 7.218763 0.229720 0.070624

33 6 0 6.184308 -1.787645 -1.034695

34 8 0 6.234551 -1.482088 1.366921

35 1 0 -8.608304 -0.548935 -0.912205

36 1 0 -7.888148 -2.899871 -0.658279

37 1 0 -5.528876 -3.459143 -0.141647

38 1 0 -6.991295 1.307993 -0.671640

39 1 0 -3.025966 -2.240721 0.285216

40 1 0 -4.023968 2.858994 -0.738784

41 1 0 -3.974998 2.643635 1.006287

42 1 0 1.021341 3.306134 0.351099

43 1 0 0.334963 2.639514 -1.114598

44 1 0 -1.182818 3.217459 1.487231

45 1 0 -1.369579 4.014189 -0.063851

46 1 0 -0.697369 -2.031502 0.666955

47 1 0 -0.081752 -1.043830 1.973487

48 1 0 1.689904 -2.528527 1.209950

49 1 0 1.499090 -2.028557 -0.455798

50 1 0 4.734810 0.497427 1.151766

51 1 0 4.987274 1.814398 -0.827549

52 1 0 2.604234 2.317981 -1.159384

53 1 0 3.853555 -0.233288 -2.381931

54 1 0 0.509839 -0.614013 -1.717921

55 1 0 -0.673009 0.638216 -1.987629

56 1 0 -1.209023 -0.990027 -1.608459

57 1 0 -1.054942 1.142138 2.644926

58 1 0 -2.053118 -0.308603 2.621930

59 1 0 -2.799940 1.267149 2.470387

60 1 0 7.140700 0.949958 0.889572

61 1 0 7.232679 0.770016 -0.878027

62 1 0 8.169865 -0.295553 0.179919

63 1 0 5.357900 -2.502500 -1.009336

64 1 0 7.118291 -2.344314 -0.926793

65 1 0 6.177764 -1.289446 -2.003899

66 1 0 5.480972 -2.080745 1.456730

---------------------------------------------------------------------

**C3** 0.77%


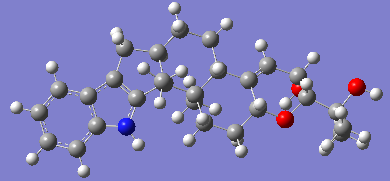


E= -1330.7345457 a.u.

Standard orientation:

---------------------------------------------------------------------

Center Atomic Atomic Coordinates (Angstroms)

Number Number Type X Y Z

---------------------------------------------------------------------

1 6 0 -7.571853 -0.759681 -0.670029

2 6 0 -7.163474 -2.099406 -0.533952

3 6 0 -5.841014 -2.422100 -0.251460

4 6 0 -4.933326 -1.373520 -0.102405

5 6 0 -5.321728 -0.006764 -0.250179

6 6 0 -6.666179 0.283562 -0.532590

7 7 0 -3.575453 -1.404489 0.191776

8 6 0 -3.101567 -0.106987 0.181709

9 6 0 -4.127176 0.764570 -0.062974

10 6 0 -1.831327 0.586835 0.627230

11 6 0 -2.128238 1.984337 -0.031347

12 6 0 -3.662629 2.193062 0.069246

13 6 0 -0.431000 0.067126 0.129576

14 6 0 0.638906 1.179760 0.471168

15 6 0 0.275096 2.595273 -0.021494

16 6 0 -1.133523 3.046382 0.412690

17 6 0 -0.002744 -1.222247 0.878217

18 6 0 1.422161 -1.686549 0.552243

19 6 0 2.455895 -0.577457 0.760202

20 6 0 2.037457 0.723817 0.097794

21 8 0 3.702388 -1.074459 0.279580

22 6 0 4.699569 -0.055590 0.185714

23 6 0 4.303687 0.905238 -0.943383

24 6 0 2.895683 1.389116 -0.683127

25 8 0 4.397431 0.341577 -2.260034

26 1 0 -1.961639 1.842830 -1.102936

27 6 0 -0.460221 -0.231193 -1.384610

28 6 0 6.091065 -0.724719 0.126131

29 6 0 -1.916753 0.669117 2.179317

30 1 0 0.655346 1.236909 1.568811

31 1 0 2.555156 -0.385311 1.844882

32 6 0 6.285389 -1.678313 -1.056158

33 6 0 6.352273 -1.459992 1.448442

34 8 0 6.984282 0.402178 0.020079

35 1 0 -8.612509 -0.542131 -0.888441

36 1 0 -7.892700 -2.894789 -0.648864

37 1 0 -5.531637 -3.457752 -0.144319

38 1 0 -6.993416 1.312676 -0.645247

39 1 0 -3.025507 -2.242632 0.275080

40 1 0 -4.024857 2.862044 -0.718125

41 1 0 -3.967999 2.639044 1.025804

42 1 0 1.027024 3.298956 0.349402

43 1 0 0.332786 2.638541 -1.114992

44 1 0 -1.172765 3.208616 1.495871

45 1 0 -1.365611 4.011617 -0.051303

46 1 0 -0.697344 -2.038752 0.645802

47 1 0 -0.076596 -1.060266 1.956909

48 1 0 1.692572 -2.541247 1.179843

49 1 0 1.497434 -2.031306 -0.482310

50 1 0 4.694977 0.539419 1.112008

51 1 0 5.002387 1.742762 -0.953447

52 1 0 2.601718 2.305502 -1.184990

53 1 0 3.729920 -0.353466 -2.328499

54 1 0 0.500102 -0.611174 -1.733606

55 1 0 -0.679380 0.645991 -1.992152

56 1 0 -1.219484 -0.982474 -1.618768

57 1 0 -1.042198 1.127531 2.643969

58 1 0 -2.041927 -0.322177 2.619396

59 1 0 -2.787992 1.255027 2.478173

60 1 0 6.223681 -1.143314 -2.001419

61 1 0 5.527511 -2.465179 -1.048103

62 1 0 7.264932 -2.165129 -0.982880

63 1 0 6.275037 -0.771395 2.293647

64 1 0 7.360599 -1.886721 1.453201

65 1 0 5.641025 -2.275352 1.594242

66 1 0 7.880139 0.078094 -0.131899

---------------------------------------------------------------------

**C4** 0.64%


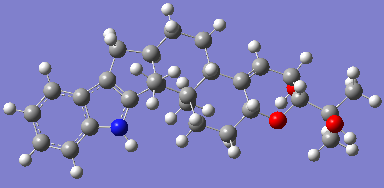


E= -1330.7343776 a.u.

Standard orientation:

---------------------------------------------------------------------

Center Atomic Atomic Coordinates (Angstroms)

Number Number Type X Y Z

---------------------------------------------------------------------

1 6 0 -7.573262 -0.760161 -0.691485

2 6 0 -7.166602 -2.099133 -0.543296

3 6 0 -5.845974 -2.420661 -0.251082

4 6 0 -4.938322 -1.371660 -0.104688

5 6 0 -5.325000 -0.005735 -0.264499

6 6 0 -6.667630 0.283432 -0.556602

7 7 0 -3.582078 -1.401346 0.196851

8 6 0 -3.107422 -0.104312 0.180756

9 6 0 -4.131070 0.766117 -0.076061

10 6 0 -1.838947 0.591781 0.627621

11 6 0 -2.131648 1.984681 -0.042398

12 6 0 -3.666367 2.195218 0.048495

13 6 0 -0.436761 0.066966 0.140680

14 6 0 0.631852 1.181651 0.478220

15 6 0 0.271522 2.594534 -0.024757

16 6 0 -1.138866 3.049299 0.399458

17 6 0 -0.011378 -1.215812 0.901972

18 6 0 1.412354 -1.686130 0.580347

19 6 0 2.451393 -0.579084 0.776390

20 6 0 2.031451 0.722038 0.116059

21 8 0 3.684443 -1.075832 0.273089

22 6 0 4.700086 -0.074565 0.217225

23 6 0 4.317209 0.927494 -0.880215

24 6 0 2.896246 1.387250 -0.657544

25 8 0 4.495222 0.420024 -2.212595

26 1 0 -1.959445 1.835655 -1.112090

27 6 0 -0.458728 -0.242765 -1.371367

28 6 0 6.081227 -0.757586 0.119435

29 6 0 -1.931519 0.685008 2.178620

30 1 0 0.644861 1.245664 1.575500

31 1 0 2.567473 -0.388800 1.859288

32 6 0 7.169943 0.268622 -0.219066

33 6 0 6.117883 -1.935652 -0.857189

34 8 0 6.298435 -1.240049 1.465585

35 1 0 -8.612541 -0.543500 -0.917241

36 1 0 -7.895777 -2.894822 -0.656367

37 1 0 -5.537945 -3.455695 -0.134599

38 1 0 -6.993504 1.311927 -0.678528

39 1 0 -3.033535 -2.239082 0.292710

40 1 0 -4.023884 2.858878 -0.745531

41 1 0 -3.976509 2.648200 1.000216

42 1 0 1.021802 3.300093 0.346118

43 1 0 0.334892 2.631244 -1.118270

44 1 0 -1.183253 3.218585 1.481331

45 1 0 -1.368231 4.011604 -0.071974

46 1 0 -0.707926 -2.032915 0.677376

47 1 0 -0.085316 -1.042983 1.978955

48 1 0 1.682467 -2.534727 1.215685

49 1 0 1.486226 -2.040864 -0.450898

50 1 0 4.714086 0.473925 1.171077

51 1 0 4.985225 1.790959 -0.832509

52 1 0 2.607640 2.303148 -1.163495

53 1 0 3.835973 -0.272469 -2.350942

54 1 0 0.504017 -0.623558 -1.712751

55 1 0 -0.677463 0.629375 -1.986380

56 1 0 -1.215191 -0.997556 -1.603204

57 1 0 -1.059007 1.146476 2.644100

58 1 0 -2.058407 -0.303205 2.624979

59 1 0 -2.804112 1.273004 2.469392

60 1 0 7.147552 1.107691 0.481559

61 1 0 7.064710 0.650327 -1.236793

62 1 0 8.155046 -0.200336 -0.136889

63 1 0 5.347155 -2.665544 -0.609514

64 1 0 7.095647 -2.429072 -0.802799

65 1 0 5.975423 -1.599913 -1.884550

66 1 0 7.001592 -1.900448 1.450270

---------------------------------------------------------------------

Lowest energy conformers of compound **(23S, 25S, 26R)-5** for NMR calculation

**C1**


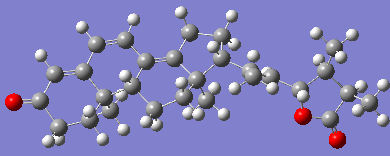


E= -1314.365947 a.u.

Standard orientation:

---------------------------------------------------------------------

Center Atomic Atomic Coordinates (Angstroms)

Number Number Type X Y Z

---------------------------------------------------------------------

1 6 0 -6.428612 -1.973538 0.385025

2 6 0 -7.303093 -0.746091 0.185059

3 6 0 -6.615653 0.498404 -0.166179

4 6 0 -5.269146 0.614293 -0.298939

5 6 0 -4.325772 -0.577498 -0.112016

6 6 0 -4.998301 -1.622354 0.805251

7 6 0 -4.675917 1.878329 -0.690157

8 6 0 -3.338324 2.081166 -0.717596

9 6 0 -2.392541 1.088749 -0.243037

10 6 0 -3.011830 -0.051410 0.558190

11 6 0 -1.060999 1.224982 -0.418897

12 6 0 -0.030890 0.239466 0.110940

13 6 0 -0.624265 -0.564638 1.278711

14 6 0 -1.999549 -1.148505 0.926547

15 6 0 -0.347899 2.419586 -1.031677

16 6 0 1.137862 2.252231 -0.635508

17 6 0 1.124387 1.231271 0.522800

18 6 0 0.424968 -0.709068 -1.021947

19 6 0 -4.037044 -1.184618 -1.507323

20 8 0 -8.520083 -0.801525 0.290225

21 6 0 2.457032 0.622805 0.935631

22 6 0 2.593706 0.384615 2.421038

23 6 0 3.418769 0.350523 0.042763

24 6 0 4.771198 -0.234548 0.288654

25 6 0 5.954266 0.647747 -0.171907

26 6 0 7.017759 -0.399471 -0.564983

27 6 0 6.163565 -1.591519 -0.995354

28 8 0 4.899631 -1.453666 -0.509599

29 8 0 6.499728 -2.543842 -1.642240

30 6 0 7.989299 -0.834341 0.544902

31 6 0 6.357560 1.713974 0.842053

32 1 0 -6.432232 -2.531798 -0.559496

33 1 0 -6.913818 -2.621712 1.118920

34 1 0 -7.259612 1.358988 -0.329161

35 1 0 -4.393198 -2.532840 0.839002

36 1 0 -5.019843 -1.224942 1.827572

37 1 0 -5.350178 2.674353 -0.994026

38 1 0 -2.957529 3.027137 -1.090892

39 1 0 -3.340474 0.410559 1.503101

40 1 0 0.052724 -1.374808 1.569981

41 1 0 -0.728369 0.091780 2.151724

42 1 0 -2.373532 -1.720987 1.779624

43 1 0 -1.890043 -1.864867 0.108418

44 1 0 -0.756094 3.355493 -0.636712

45 1 0 -0.475193 2.468069 -2.118228

46 1 0 1.717171 1.880803 -1.482589

47 1 0 1.599449 3.194882 -0.332044

48 1 0 0.738405 1.764348 1.402701

49 1 0 -0.405194 -1.327651 -1.366500

50 1 0 0.792609 -0.163827 -1.894064

51 1 0 1.226663 -1.369283 -0.682871

52 1 0 -4.962473 -1.463212 -2.014577

53 1 0 -3.513753 -0.474956 -2.150962

54 1 0 -3.421652 -2.083958 -1.426760

55 1 0 1.735889 -0.172477 2.807304

56 1 0 3.494405 -0.161576 2.699101

57 1 0 2.606406 1.339744 2.959279

58 1 0 3.239343 0.562474 -1.008083

59 1 0 4.907822 -0.529584 1.331459

60 1 0 5.636535 1.149711 -1.094366

61 1 0 7.602509 -0.074809 -1.429390

62 1 0 8.619490 -1.651428 0.188388

63 1 0 8.640706 -0.008021 0.837645

64 1 0 7.467496 -1.184799 1.440174

65 1 0 7.187992 2.319837 0.469092

66 1 0 5.522679 2.390841 1.042786

67 1 0 6.667460 1.277515 1.795096

---------------------------------------------------------------------

**C2**


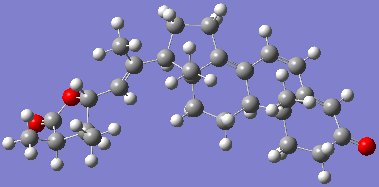


E= -1314.3653664 a.u.

Standard orientation:

---------------------------------------------------------------------

Center Atomic Atomic Coordinates (Angstroms)

Number Number Type X Y Z

---------------------------------------------------------------------

1 6 0 5.765219 -2.490909 0.450063

2 6 0 6.711407 -1.727297 -0.462848

3 6 0 6.250459 -0.422475 -0.942291

4 6 0 5.043448 0.118734 -0.636395

5 6 0 4.039241 -0.590114 0.276971

6 6 0 4.296599 -2.112518 0.236880

7 6 0 4.684437 1.435933 -1.125239

8 6 0 3.452042 1.969003 -0.957392

9 6 0 2.354742 1.218725 -0.376182

10 6 0 2.600752 -0.282247 -0.260386

11 6 0 1.177600 1.797613 -0.057053

12 6 0 -0.012713 1.034549 0.502363

13 6 0 0.091529 -0.450242 0.126823

14 6 0 1.471082 -1.027082 0.471739

15 6 0 0.772996 3.237683 -0.327272

16 6 0 -0.765034 3.266729 -0.156761

17 6 0 -1.196855 1.784116 -0.212996

18 6 0 -0.071354 1.198389 2.039770

19 6 0 4.229697 -0.044820 1.713818

20 8 0 7.810615 -2.171263 -0.762011

21 6 0 -2.614410 1.467154 0.246438

22 6 0 -3.213094 2.314626 1.344327

23 6 0 -3.278356 0.479753 -0.373960

24 6 0 -4.656921 -0.030890 -0.108218

25 6 0 -4.733795 -1.519391 0.304381

26 6 0 -6.099161 -1.954265 -0.268014

27 6 0 -6.263192 -1.025184 -1.470683

28 8 0 -5.421773 0.037834 -1.352400

29 8 0 -7.015163 -1.146240 -2.397040

30 6 0 -7.311496 -1.806946 0.666521

31 6 0 -4.484192 -1.758573 1.790297

32 1 0 6.076027 -2.287382 1.482367

33 1 0 5.929550 -3.559759 0.292843

34 1 0 6.949912 0.117686 -1.575288

35 1 0 3.677334 -2.614272 0.985914

36 1 0 3.973689 -2.493900 -0.739684

37 1 0 5.458871 2.012023 -1.624274

38 1 0 3.270904 2.988539 -1.284255

39 1 0 2.602760 -0.649057 -1.299400

40 1 0 -0.693675 -1.026297 0.627382

41 1 0 -0.081061 -0.558694 -0.950862

42 1 0 1.495242 -2.085895 0.200081

43 1 0 1.622131 -0.991437 1.553883

44 1 0 1.059791 3.535273 -1.340856

45 1 0 1.268695 3.943732 0.347358

46 1 0 -1.033369 3.716144 0.801372

47 1 0 -1.258061 3.861300 -0.929655

48 1 0 -1.130165 1.474019 -1.262731

49 1 0 0.824412 0.791148 2.511152

50 1 0 -0.135082 2.246005 2.342490

51 1 0 -0.935444 0.671335 2.454606

52 1 0 5.255004 -0.193432 2.057851

53 1 0 4.020285 1.025365 1.766061

54 1 0 3.568475 -0.552989 2.420494

55 1 0 -3.347824 3.345398 1.000335

56 1 0 -4.186364 1.954676 1.674844

57 1 0 -2.567376 2.363546 2.224008

58 1 0 -2.783266 -0.040025 -1.193304

59 1 0 -5.190154 0.587356 0.617754

60 1 0 -3.960135 -2.048272 -0.266094

61 1 0 -6.070827 -2.982113 -0.638326

62 1 0 -8.229651 -2.035398 0.121939

63 1 0 -7.238955 -2.496088 1.510816

64 1 0 -7.407643 -0.793492 1.066719

65 1 0 -4.533135 -2.823557 2.033449

66 1 0 -3.490402 -1.403035 2.075735

67 1 0 -5.214003 -1.241431 2.418660

---------------------------------------------------------------------

C3


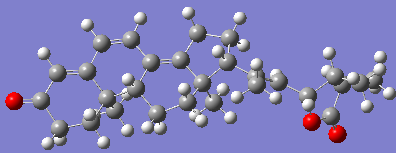


E= -1314.3650003 a.u.

Standard orientation:

---------------------------------------------------------------------

Center Atomic Atomic Coordinates (Angstroms)

Number Number Type X Y Z

---------------------------------------------------------------------

1 6 0 -6.462429 -1.885923 0.115823

2 6 0 -7.295221 -0.624555 -0.046145

3 6 0 -6.561375 0.617541 -0.297499

4 6 0 -5.207917 0.702296 -0.366757

5 6 0 -4.304236 -0.523946 -0.209114

6 6 0 -5.042499 -1.601308 0.615080

7 6 0 -4.565721 1.969490 -0.657441

8 6 0 -3.223998 2.138439 -0.610085

9 6 0 -2.325516 1.096885 -0.149588

10 6 0 -3.008701 -0.074113 0.547933

11 6 0 -0.983618 1.211420 -0.245900

12 6 0 -0.003195 0.172724 0.276216

13 6 0 -0.675762 -0.695444 1.351220

14 6 0 -2.040657 -1.219705 0.885239

15 6 0 -0.213753 2.424057 -0.744784

16 6 0 1.242177 2.204092 -0.269494

17 6 0 1.141831 1.113287 0.818271

18 6 0 0.506240 -0.708210 -0.888599

19 6 0 -3.968607 -1.056827 -1.624317

20 8 0 -8.516499 -0.650787 0.008044

21 6 0 2.433629 0.448130 1.271713

22 6 0 2.460577 0.091826 2.739741

23 6 0 3.454291 0.227529 0.431402

24 6 0 4.771868 -0.419779 0.734798

25 6 0 6.014758 0.470816 0.450759

26 6 0 6.329002 0.113697 -1.018055

27 6 0 5.826412 -1.324150 -1.139773

28 8 0 4.945232 -1.583089 -0.138215

29 8 0 6.113067 -2.140616 -1.970540

30 6 0 7.755994 0.297450 -1.527013

31 6 0 7.151123 0.167318 1.432371

32 1 0 -6.439633 -2.386931 -0.859965

33 1 0 -6.995437 -2.562266 0.788542

34 1 0 -7.174948 1.504037 -0.437035

35 1 0 -4.463755 -2.529275 0.618195

36 1 0 -5.097150 -1.264731 1.657790

37 1 0 -5.205163 2.799310 -0.945800

38 1 0 -2.804124 3.093943 -0.909550

39 1 0 -3.367659 0.333987 1.506625

40 1 0 -0.815701 -0.098300 2.261024

41 1 0 -0.031446 -1.538808 1.621292

42 1 0 -2.471281 -1.844558 1.672137

43 1 0 -1.902397 -1.874867 0.021437

44 1 0 -0.628965 3.344837 -0.322852

45 1 0 -0.272910 2.535604 -1.832540

46 1 0 1.867314 1.877107 -1.102121

47 1 0 1.697224 3.117213 0.121485

48 1 0 0.712583 1.598540 1.705841

49 1 0 -0.308913 -1.292739 -1.318063

50 1 0 0.928701 -0.115256 -1.703016

51 1 0 1.280479 -1.399375 -0.547384

52 1 0 -4.876962 -1.276241 -2.188575

53 1 0 -3.395879 -0.326953 -2.199653

54 1 0 -3.383349 -1.978111 -1.571229

55 1 0 1.563657 -0.464275 3.024409

56 1 0 3.324223 -0.503216 3.034621

57 1 0 2.463553 1.002888 3.349870

58 1 0 3.349798 0.533539 -0.606669

59 1 0 4.804498 -0.818695 1.746493

60 1 0 5.738580 1.524586 0.542570

61 1 0 5.661075 0.702181 -1.662305

62 1 0 7.823101 -0.016654 -2.570038

63 1 0 8.060594 1.345632 -1.465346

64 1 0 8.472377 -0.300176 -0.960379

65 1 0 8.034040 0.777057 1.228235

66 1 0 6.835857 0.382053 2.457449

67 1 0 7.455414 -0.883375 1.394166

---------------------------------------------------------------------

C4


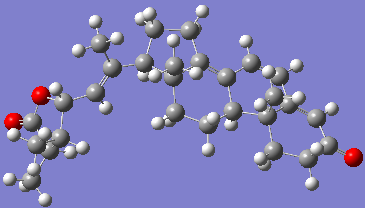


E= -1314.3645046 a.u.

Standard orientation:

---------------------------------------------------------------------

Center Atomic Atomic Coordinates (Angstroms)

Number Number Type X Y Z

---------------------------------------------------------------------

1 6 0 5.779272 -2.349109 0.767138

2 6 0 6.648255 -1.795311 -0.350699

3 6 0 6.141517 -0.613086 -1.050853

4 6 0 4.955621 -0.012687 -0.773058

5 6 0 4.028358 -0.513228 0.338043

6 6 0 4.295467 -2.013536 0.589480

7 6 0 4.550269 1.179883 -1.491995

8 6 0 3.330218 1.745789 -1.342143

9 6 0 2.286401 1.138146 -0.537998

10 6 0 2.551279 -0.310385 -0.140614

11 6 0 1.133470 1.780143 -0.252471

12 6 0 -0.006364 1.155062 0.537354

13 6 0 0.080204 -0.376160 0.463711

14 6 0 1.486679 -0.881874 0.811138

15 6 0 0.700377 3.140966 -0.773287

16 6 0 -0.821288 3.215353 -0.499298

17 6 0 -1.244359 1.754012 -0.227589

18 6 0 0.047561 1.624090 2.010722

19 6 0 4.320111 0.313993 1.614228

20 8 0 7.725737 -2.298761 -0.634270

21 6 0 -2.623849 1.543520 0.381506

22 6 0 -3.139164 2.577879 1.353329

23 6 0 -3.337022 0.476691 -0.013682

24 6 0 -4.704405 0.059942 0.435194

25 6 0 -4.853897 -1.463355 0.697988

26 6 0 -5.298765 -1.978608 -0.688279

27 6 0 -6.050275 -0.784518 -1.274867

28 8 0 -5.680068 0.347779 -0.622968

29 8 0 -6.842466 -0.779088 -2.175818

30 6 0 -6.083764 -3.284667 -0.770141

31 6 0 -5.853834 -1.741468 1.824426

32 1 0 6.163036 -1.939120 1.709766

33 1 0 5.941617 -3.428411 0.821363

34 1 0 6.787716 -0.217800 -1.830624

35 1 0 3.737120 -2.350068 1.467845

36 1 0 3.905832 -2.582975 -0.263359

37 1 0 5.279950 1.636122 -2.155262

38 1 0 3.116520 2.678803 -1.854866

39 1 0 2.477449 -0.880263 -1.080737

40 1 0 -0.658769 -0.832409 1.130611

41 1 0 -0.173904 -0.700654 -0.552875

42 1 0 1.498250 -1.973880 0.758346

43 1 0 1.720387 -0.629832 1.848974

44 1 0 0.910629 3.228931 -1.843769

45 1 0 1.239595 3.962515 -0.289859

46 1 0 -1.020588 3.850038 0.366550

47 1 0 -1.374558 3.647096 -1.336698

48 1 0 -1.248202 1.240434 -1.196508

49 1 0 0.976881 1.307864 2.486739

50 1 0 0.002708 2.711553 2.101774

51 1 0 -0.782029 1.200086 2.583781

52 1 0 5.373650 0.250287 1.892361

53 1 0 4.085242 1.369877 1.466788

54 1 0 3.733609 -0.046948 2.462854

55 1 0 -3.340280 3.519313 0.831279

56 1 0 -4.067121 2.280247 1.839813

57 1 0 -2.412839 2.805101 2.136062

58 1 0 -2.897454 -0.170908 -0.771936

59 1 0 -5.043582 0.638830 1.291924

60 1 0 -3.883257 -1.884987 0.973702

61 1 0 -4.403562 -2.082883 -1.316798

62 1 0 -6.342492 -3.497473 -1.808834

63 1 0 -5.493179 -4.121293 -0.387100

64 1 0 -7.017041 -3.239098 -0.205730

65 1 0 -5.974565 -2.812698 2.000191

66 1 0 -5.508389 -1.293173 2.760370

67 1 0 -6.842449 -1.326534 1.604605

---------------------------------------------------------------------

C5


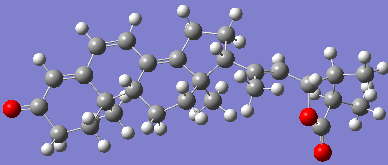


E= -1314.3644194 a.u.

Standard orientation:

---------------------------------------------------------------------

Center Atomic Atomic Coordinates (Angstroms)

Number Number Type X Y Z

---------------------------------------------------------------------

1 6 0 -6.298881 -2.022078 0.129734

2 6 0 -7.192509 -0.805808 -0.053981

3 6 0 -6.519715 0.469330 -0.312127

4 6 0 -5.171640 0.620703 -0.373222

5 6 0 -4.209854 -0.557885 -0.197553

6 6 0 -4.898604 -1.661266 0.634516

7 6 0 -4.590636 1.915278 -0.672850

8 6 0 -3.258897 2.150021 -0.621637

9 6 0 -2.313339 1.156585 -0.148468

10 6 0 -2.943395 -0.035380 0.561437

11 6 0 -0.978243 1.334874 -0.243321

12 6 0 0.052330 0.349366 0.288840

13 6 0 -0.583259 -0.525935 1.381824

14 6 0 -1.921835 -1.123191 0.926397

15 6 0 -0.274294 2.585038 -0.744342

16 6 0 1.197930 2.423786 -0.304736

17 6 0 1.169365 1.343990 0.796376

18 6 0 0.599787 -0.531274 -0.859578

19 6 0 -3.840638 -1.088432 -1.604834

20 8 0 -8.411360 -0.892931 -0.010827

21 6 0 2.497017 0.738540 1.218717

22 6 0 2.503982 0.043782 2.560882

23 6 0 3.595682 0.805912 0.452091

24 6 0 4.935361 0.209349 0.793335

25 6 0 6.081132 0.619371 -0.161820

26 6 0 5.984156 -0.481527 -1.241517

27 6 0 5.468155 -1.685223 -0.454474

28 8 0 4.888592 -1.257412 0.696414

29 8 0 5.524404 -2.845859 -0.754353

30 6 0 7.215974 -0.795278 -2.085585

31 6 0 7.430362 0.673765 0.560753

32 1 0 -6.244632 -2.533843 -0.839239

33 1 0 -6.802550 -2.715524 0.807654

34 1 0 -7.175908 1.322540 -0.464853

35 1 0 -4.273925 -2.558874 0.651707

36 1 0 -4.976727 -1.316516 1.673029

37 1 0 -5.268494 2.710107 -0.971883

38 1 0 -2.884660 3.122119 -0.928260

39 1 0 -3.330125 0.370657 1.510193

40 1 0 0.092279 -1.336416 1.669276

41 1 0 -0.746654 0.081863 2.280379

42 1 0 -2.322054 -1.749475 1.728117

43 1 0 -1.749026 -1.792559 0.079660

44 1 0 -0.719705 3.480068 -0.297734

45 1 0 -0.363486 2.710765 -1.828449

46 1 0 1.805012 2.104766 -1.153813

47 1 0 1.633622 3.357542 0.058124

48 1 0 0.746137 1.817610 1.694026

49 1 0 -0.187332 -1.156202 -1.284218

50 1 0 1.005167 0.064864 -1.680290

51 1 0 1.399188 -1.185264 -0.501319

52 1 0 -4.734036 -1.366571 -2.167078

53 1 0 -3.309790 -0.333967 -2.188629

54 1 0 -3.203816 -1.973953 -1.538765

55 1 0 2.149715 -0.987662 2.475544

56 1 0 3.500957 -0.009634 3.000210

57 1 0 1.846394 0.550929 3.271845

58 1 0 3.542047 1.305688 -0.510090

59 1 0 5.199741 0.405205 1.833682

60 1 0 5.870295 1.603543 -0.590489

61 1 0 5.161533 -0.218420 -1.921229

62 1 0 6.993094 -1.605667 -2.781736

63 1 0 7.526508 0.077808 -2.666011

64 1 0 8.060707 -1.115985 -1.473107

65 1 0 8.241113 0.937594 -0.121936

66 1 0 7.409058 1.428362 1.352065

67 1 0 7.684320 -0.284358 1.024609

---------------------------------------------------------------------

Lowest energy conformers of compound **(23R, 25R, 26S)-5** for NMR calculation

**C1**


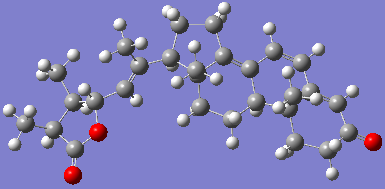


E= -1314.3659613 a.u.

Standard orientation:

---------------------------------------------------------------------

Center Atomic Atomic Coordinates (Angstroms)

Number Number Type X Y Z

---------------------------------------------------------------------

1 6 0 5.855033 -2.424266 0.431770

2 6 0 6.771270 -1.641921 -0.495284

3 6 0 6.278334 -0.344776 -0.963786

4 6 0 5.066144 0.172452 -0.637728

5 6 0 4.089532 -0.557243 0.288921

6 6 0 4.375890 -2.074240 0.243172

7 6 0 4.674930 1.483993 -1.117305

8 6 0 3.434464 1.991763 -0.931752

9 6 0 2.359521 1.218183 -0.339558

10 6 0 2.637215 -0.277794 -0.226456

11 6 0 1.173444 1.771788 -0.008497

12 6 0 0.005135 0.983514 0.561199

13 6 0 0.137449 -0.500765 0.191067

14 6 0 1.533460 -1.044145 0.522481

15 6 0 0.736248 3.203513 -0.272796

16 6 0 -0.803288 3.196018 -0.106863

17 6 0 -1.200121 1.703300 -0.150140

18 6 0 -0.050333 1.151666 2.098334

19 6 0 4.290325 -0.009909 1.723659

20 8 0 7.873414 -2.064428 -0.814557

21 6 0 -2.600180 1.347723 0.333335

22 6 0 -3.211236 2.192668 1.425915

23 6 0 -3.233657 0.317759 -0.248946

24 6 0 -4.587534 -0.234723 0.054849

25 6 0 -5.557685 -0.285067 -1.146953

26 6 0 -6.432692 -1.512205 -0.815346

27 6 0 -5.477610 -2.386662 -0.003175

28 8 0 -4.439375 -1.634957 0.454439

29 8 0 -5.580889 -3.554961 0.247583

30 6 0 -7.705949 -1.242230 0.003427

31 6 0 -6.268974 1.036266 -1.421208

32 1 0 6.178653 -2.215410 1.459099

33 1 0 6.037203 -3.489694 0.271113

34 1 0 6.957585 0.210216 -1.605976

35 1 0 3.779037 -2.588478 1.001631

36 1 0 4.044289 -2.461443 -0.728139

37 1 0 5.431266 2.077013 -1.624297

38 1 0 3.229529 3.008698 -1.252853

39 1 0 2.630846 -0.645062 -1.265409

40 1 0 -0.629355 -1.092608 0.700106

41 1 0 -0.041926 -0.617153 -0.884873

42 1 0 1.576891 -2.103402 0.255775

43 1 0 1.696285 -1.000191 1.602510

44 1 0 1.020099 3.515454 -1.282810

45 1 0 1.212363 3.916311 0.408931

46 1 0 -1.086234 3.648928 0.845351

47 1 0 -1.306984 3.771979 -0.887460

48 1 0 -1.134935 1.386978 -1.197999

49 1 0 -0.126211 2.199465 2.398751

50 1 0 -0.904403 0.612363 2.517422

51 1 0 0.853233 0.758267 2.566419

52 1 0 5.323164 -0.139106 2.052818

53 1 0 4.061075 1.056016 1.780473

54 1 0 3.649141 -0.531541 2.438760

55 1 0 -3.471925 3.186878 1.046634

56 1 0 -4.119006 1.755551 1.840646

57 1 0 -2.517206 2.349958 2.253597

58 1 0 -2.722772 -0.215835 -1.049310

59 1 0 -5.059843 0.279743 0.895216

60 1 0 -4.958045 -0.544391 -2.028477

61 1 0 -6.710768 -2.064079 -1.716768

62 1 0 -8.178787 -2.187342 0.277330

63 1 0 -8.425898 -0.659722 -0.575590

64 1 0 -7.497444 -0.696095 0.927955

65 1 0 -6.940092 0.952297 -2.280479

66 1 0 -5.544586 1.823521 -1.647092

67 1 0 -6.864247 1.368727 -0.566748

---------------------------------------------------------------------

**C2**


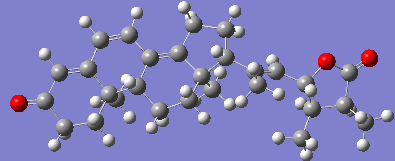


E= -1314.3654055 a.u.

Standard orientation:

---------------------------------------------------------------------

Center Atomic Atomic Coordinates (Angstroms)

Number Number Type X Y Z

---------------------------------------------------------------------

1 6 0 -6.333877 -2.050958 -0.202298

2 6 0 -7.237507 -0.835459 -0.071362

3 6 0 -6.580831 0.473305 -0.076677

4 6 0 -5.237702 0.651747 -0.165248

5 6 0 -4.264595 -0.523851 -0.293003

6 6 0 -4.911524 -1.792552 0.304490

7 6 0 -4.675312 1.987872 -0.202577

8 6 0 -3.343461 2.223089 -0.159249

9 6 0 -2.374042 1.163848 0.047271

10 6 0 -2.968019 -0.163173 0.507744

11 6 0 -1.045222 1.374270 -0.065819

12 6 0 0.007637 0.309415 0.204468

13 6 0 -0.574726 -0.793070 1.102173

14 6 0 -1.928941 -1.293617 0.581553

15 6 0 -0.359610 2.706038 -0.327062

16 6 0 1.125329 2.477637 0.040589

17 6 0 1.127038 1.185297 0.886210

18 6 0 0.507351 -0.289350 -1.130929

19 6 0 -3.955401 -0.728225 -1.797026

20 8 0 -8.452459 -0.945883 0.010499

21 6 0 2.477197 0.534646 1.155838

22 6 0 2.606321 -0.084625 2.527693

23 6 0 3.461005 0.545119 0.245980

24 6 0 4.830897 -0.043085 0.351967

25 6 0 5.127933 -1.193805 -0.638918

26 6 0 6.639269 -1.025790 -0.902573

27 6 0 6.846377 0.474095 -0.695698

28 8 0 5.799640 0.993495 0.001464

29 8 0 7.775995 1.144626 -1.048468

30 6 0 7.585515 -1.808339 0.023180

31 6 0 4.647295 -2.559210 -0.156777

32 1 0 -6.325390 -2.335839 -1.261836

33 1 0 -6.802923 -2.882975 0.328641

34 1 0 -7.245335 1.331019 -0.009316

35 1 0 -4.284552 -2.663695 0.093957

36 1 0 -4.940697 -1.686787 1.395984

37 1 0 -5.368478 2.819805 -0.291942

38 1 0 -2.986128 3.243158 -0.262548

39 1 0 -3.313423 0.021321 1.537758

40 1 0 0.122914 -1.634411 1.176226

41 1 0 -0.704165 -0.399735 2.117879

42 1 0 -2.295700 -2.086002 1.239541

43 1 0 -1.792363 -1.756191 -0.399483

44 1 0 -0.799827 3.493888 0.292487

45 1 0 -0.475129 3.036601 -1.364609

46 1 0 1.727417 2.361874 -0.861934

47 1 0 1.555432 3.315664 0.593014

48 1 0 0.712212 1.456013 1.866803

49 1 0 0.875519 0.478180 -1.815206

50 1 0 1.322322 -0.996447 -0.957257

51 1 0 -0.296027 -0.814499 -1.649569

52 1 0 -4.871414 -0.880803 -2.370818

53 1 0 -3.446426 0.139472 -2.221065

54 1 0 -3.319313 -1.602954 -1.955282

55 1 0 1.856956 -0.867400 2.681043

56 1 0 3.583506 -0.526016 2.720383

57 1 0 2.424651 0.670193 3.300834

58 1 0 3.291933 1.034341 -0.709729

59 1 0 5.065877 -0.355918 1.372125

60 1 0 4.606327 -0.952726 -1.573434

61 1 0 6.895272 -1.261468 -1.938610

62 1 0 8.620675 -1.529355 -0.182744

63 1 0 7.486918 -2.884216 -0.137424

64 1 0 7.392494 -1.606748 1.080840

65 1 0 3.565314 -2.554543 0.000605

66 1 0 5.118173 -2.850712 0.785543

67 1 0 4.867606 -3.337603 -0.892445

---------------------------------------------------------------------

**C3**


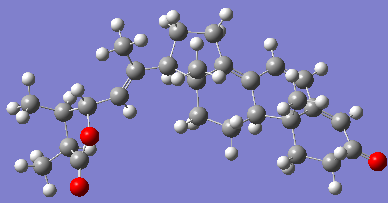


E= -1314.3651677 a.u.

Standard orientation:

---------------------------------------------------------------------

Center Atomic Atomic Coordinates (Angstroms)

Number Number Type X Y Z

---------------------------------------------------------------------

1 6 0 5.733763 -2.342222 0.782187

2 6 0 6.620078 -1.785690 -0.320263

3 6 0 6.127490 -0.597557 -1.020700

4 6 0 4.940367 0.005999 -0.755348

5 6 0 3.996191 -0.496726 0.340395

6 6 0 4.253763 -1.999503 0.586908

7 6 0 4.549854 1.204193 -1.473222

8 6 0 3.330160 1.774160 -1.336891

9 6 0 2.273073 1.166452 -0.550322

10 6 0 2.526089 -0.285212 -0.155973

11 6 0 1.119216 1.811944 -0.276641

12 6 0 -0.032870 1.187714 0.494992

13 6 0 0.046740 -0.343894 0.415359

14 6 0 1.446514 -0.856444 0.779029

15 6 0 0.699472 3.177963 -0.795190

16 6 0 -0.826916 3.255182 -0.547190

17 6 0 -1.258662 1.795118 -0.283126

18 6 0 0.002643 1.649575 1.971325

19 6 0 4.275006 0.322262 1.624819

20 8 0 7.699742 -2.291147 -0.592222

21 6 0 -2.641665 1.584838 0.318287

22 6 0 -3.163015 2.621169 1.285669

23 6 0 -3.343642 0.506028 -0.062788

24 6 0 -4.705050 0.076176 0.390374

25 6 0 -5.716677 -0.177492 -0.762689

26 6 0 -5.487440 -1.675640 -1.057678

27 6 0 -5.044588 -2.230574 0.295083

28 8 0 -4.601500 -1.213372 1.079291

29 8 0 -5.047667 -3.372124 0.663855

30 6 0 -6.616696 -2.478568 -1.697609

31 6 0 -7.146153 0.170667 -0.335880

32 1 0 6.106640 -1.939504 1.732306

33 1 0 5.890733 -3.422498 0.832407

34 1 0 6.785819 -0.200507 -1.789412

35 1 0 3.682475 -2.338715 1.455679

36 1 0 3.872961 -2.562559 -0.274052

37 1 0 5.290545 1.661141 -2.123755

38 1 0 3.127652 2.711089 -1.847187

39 1 0 2.461978 -0.850219 -1.099787

40 1 0 -0.703619 -0.801352 1.067420

41 1 0 -0.192449 -0.661946 -0.607022

42 1 0 1.453130 -1.948021 0.721715

43 1 0 1.667309 -0.610333 1.821028

44 1 0 0.929261 3.274218 -1.860962

45 1 0 1.232000 3.994176 -0.295501

46 1 0 -1.039929 3.890372 0.315054

47 1 0 -1.363762 3.689609 -1.394368

48 1 0 -1.254576 1.282867 -1.252628

49 1 0 0.921473 1.321384 2.459209

50 1 0 -0.031178 2.737310 2.067088

51 1 0 -0.838975 1.230230 2.529563

52 1 0 5.324519 0.252062 1.916444

53 1 0 4.047115 1.380103 1.480169

54 1 0 3.675693 -0.040571 2.463456

55 1 0 -3.415563 3.548741 0.760105

56 1 0 -4.056969 2.295182 1.816493

57 1 0 -2.417093 2.885245 2.037103

58 1 0 -2.886724 -0.163544 -0.791105

59 1 0 -5.120902 0.758612 1.128876

60 1 0 -5.446000 0.433214 -1.628287

61 1 0 -4.601892 -1.764675 -1.702054

62 1 0 -6.311514 -3.519670 -1.816088

63 1 0 -6.867921 -2.081929 -2.684998

64 1 0 -7.521428 -2.472139 -1.086891

65 1 0 -7.864021 -0.022745 -1.136034

66 1 0 -7.219353 1.231595 -0.080160

67 1 0 -7.462311 -0.402602 0.541287

---------------------------------------------------------------------

**C4**


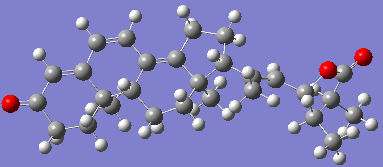


E= -1314.3645694 a.u.

Standard orientation:

---------------------------------------------------------------------

Center Atomic Atomic Coordinates (Angstroms)

Number Number Type X Y Z

---------------------------------------------------------------------

1 6 0 -6.403220 -1.955725 -0.183945

2 6 0 -7.260617 -0.700699 -0.163163

3 6 0 -6.551598 0.578359 -0.238166

4 6 0 -5.200238 0.698583 -0.294766

5 6 0 -4.271777 -0.519104 -0.308023

6 6 0 -4.988367 -1.717672 0.352540

7 6 0 -4.583246 2.006229 -0.406173

8 6 0 -3.245231 2.193156 -0.330266

9 6 0 -2.326663 1.115739 -0.013615

10 6 0 -2.987731 -0.154651 0.510342

11 6 0 -0.987284 1.271263 -0.084516

12 6 0 0.014489 0.192728 0.301526

13 6 0 -0.645998 -0.833280 1.235686

14 6 0 -1.996278 -1.315108 0.688095

15 6 0 -0.242532 2.558318 -0.402670

16 6 0 1.213858 2.308564 0.055107

17 6 0 1.128662 1.076726 0.981965

18 6 0 0.558378 -0.505067 -0.966326

19 6 0 -3.924230 -0.840638 -1.782925

20 8 0 -8.480763 -0.758312 -0.110322

21 6 0 2.436877 0.400941 1.370052

22 6 0 2.449641 -0.155884 2.774697

23 6 0 3.483619 0.342824 0.534499

24 6 0 4.825424 -0.276938 0.783512

25 6 0 5.230260 -1.383380 -0.232973

26 6 0 5.979628 -0.567909 -1.308540

27 6 0 6.547799 0.609678 -0.518414

28 8 0 5.861315 0.748907 0.645506

29 8 0 7.445193 1.343239 -0.827588

30 6 0 7.024446 -1.275204 -2.167302

31 6 0 6.073648 -2.472197 0.437948

32 1 0 -6.372791 -2.310832 -1.221660

33 1 0 -6.921408 -2.732001 0.384400

34 1 0 -7.182515 1.463545 -0.252164

35 1 0 -4.391628 -2.625316 0.224093

36 1 0 -5.047668 -1.534937 1.432614

37 1 0 -5.238694 2.855363 -0.579016

38 1 0 -2.843712 3.188876 -0.492048

39 1 0 -3.356762 0.108950 1.514621

40 1 0 0.015328 -1.692979 1.387844

41 1 0 -0.802265 -0.376484 2.220527

42 1 0 -2.417128 -2.056282 1.373008

43 1 0 -1.840651 -1.835444 -0.260594

44 1 0 -0.682973 3.402374 0.137295

45 1 0 -0.295431 2.817521 -1.465362

46 1 0 1.854618 2.114978 -0.806720

47 1 0 1.644452 3.168018 0.573121

48 1 0 0.673859 1.426477 1.918943

49 1 0 0.986735 0.205386 -1.676679

50 1 0 1.337669 -1.226128 -0.706055

51 1 0 -0.236626 -1.035804 -1.492128

52 1 0 -4.827625 -0.997293 -2.375388

53 1 0 -3.366392 -0.025912 -2.248728

54 1 0 -3.320583 -1.748818 -1.858113

55 1 0 1.655874 -0.895686 2.914937

56 1 0 3.390326 -0.628136 3.054548

57 1 0 2.255350 0.644530 3.497281

58 1 0 3.391892 0.798456 -0.448556

59 1 0 4.921436 -0.637282 1.805439

60 1 0 4.331172 -1.841709 -0.653490

61 1 0 5.233112 -0.122422 -1.980518

62 1 0 7.485446 -0.563140 -2.853920

63 1 0 6.569685 -2.074767 -2.758269

64 1 0 7.823196 -1.709530 -1.563214

65 1 0 6.378713 -3.241497 -0.274855

66 1 0 5.499636 -2.966608 1.226873

67 1 0 6.980090 -2.064048 0.896049

---------------------------------------------------------------------

**C5**


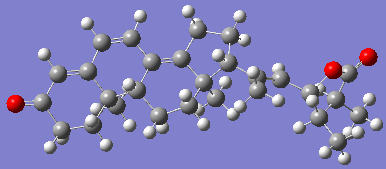


E= -1314.3645689 a.u.

Standard orientation:

---------------------------------------------------------------------

Center Atomic Atomic Coordinates (Angstroms)

Number Number Type X Y Z

---------------------------------------------------------------------

1 6 0 -6.403064 -1.955914 -0.183419

2 6 0 -7.260529 -0.700920 -0.163288

3 6 0 -6.551582 0.578156 -0.238499

4 6 0 -5.200220 0.698435 -0.294966

5 6 0 -4.271702 -0.519215 -0.307772

6 6 0 -4.988279 -1.717604 0.353118

7 6 0 -4.583289 2.006080 -0.406673

8 6 0 -3.245289 2.193103 -0.330734

9 6 0 -2.326687 1.115814 -0.013755

10 6 0 -2.987714 -0.154447 0.510554

11 6 0 -0.987313 1.271388 -0.084642

12 6 0 0.014483 0.192955 0.301618

13 6 0 -0.645941 -0.832809 1.236094

14 6 0 -1.996239 -1.314831 0.688715

15 6 0 -0.242606 2.558410 -0.403056

16 6 0 1.213826 2.308747 0.054617

17 6 0 1.128692 1.077128 0.981774

18 6 0 0.558255 -0.505144 -0.966115

19 6 0 -3.924049 -0.841174 -1.782556

20 8 0 -8.480692 -0.758609 -0.110844

21 6 0 2.436918 0.401425 1.369962

22 6 0 2.449902 -0.154503 2.774960

23 6 0 3.483498 0.342719 0.534243

24 6 0 4.825271 -0.277053 0.783398

25 6 0 5.230129 -1.383587 -0.232952

26 6 0 5.979564 -0.568229 -1.308564

27 6 0 6.547735 0.609412 -0.518517

28 8 0 5.861234 0.748751 0.645367

29 8 0 7.445189 1.342900 -0.827716

30 6 0 7.024430 -1.275650 -2.167174

31 6 0 6.073492 -2.472341 0.438097

32 1 0 -6.372528 -2.311491 -1.220970

33 1 0 -6.921298 -2.731950 0.385213

34 1 0 -7.182548 1.463301 -0.252849

35 1 0 -4.391480 -2.625258 0.225020

36 1 0 -5.047682 -1.534517 1.433127

37 1 0 -5.238769 2.855133 -0.579796

38 1 0 -2.843814 3.188803 -0.492758

39 1 0 -3.356812 0.109447 1.514732

40 1 0 0.015418 -1.692443 1.388489

41 1 0 -0.802187 -0.375720 2.220803

42 1 0 -2.417071 -2.055744 1.373922

43 1 0 -1.840646 -1.835541 -0.259774

44 1 0 -0.683010 3.402504 0.136889

45 1 0 -0.295630 2.817518 -1.465762

46 1 0 1.854493 2.114938 -0.807228

47 1 0 1.644495 3.168319 0.572375

48 1 0 0.673937 1.427103 1.918691

49 1 0 0.986693 0.205120 -1.676608

50 1 0 1.337455 -1.226263 -0.705740

51 1 0 -0.236833 -1.035861 -1.491806

52 1 0 -4.827400 -0.998109 -2.375011

53 1 0 -3.366273 -0.026531 -2.248582

54 1 0 -3.320298 -1.749311 -1.857435

55 1 0 1.655247 -0.893145 2.916191

56 1 0 3.390147 -0.627798 3.054517

57 1 0 2.257125 0.646671 3.497120

58 1 0 3.391650 0.797788 -0.449058

59 1 0 4.921214 -0.637278 1.805373

60 1 0 4.331054 -1.841956 -0.653458

61 1 0 5.233092 -0.122793 -1.980625

62 1 0 7.485447 -0.563690 -2.853888

63 1 0 6.569710 -2.075322 -2.758026

64 1 0 7.823160 -1.709859 -1.562975

65 1 0 6.378549 -3.241723 -0.274620

66 1 0 5.499467 -2.966646 1.227080

67 1 0 6.979933 -2.064144 0.896151

---------------------------------------------------------------------

Lowest energy conformers of compound **(23R, 25R, 26R)-6** for NMR calculation

**C1**


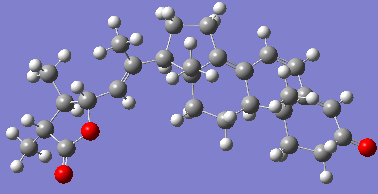


E= -1314.3693241 a.u.

Standard orientation:

---------------------------------------------------------------------

Center Atomic Atomic Coordinates (Angstroms)

Number Number Type X Y Z

---------------------------------------------------------------------

1 6 0 5.817541 -2.441216 0.508225

2 6 0 6.714831 -1.739350 -0.498498

3 6 0 6.224124 -0.471072 -1.042051

4 6 0 5.029539 0.086271 -0.716958

5 6 0 4.074222 -0.560190 0.290082

6 6 0 4.338098 -2.081467 0.340796

7 6 0 4.640856 1.365462 -1.278413

8 6 0 3.413735 1.904488 -1.092502

9 6 0 2.348282 1.193393 -0.411683

10 6 0 2.609213 -0.293993 -0.194926

11 6 0 1.178439 1.787753 -0.093172

12 6 0 0.017658 1.064181 0.569967

13 6 0 0.121017 -0.446943 0.314613

14 6 0 1.520298 -0.982516 0.645369

15 6 0 0.750660 3.198598 -0.464216

16 6 0 -0.782358 3.228141 -0.250001

17 6 0 -1.199167 1.742665 -0.161939

18 6 0 0.009345 1.352169 2.089957

19 6 0 4.333544 0.081648 1.675473

20 8 0 7.801103 -2.199564 -0.820024

21 6 0 -2.589887 1.448395 0.385892

22 6 0 -3.157906 2.384551 1.425765

23 6 0 -3.254222 0.387927 -0.099364

24 6 0 -4.611669 -0.110106 0.272557

25 6 0 -5.610403 -0.226086 -0.898890

26 6 0 -6.503036 -1.391254 -0.445987

27 6 0 -5.551085 -2.235713 0.399280

28 8 0 -4.485613 -1.479140 0.777355

29 8 0 -5.667051 -3.385574 0.719592

30 6 0 -7.203038 -2.192082 -1.539598

31 6 0 -6.330869 1.078625 -1.221711

32 1 0 -5.059127 0.477278 1.078505

33 1 0 -7.259016 -1.006127 0.255322

34 1 0 6.176230 -2.166736 1.508269

35 1 0 5.979875 -3.517982 0.416758

36 1 0 6.889627 0.026867 -1.742738

37 1 0 3.758449 -2.532825 1.150877

38 1 0 3.970530 -2.529015 -0.590834

39 1 0 5.388380 1.908046 -1.850733

40 1 0 3.211953 2.898217 -1.481063

41 1 0 2.561313 -0.737864 -1.202510

42 1 0 -0.635717 -0.985988 0.892779

43 1 0 -0.094723 -0.645855 -0.742383

44 1 0 1.542262 -2.060009 0.462256

45 1 0 1.717601 -0.855932 1.713047

46 1 0 1.005269 3.417477 -1.505960

47 1 0 1.257457 3.960235 0.137895

48 1 0 -1.026455 3.760177 0.671703

49 1 0 -1.303710 3.748535 -1.057575

50 1 0 -1.169328 1.342793 -1.182489

51 1 0 0.919400 0.979031 2.561958

52 1 0 -0.040690 2.421281 2.309697

53 1 0 -0.840931 0.863068 2.573420

54 1 0 5.378154 -0.031763 1.971650

55 1 0 4.111133 1.150593 1.669080

56 1 0 3.719645 -0.386610 2.448924

57 1 0 -3.419327 3.349447 0.977519

58 1 0 -4.056851 1.993478 1.901408

59 1 0 -2.436346 2.596505 2.216852

60 1 0 -2.771933 -0.215135 -0.867334

61 1 0 -5.048533 -0.554183 -1.783842

62 1 0 -7.735191 -3.043381 -1.111346

63 1 0 -7.926115 -1.573540 -2.077400

64 1 0 -6.483427 -2.582220 -2.264822

65 1 0 -7.012945 0.955855 -2.066977

66 1 0 -5.620941 1.867399 -1.485665

67 1 0 -6.920038 1.429465 -0.368035

---------------------------------------------------------------------

**C2**


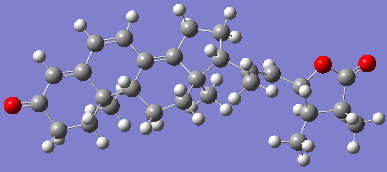


E= -1314.3689288 a.u.

Standard orientation:

---------------------------------------------------------------------

Center Atomic Atomic Coordinates (Angstroms)

Number Number Type X Y Z

---------------------------------------------------------------------

1 6 0 -6.347249 -2.037540 -0.026357

2 6 0 -7.241096 -0.807926 -0.042078

3 6 0 -6.572641 0.486305 -0.191997

4 6 0 -5.227129 0.642359 -0.287444

5 6 0 -4.264006 -0.547887 -0.270166

6 6 0 -4.927508 -1.734435 0.462531

7 6 0 -4.651548 1.960372 -0.473009

8 6 0 -3.318221 2.188889 -0.439736

9 6 0 -2.360338 1.154214 -0.098291

10 6 0 -2.970044 -0.110249 0.496651

11 6 0 -1.027913 1.345514 -0.206119

12 6 0 0.013674 0.313870 0.201880

13 6 0 -0.593895 -0.692428 1.191076

14 6 0 -1.941664 -1.234318 0.696716

15 6 0 -0.327512 2.637090 -0.597545

16 6 0 1.142447 2.455072 -0.148931

17 6 0 1.110715 1.258310 0.826518

18 6 0 0.552514 -0.414813 -1.050137

19 6 0 -3.945207 -0.925972 -1.738264

20 8 0 -8.457492 -0.898391 0.042440

21 6 0 2.445851 0.640542 1.217967

22 6 0 2.513397 0.172121 2.652756

23 6 0 3.469115 0.556047 0.356512

24 6 0 4.827760 -0.020960 0.588479

25 6 0 5.178534 -1.252107 -0.276982

26 6 0 6.700544 -1.112874 -0.430502

27 6 0 6.908550 0.398861 -0.364427

28 8 0 5.823078 0.982440 0.211031

29 8 0 7.855832 1.032732 -0.737123

30 6 0 7.333675 -1.750779 -1.663041

31 6 0 4.689143 -2.572731 0.307775

32 1 0 5.004658 -0.251038 1.642118

33 1 0 7.185210 -1.517060 0.471315

34 1 0 -6.330478 -2.439400 -1.047156

35 1 0 -6.828530 -2.801043 0.589674

36 1 0 -7.229832 1.351420 -0.230459

37 1 0 -4.307407 -2.629485 0.359513

38 1 0 -4.965673 -1.504218 1.534442

39 1 0 -5.335694 2.781774 -0.667439

40 1 0 -2.950948 3.187259 -0.656902

41 1 0 -3.320658 0.185189 1.498750

42 1 0 0.096388 -1.525629 1.362432

43 1 0 -0.741407 -0.200513 2.160297

44 1 0 -2.323933 -1.957374 1.422196

45 1 0 -1.793728 -1.789503 -0.233338

46 1 0 -0.787236 3.493645 -0.094835

47 1 0 -0.401744 2.840360 -1.671095

48 1 0 1.781827 2.250488 -1.009010

49 1 0 1.547964 3.348274 0.330902

50 1 0 0.659749 1.630031 1.756716

51 1 0 0.939567 0.281234 -1.797478

52 1 0 1.362106 -1.098836 -0.782853

53 1 0 -0.236011 -0.992013 -1.535311

54 1 0 -4.858376 -1.140925 -2.296555

55 1 0 -3.429706 -0.115228 -2.256847

56 1 0 -3.311904 -1.815309 -1.789326

57 1 0 1.755741 -0.590525 2.856751

58 1 0 3.479970 -0.242773 2.935844

59 1 0 2.300176 1.006099 3.330596

60 1 0 3.347574 0.943821 -0.651622

61 1 0 4.722348 -1.105552 -1.265326

62 1 0 8.396872 -1.509954 -1.716103

63 1 0 7.231484 -2.838701 -1.639241

64 1 0 6.865072 -1.385146 -2.581050

65 1 0 4.940845 -3.412131 -0.345474

66 1 0 3.603154 -2.572226 0.436172

67 1 0 5.141532 -2.766537 1.285919

---------------------------------------------------------------------

**C3**


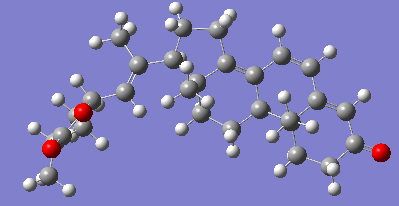


E= 1314.3653721 a.u.

Standard orientation:

---------------------------------------------------------------------

Center Atomic Atomic Coordinates (Angstroms)

Number Number Type X Y Z

---------------------------------------------------------------------

1 6 0 5.637560 -2.655498 0.063608

2 6 0 6.710145 -1.689882 -0.413900

3 6 0 6.325919 -0.279953 -0.508680

4 6 0 5.084061 0.191564 -0.227212

5 6 0 3.950269 -0.722334 0.244807

6 6 0 4.218782 -2.164414 -0.239826

7 6 0 4.802024 1.611680 -0.304709

8 6 0 3.555838 2.118829 -0.161508

9 6 0 2.381109 1.274991 -0.044041

10 6 0 2.613644 -0.193970 -0.377755

11 6 0 1.162939 1.777696 0.249147

12 6 0 -0.117153 0.951595 0.322001

13 6 0 0.074339 -0.364713 -0.454194

14 6 0 1.386392 -1.068120 -0.083012

15 6 0 0.817383 3.243318 0.438935

16 6 0 -0.717362 3.322241 0.276443

17 6 0 -1.125463 1.956285 -0.339011

18 6 0 -0.440642 0.687738 1.816677

19 6 0 3.906797 -0.669994 1.792254

20 8 0 7.841665 -2.068288 -0.681291

21 6 0 -2.629671 1.714294 -0.356256

22 6 0 -3.417694 2.905997 -0.855409

23 6 0 -3.198638 0.546344 -0.030310

24 6 0 -4.648818 0.185079 -0.059676

25 6 0 -5.021459 -0.954577 -1.032995

26 6 0 -6.223955 -1.599166 -0.327281

27 6 0 -5.925382 -1.340129 1.148305

28 8 0 -5.019993 -0.331104 1.258981

29 8 0 -6.383591 -1.898487 2.105680

30 6 0 -6.499939 -3.067771 -0.633592

31 6 0 -5.262040 -0.485888 -2.464019

32 1 0 -5.289687 1.051012 -0.241444

33 1 0 -7.125373 -1.009941 -0.555058

34 1 0 5.781008 -2.791511 1.142873

35 1 0 5.829770 -3.630616 -0.390633

36 1 0 7.114227 0.400712 -0.820301

37 1 0 3.492056 -2.848723 0.207063

38 1 0 4.057395 -2.203944 -1.324318

39 1 0 5.643490 2.281243 -0.460409

40 1 0 3.426338 3.196737 -0.156781

41 1 0 2.776035 -0.221796 -1.467205

42 1 0 -0.757673 -1.051153 -0.277472

43 1 0 0.071990 -0.147076 -1.529600

44 1 0 1.458223 -2.004694 -0.642676

45 1 0 1.359179 -1.349373 0.973132

46 1 0 1.322714 3.866447 -0.304853

47 1 0 1.139377 3.617525 1.416614

48 1 0 -1.211152 3.465721 1.241494

49 1 0 -1.012505 4.162142 -0.354173

50 1 0 -0.827660 2.004166 -1.399693

51 1 0 0.362381 0.117112 2.286319

52 1 0 -0.537708 1.617341 2.381353

53 1 0 -1.369573 0.132315 1.949359

54 1 0 4.870411 -0.948765 2.222584

55 1 0 3.668501 0.332855 2.151798

56 1 0 3.157226 -1.358277 2.190530

57 1 0 -2.996510 3.280894 -1.794887

58 1 0 -4.467973 2.685318 -1.037088

59 1 0 -3.374005 3.734745 -0.142560

60 1 0 -2.576821 -0.276986 0.300210

61 1 0 -4.196350 -1.679589 -1.027132

62 1 0 -7.315184 -3.443194 -0.012611

63 1 0 -6.778601 -3.204389 -1.681571

64 1 0 -5.619387 -3.684576 -0.432931

65 1 0 -5.505019 -1.325823 -3.119941

66 1 0 -4.374869 0.004115 -2.874583

67 1 0 -6.092836 0.225593 -2.515122

---------------------------------------------------------------------

**C4**


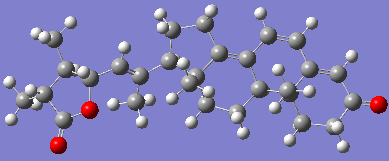


E= -1314.3647582 a.u.

Standard orientation:

---------------------------------------------------------------------

Center Atomic Atomic Coordinates (Angstroms)

Number Number Type X Y Z

---------------------------------------------------------------------

1 6 0 6.422548 -2.015504 0.186771

2 6 0 7.271956 -0.867976 -0.335593

3 6 0 6.595422 0.417807 -0.518386

4 6 0 5.275996 0.626285 -0.274583

5 6 0 4.357005 -0.481000 0.249062

6 6 0 4.936277 -1.856983 -0.147946

7 6 0 4.697780 1.944685 -0.448022

8 6 0 3.369264 2.180655 -0.345730

9 6 0 2.402636 1.113629 -0.169310

10 6 0 2.949327 -0.289201 -0.408635

11 6 0 1.100217 1.361748 0.086515

12 6 0 0.036621 0.280690 0.216985

13 6 0 0.507102 -0.997500 -0.497044

14 6 0 1.930544 -1.390451 -0.077834

15 6 0 0.441805 2.730921 0.130035

16 6 0 -1.076370 2.450568 0.077575

17 6 0 -1.195771 1.006375 -0.451803

18 6 0 -0.259339 -0.011875 1.707260

19 6 0 4.278706 -0.349117 1.790282

20 8 0 8.464278 -1.006905 -0.568663

21 6 0 -2.549706 0.321856 -0.340408

22 6 0 -2.747815 -0.874111 -1.248763

23 6 0 -3.489439 0.758402 0.510710

24 6 0 -4.862158 0.201830 0.769020

25 6 0 -5.981488 0.625165 -0.211399

26 6 0 -6.982357 -0.531013 -0.060515

27 6 0 -6.081859 -1.713265 0.292869

28 8 0 -4.885131 -1.254753 0.748103

29 8 0 -6.327747 -2.884248 0.207780

30 6 0 -7.900992 -0.810267 -1.245917

31 6 0 -6.536941 2.020369 0.055433

32 1 0 -5.158789 0.493616 1.784730

33 1 0 -7.596553 -0.350619 0.834908

34 1 0 6.576309 -2.064449 1.272094

35 1 0 6.825701 -2.948215 -0.215433

36 1 0 7.224516 1.232686 -0.867691

37 1 0 4.365745 -2.654570 0.336235

38 1 0 4.803259 -1.991488 -1.228532

39 1 0 5.379373 2.767497 -0.645780

40 1 0 3.010308 3.202971 -0.416627

41 1 0 3.134954 -0.340988 -1.493719

42 1 0 -0.175205 -1.826709 -0.290065

43 1 0 0.485080 -0.834567 -1.581542

44 1 0 2.210451 -2.315858 -0.588309

45 1 0 1.943512 -1.619763 0.990783

46 1 0 0.757426 3.334660 -0.727210

47 1 0 0.719445 3.302105 1.022118

48 1 0 -1.503222 2.542991 1.077737

49 1 0 -1.612631 3.158382 -0.558784

50 1 0 -0.957961 1.045076 -1.524622

51 1 0 0.619383 -0.417316 2.211086

52 1 0 -0.551231 0.887442 2.253992

53 1 0 -1.074229 -0.734002 1.805567

54 1 0 5.271792 -0.398010 2.240892

55 1 0 3.832932 0.602081 2.087669

56 1 0 3.680049 -1.153072 2.225916

57 1 0 -2.472049 -1.808130 -0.751411

58 1 0 -3.782208 -0.991685 -1.568530

59 1 0 -2.129708 -0.787304 -2.145343

60 1 0 -3.270796 1.626330 1.124006

61 1 0 -5.567200 0.595336 -1.226669

62 1 0 -8.502314 -1.702861 -1.064525

63 1 0 -8.579798 0.027958 -1.422543

64 1 0 -7.325043 -0.978746 -2.160288

65 1 0 -7.327557 2.273301 -0.655750

66 1 0 -5.758737 2.782796 -0.038604

67 1 0 -6.960028 2.094056 1.062711

---------------------------------------------------------------------

**C5**


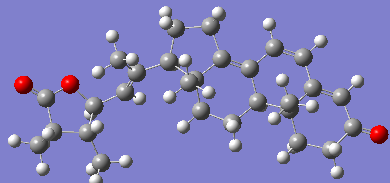


E= -1314.364495 a.u.

Standard orientation:

---------------------------------------------------------------------

Center Atomic Atomic Coordinates (Angstroms)

Number Number Type X Y Z

---------------------------------------------------------------------

1 6 0 5.961147 -2.336437 0.312337

2 6 0 6.929331 -1.353945 -0.327042

3 6 0 6.415829 -0.010247 -0.600725

4 6 0 5.144770 0.388589 -0.337015

5 6 0 4.115397 -0.543129 0.309152

6 6 0 4.495231 -2.010164 0.011438

7 6 0 4.731577 1.752216 -0.606868

8 6 0 3.449492 2.166999 -0.481949

9 6 0 2.362959 1.253846 -0.181937

10 6 0 2.716058 -0.223662 -0.317049

11 6 0 1.114489 1.688408 0.094067

12 6 0 -0.064323 0.762275 0.350285

13 6 0 0.205158 -0.618833 -0.264041

14 6 0 1.583688 -1.159605 0.137625

15 6 0 0.623708 3.126520 0.049789

16 6 0 -0.920772 3.027793 0.003161

17 6 0 -1.212680 1.557902 -0.373680

18 6 0 -0.314633 0.633367 1.871384

19 6 0 4.121957 -0.278411 1.835086

20 8 0 8.084748 -1.665015 -0.578802

21 6 0 -2.644450 1.076417 -0.174503

22 6 0 -3.429320 1.640913 0.985840

23 6 0 -3.155524 0.220461 -1.073825

24 6 0 -4.522557 -0.405337 -1.137511

25 6 0 -4.807465 -1.583350 -0.176820

26 6 0 -6.340365 -1.540455 -0.077828

27 6 0 -6.648967 -0.057541 -0.276574

28 8 0 -5.585657 0.555037 -0.862983

29 8 0 -7.648386 0.537305 0.017017

30 6 0 -6.971282 -2.118680 1.185068

31 6 0 -4.212714 -2.910571 -0.636099

32 1 0 -4.686622 -0.748290 -2.166742

33 1 0 -6.764168 -2.050866 -0.956167

34 1 0 6.150952 -2.322505 1.392790

35 1 0 6.224415 -3.341924 -0.025171

36 1 0 7.129595 0.683690 -1.037522

37 1 0 3.847695 -2.685283 0.578230

38 1 0 4.301139 -2.210302 -1.049468

39 1 0 5.502714 2.460046 -0.898251

40 1 0 3.220308 3.217982 -0.629918

41 1 0 2.847389 -0.385331 -1.399220

42 1 0 -0.578101 -1.325187 0.030198

43 1 0 0.159093 -0.539559 -1.357153

44 1 0 1.725234 -2.148155 -0.307683

45 1 0 1.617431 -1.310068 1.219921

46 1 0 1.011043 3.640836 -0.835158

47 1 0 0.966316 3.708141 0.912264

48 1 0 -1.349808 3.273916 0.976277

49 1 0 -1.360488 3.723220 -0.715732

50 1 0 -0.989381 1.455659 -1.442204

51 1 0 0.547680 0.190555 2.372155

52 1 0 -0.489549 1.601576 2.345346

53 1 0 -1.180389 -0.004324 2.070854

54 1 0 5.120921 -0.415559 2.253292

55 1 0 3.809519 0.742302 2.063711

56 1 0 3.449273 -0.962524 2.358654

57 1 0 -3.721483 2.675847 0.778076

58 1 0 -4.347108 1.093173 1.182193

59 1 0 -2.840533 1.659483 1.904792

60 1 0 -2.512743 -0.079423 -1.898713

61 1 0 -4.389964 -1.324869 0.804106

62 1 0 -8.049483 -1.949349 1.187508

63 1 0 -6.795411 -3.195075 1.254982

64 1 0 -6.560260 -1.648494 2.082930

65 1 0 -4.447205 -3.712712 0.068467

66 1 0 -3.123493 -2.853265 -0.714599

67 1 0 -4.604601 -3.204011 -1.615508

---------------------------------------------------------------------

Lowest energy conformers of compound **(23S, 25S, 26S)-6** for NMR calculation

**C1**


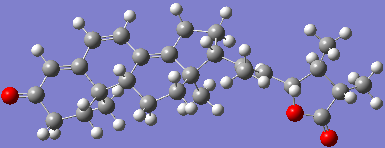


E= -1314.3694912 a.u.

Standard orientation:

---------------------------------------------------------------------

Center Atomic Atomic Coordinates (Angstroms)

Number Number Type X Y Z

---------------------------------------------------------------------

1 6 0 -6.455527 -1.929308 0.330870

2 6 0 -7.312868 -0.700600 0.073012

3 6 0 -6.604772 0.527583 -0.294128

4 6 0 -5.254257 0.628163 -0.393790

5 6 0 -4.326980 -0.565486 -0.147919

6 6 0 -5.033403 -1.579184 0.779010

7 6 0 -4.639046 1.875329 -0.804915

8 6 0 -3.299459 2.066488 -0.800754

9 6 0 -2.376091 1.081400 -0.270234

10 6 0 -3.028035 -0.030570 0.544538

11 6 0 -1.038703 1.203508 -0.408428

12 6 0 -0.032702 0.227926 0.182795

13 6 0 -0.668299 -0.538421 1.353527

14 6 0 -2.035958 -1.123347 0.974807

15 6 0 -0.298048 2.375240 -1.032689

16 6 0 1.172821 2.211912 -0.582471

17 6 0 1.114763 1.224976 0.603753

18 6 0 0.453710 -0.755860 -0.906429

19 6 0 -4.005509 -1.213181 -1.517299

20 8 0 -8.532706 -0.742745 0.146427

21 6 0 2.428791 0.620740 1.077801

22 6 0 2.516761 0.427664 2.573313

23 6 0 3.414355 0.310794 0.223926

24 6 0 4.749791 -0.283950 0.530226

25 6 0 5.961877 0.541513 0.046483

26 6 0 7.002908 -0.550858 -0.240355

27 6 0 6.137454 -1.746677 -0.633547

28 8 0 4.866534 -1.554576 -0.188800

29 8 0 6.464621 -2.731972 -1.233925

30 6 0 8.078798 -0.227732 -1.272533

31 6 0 6.392769 1.629959 1.024020

32 1 0 4.864451 -0.517877 1.591492

33 1 0 7.487855 -0.835269 0.705862

34 1 0 -6.439607 -2.514047 -0.597401

35 1 0 -6.966067 -2.552227 1.069419

36 1 0 -7.236147 1.388624 -0.498658

37 1 0 -4.438396 -2.493904 0.853246

38 1 0 -5.077740 -1.154087 1.789379

39 1 0 -5.297385 2.667676 -1.150457

40 1 0 -2.900263 2.998105 -1.190591

41 1 0 -3.380349 0.460956 1.465757

42 1 0 -0.006558 -1.344293 1.688472

43 1 0 -0.794599 0.143205 2.204023

44 1 0 -2.440509 -1.668989 1.831402

45 1 0 -1.905548 -1.863297 0.181008

46 1 0 -0.712906 3.324975 -0.680079

47 1 0 -0.388995 2.391106 -2.123846

48 1 0 1.777579 1.813752 -1.399027

49 1 0 1.629570 3.160614 -0.290818

50 1 0 0.703079 1.785495 1.454382

51 1 0 -0.368774 -1.379224 -1.260694

52 1 0 0.853794 -0.237963 -1.780923

53 1 0 1.238867 -1.410783 -0.521584

54 1 0 -4.919256 -1.501133 -2.040268

55 1 0 -3.461706 -0.524835 -2.167074

56 1 0 -3.398024 -2.113199 -1.395290

57 1 0 1.648785 -0.122211 2.947330

58 1 0 3.409560 -0.105833 2.897473

59 1 0 2.507675 1.398228 3.083196

60 1 0 3.267888 0.489736 -0.838008

61 1 0 5.686870 1.006986 -0.909613

62 1 0 8.700019 -1.104021 -1.465927

63 1 0 8.727989 0.580242 -0.925504

64 1 0 7.633326 0.078855 -2.223127

65 1 0 7.244415 2.194457 0.635816

66 1 0 5.583521 2.342192 1.207184

67 1 0 6.689143 1.204188 1.988289

---------------------------------------------------------------------

**C2**


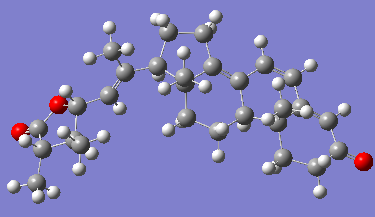


E= -1314.3687494 a.u.

Standard orientation:

---------------------------------------------------------------------

Center Atomic Atomic Coordinates (Angstroms)

Number Number Type X Y Z

---------------------------------------------------------------------

1 6 0 5.695427 -2.518540 0.516646

2 6 0 6.640695 -1.815974 -0.444970

3 6 0 6.194823 -0.527521 -0.979343

4 6 0 5.002317 0.049602 -0.681899

5 6 0 4.001838 -0.595158 0.281574

6 6 0 4.230684 -2.122679 0.308317

7 6 0 4.658526 1.346958 -1.230659

8 6 0 3.438848 1.910540 -1.069971

9 6 0 2.338981 1.211785 -0.432322

10 6 0 2.559266 -0.285561 -0.243289

11 6 0 1.177348 1.828601 -0.127302

12 6 0 -0.015325 1.120058 0.494821

13 6 0 0.054577 -0.384892 0.198112

14 6 0 1.430109 -0.968823 0.547144

15 6 0 0.793118 3.257872 -0.473916

16 6 0 -0.740079 3.326386 -0.274450

17 6 0 -1.200500 1.851673 -0.236994

18 6 0 -0.039348 1.366294 2.022058

19 6 0 4.230464 0.012827 1.687367

20 8 0 7.727434 -2.293223 -0.738422

21 6 0 -2.613292 1.588566 0.268453

22 6 0 -3.170728 2.503776 1.332834

23 6 0 -3.310373 0.585156 -0.286973

24 6 0 -4.691508 0.117286 0.035472

25 6 0 -4.797787 -1.354653 0.491556

26 6 0 -6.197534 -1.744809 -0.005330

27 6 0 -6.374070 -0.862361 -1.239373

28 8 0 -5.497909 0.176914 -1.184634

29 8 0 -7.146462 -1.003421 -2.145779

30 6 0 -6.444257 -3.224085 -0.284429

31 6 0 -4.542589 -1.555491 1.981656

32 1 0 -5.186200 0.763937 0.764604

33 1 0 -6.942733 -1.388369 0.722133

34 1 0 6.026371 -2.273182 1.533577

35 1 0 5.837765 -3.596443 0.406965

36 1 0 6.893024 -0.031261 -1.648630

37 1 0 3.614613 -2.577998 1.089003

38 1 0 3.885305 -2.542199 -0.644653

39 1 0 5.434368 1.882553 -1.770869

40 1 0 3.270162 2.915434 -1.445244

41 1 0 2.533511 -0.705036 -1.261897

42 1 0 -0.730251 -0.918265 0.744546

43 1 0 -0.142663 -0.547672 -0.868414

44 1 0 1.429648 -2.040685 0.331348

45 1 0 1.602795 -0.879522 1.622814

46 1 0 1.064120 3.488637 -1.509002

47 1 0 1.315373 3.993240 0.147200

48 1 0 -0.977789 3.836419 0.661269

49 1 0 -1.238290 3.885649 -1.070034

50 1 0 -1.162455 1.481482 -1.268545

51 1 0 0.857970 0.966510 2.496778

52 1 0 -0.077057 2.429388 2.270499

53 1 0 -0.904434 0.879240 2.481184

54 1 0 5.260672 -0.135102 2.016697

55 1 0 4.037493 1.087403 1.694122

56 1 0 3.577459 -0.452374 2.430300

57 1 0 -3.293078 3.517460 0.937350

58 1 0 -4.143487 2.182215 1.702272

59 1 0 -2.504973 2.585896 2.194824

60 1 0 -2.844445 0.015907 -1.090175

61 1 0 -4.060795 -1.934470 -0.080373

62 1 0 -7.436939 -3.374000 -0.712518

63 1 0 -6.376146 -3.812981 0.633870

64 1 0 -5.713568 -3.618474 -0.996133

65 1 0 -4.605631 -2.611829 2.255370

66 1 0 -3.546916 -1.202654 2.264172

67 1 0 -5.275087 -1.012019 2.587547

---------------------------------------------------------------------

**C3**


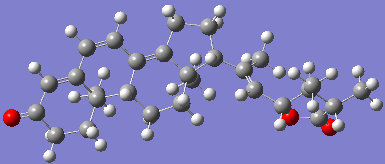


E= -1314.3651386 a.u.

Standard orientation:

---------------------------------------------------------------------

Center Atomic Atomic Coordinates (Angstroms)

Number Number Type X Y Z

---------------------------------------------------------------------

1 6 0 -6.160157 -2.251067 -0.084117

2 6 0 -7.063419 -1.194923 0.532300

3 6 0 -6.483398 0.136721 0.718554

4 6 0 -5.205449 0.463478 0.396119

5 6 0 -4.238756 -0.545900 -0.229493

6 6 0 -4.672418 -1.976926 0.156307

7 6 0 -4.725190 1.819038 0.581762

8 6 0 -3.430723 2.168923 0.398923

9 6 0 -2.395573 1.193583 0.113590

10 6 0 -2.807641 -0.257565 0.337790

11 6 0 -1.139839 1.556434 -0.225470

12 6 0 -0.016336 0.565304 -0.482315

13 6 0 -0.316836 -0.766136 0.221215

14 6 0 -1.731762 -1.266690 -0.097889

15 6 0 -0.585444 2.971119 -0.266837

16 6 0 0.953463 2.804295 -0.298942

17 6 0 1.203615 1.343675 0.137159

18 6 0 0.159704 0.339727 -2.002939

19 6 0 -4.280271 -0.360824 -1.766675

20 8 0 -8.222260 -1.440330 0.835962

21 6 0 2.592257 0.774595 -0.126250

22 6 0 3.351138 1.274832 -1.332072

23 6 0 3.077085 -0.128641 0.740977

24 6 0 4.381786 -0.877449 0.732281

25 6 0 5.641442 -0.100976 1.179646

26 6 0 6.760118 -0.913539 0.508815

27 6 0 6.076033 -1.451835 -0.745868

28 8 0 4.725427 -1.396261 -0.587728

29 8 0 6.585133 -1.869560 -1.748333

30 6 0 8.066515 -0.183756 0.212830

31 6 0 5.759407 0.065494 2.690909

32 1 0 4.267575 -1.756681 1.378561

33 1 0 6.975265 -1.796501 1.130168

34 1 0 -6.382641 -2.281362 -1.158005

35 1 0 -6.456682 -3.225689 0.311301

36 1 0 -7.151771 0.883722 1.139469

37 1 0 -4.073294 -2.708754 -0.392883

38 1 0 -4.453201 -2.130617 1.220050

39 1 0 -5.455540 2.574972 0.857108

40 1 0 -3.151854 3.214957 0.484503

41 1 0 -2.912031 -0.355844 1.430564

42 1 0 0.422921 -1.522186 -0.060245

43 1 0 -0.224579 -0.626768 1.305643

44 1 0 -1.897017 -2.220487 0.410490

45 1 0 -1.811092 -1.479276 -1.167088

46 1 0 -0.900548 3.534230 0.617417

47 1 0 -0.949478 3.536023 -1.131559

48 1 0 1.334332 2.981192 -1.306386

49 1 0 1.461546 3.516432 0.356288

50 1 0 1.041519 1.301388 1.221353

51 1 0 -0.738846 -0.099340 -2.439221

52 1 0 0.345501 1.272143 -2.540753

53 1 0 0.993655 -0.339355 -2.199775

54 1 0 -5.297107 -0.469875 -2.148568

55 1 0 -3.925875 0.629475 -2.059091

56 1 0 -3.657676 -1.104377 -2.270660

57 1 0 3.668539 2.312363 -1.175500

58 1 0 4.232699 0.677493 -1.546479

59 1 0 2.733595 1.276496 -2.232640

60 1 0 2.455188 -0.376321 1.598905

61 1 0 5.605998 0.890390 0.711165

62 1 0 8.750040 -0.829011 -0.341876

63 1 0 8.562631 0.123268 1.136967

64 1 0 7.890959 0.710421 -0.391835

65 1 0 6.669621 0.608532 2.958225

66 1 0 4.913620 0.627382 3.096520

67 1 0 5.791522 -0.905162 3.196796

---------------------------------------------------------------------

C4


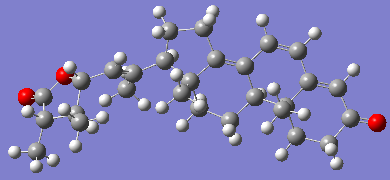


E= -1314.3644074 a.u.

Standard orientation:

---------------------------------------------------------------------

Center Atomic Atomic Coordinates (Angstroms)

Number Number Type X Y Z

---------------------------------------------------------------------

1 6 0 6.129716 -2.277618 -0.101607

2 6 0 7.089898 -1.132504 -0.382114

3 6 0 6.525080 0.218310 -0.353791

4 6 0 5.216399 0.492113 -0.117239

5 6 0 4.191347 -0.607948 0.171119

6 6 0 4.677582 -1.936329 -0.449310

7 6 0 4.750881 1.864635 -0.067967

8 6 0 3.441626 2.190444 0.036974

9 6 0 2.387367 1.194830 -0.010790

10 6 0 2.833317 -0.183713 -0.483730

11 6 0 1.098063 1.502155 0.246632

12 6 0 -0.048533 0.507026 0.146007

13 6 0 0.349611 -0.664365 -0.765697

14 6 0 1.721571 -1.240376 -0.387919

15 6 0 0.539615 2.888668 0.525065

16 6 0 -0.990925 2.752145 0.349807

17 6 0 -1.181167 1.438115 -0.437043

18 6 0 -0.428554 -0.010989 1.552427

19 6 0 4.054184 -0.742913 1.707873

20 8 0 8.277328 -1.329608 -0.596898

21 6 0 -2.590110 0.866963 -0.530843

22 6 0 -2.888959 0.183591 -1.844001

23 6 0 -3.480457 1.012769 0.460898

24 6 0 -4.907554 0.539429 0.541325

25 6 0 -5.136778 -0.956789 0.857915

26 6 0 -6.541196 -1.186752 0.278068

27 6 0 -6.597204 -0.185615 -0.874265

28 8 0 -5.642556 0.766904 -0.697475

29 8 0 -7.337663 -0.182112 -1.817904

30 6 0 -6.891452 -2.609348 -0.147096

31 6 0 -4.966896 -1.310213 2.331790

32 1 0 -5.416649 1.139738 1.305783

33 1 0 -7.286216 -0.837331 1.009184

34 1 0 6.227469 -2.529348 0.961810

35 1 0 6.477050 -3.155793 -0.651398

36 1 0 7.231141 1.025412 -0.532123

37 1 0 4.024974 -2.756099 -0.135235

38 1 0 4.584333 -1.865245 -1.540043

39 1 0 5.502443 2.648896 -0.094378

40 1 0 3.166814 3.235736 0.140805

41 1 0 3.062709 -0.057010 -1.554034

42 1 0 -0.403784 -1.458227 -0.722581

43 1 0 0.382134 -0.317202 -1.805552

44 1 0 1.952289 -2.077442 -1.052404

45 1 0 1.674866 -1.660574 0.620287

46 1 0 0.950134 3.616649 -0.181972

47 1 0 0.804501 3.252904 1.523297

48 1 0 -1.480262 2.706122 1.324215

49 1 0 -1.431427 3.600422 -0.178563

50 1 0 -0.867532 1.648388 -1.469012

51 1 0 0.388258 -0.584654 1.992954

52 1 0 -0.652838 0.802264 2.246296

53 1 0 -1.309955 -0.655144 1.499434

54 1 0 5.019302 -0.953812 2.172259

55 1 0 3.670734 0.175593 2.156519

56 1 0 3.375767 -1.557405 1.973869

57 1 0 -2.098889 -0.527083 -2.101185

58 1 0 -3.841382 -0.338462 -1.860290

59 1 0 -2.915170 0.923545 -2.652358

60 1 0 -3.169829 1.546981 1.353445

61 1 0 -4.418841 -1.536721 0.264801

62 1 0 -7.871403 -2.636562 -0.626893

63 1 0 -6.915315 -3.282404 0.713726

64 1 0 -6.161543 -2.998887 -0.862397

65 1 0 -5.153809 -2.373021 2.505910

66 1 0 -3.952675 -1.096110 2.679980

67 1 0 -5.663091 -0.743914 2.959139

---------------------------------------------------------------------

**C5**


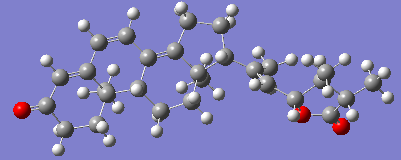


E= -1314.3643836 a.u.

Standard orientation:

---------------------------------------------------------------------

Center Atomic Atomic Coordinates (Angstroms)

Number Number Type X Y Z

---------------------------------------------------------------------

1 6 0 -6.252205 -2.163522 0.047520

2 6 0 -7.101581 -1.066991 0.670088

3 6 0 -6.477777 0.251679 0.798466

4 6 0 -5.203241 0.531470 0.422991

5 6 0 -4.286638 -0.522483 -0.203892

6 6 0 -4.749731 -1.929468 0.232643

7 6 0 -4.678948 1.877257 0.552047

8 6 0 -3.381518 2.183255 0.319176

9 6 0 -2.383037 1.169991 0.034899

10 6 0 -2.830266 -0.262278 0.311652

11 6 0 -1.127129 1.484573 -0.348353

12 6 0 -0.037301 0.454194 -0.596127

13 6 0 -0.361424 -0.852808 0.140891

14 6 0 -1.798968 -1.314084 -0.131112

15 6 0 -0.532608 2.879578 -0.456698

16 6 0 1.002760 2.668589 -0.474949

17 6 0 1.203992 1.211560 0.001762

18 6 0 0.106525 0.196017 -2.115033

19 6 0 -4.369902 -0.374955 -1.743363

20 8 0 -8.254248 -1.271071 1.023678

21 6 0 2.579119 0.596729 -0.205253

22 6 0 3.261313 0.720847 -1.554034

23 6 0 3.136987 -0.050351 0.830994

24 6 0 4.456624 -0.765345 0.905424

25 6 0 5.702853 0.109399 1.184047

26 6 0 6.827208 -0.740802 0.572937

27 6 0 6.112697 -1.489050 -0.550605

28 8 0 4.771525 -1.473740 -0.326999

29 8 0 6.595141 -2.030118 -1.506251

30 6 0 8.074876 -0.000569 0.100996

31 6 0 5.878733 0.480779 2.652818

32 1 0 4.384315 -1.533333 1.686236

33 1 0 7.121762 -1.513198 1.299735

34 1 0 -6.510979 -2.208581 -1.017731

35 1 0 -6.564268 -3.120121 0.473667

36 1 0 -7.109248 1.029520 1.220440

37 1 0 -4.190908 -2.692334 -0.316682

38 1 0 -4.500165 -2.063687 1.292368

39 1 0 -5.378304 2.662301 0.826410

40 1 0 -3.070844 3.222788 0.364553

41 1 0 -2.902377 -0.329825 1.409313

42 1 0 0.346706 -1.637613 -0.143842

43 1 0 -0.233545 -0.696417 1.219253

44 1 0 -1.980767 -2.251933 0.400607

45 1 0 -1.913260 -1.544138 -1.193770

46 1 0 -0.833522 3.497574 0.394809

47 1 0 -0.878876 3.407673 -1.351562

48 1 0 1.397054 2.818954 -1.481890

49 1 0 1.526583 3.376094 0.172396

50 1 0 1.027214 1.214869 1.084307

51 1 0 -0.819600 -0.206517 -2.528783

52 1 0 0.328508 1.108662 -2.672444

53 1 0 0.903167 -0.523840 -2.316853

54 1 0 -5.400509 -0.463529 -2.092139

55 1 0 -3.996968 0.597312 -2.071051

56 1 0 -3.784046 -1.148409 -2.246272

57 1 0 2.745436 1.415346 -2.214695

58 1 0 4.288390 1.079386 -1.451144

59 1 0 3.321890 -0.242112 -2.063974

60 1 0 2.581619 -0.083027 1.765901

61 1 0 5.606157 1.028879 0.593558

62 1 0 8.763367 -0.685849 -0.396615

63 1 0 8.600030 0.459503 0.941918

64 1 0 7.819422 0.788213 -0.612218

65 1 0 6.772708 1.092271 2.800239

66 1 0 5.026162 1.056616 3.023260

67 1 0 5.979871 -0.411317 3.279671

---------------------------------------------------------------------


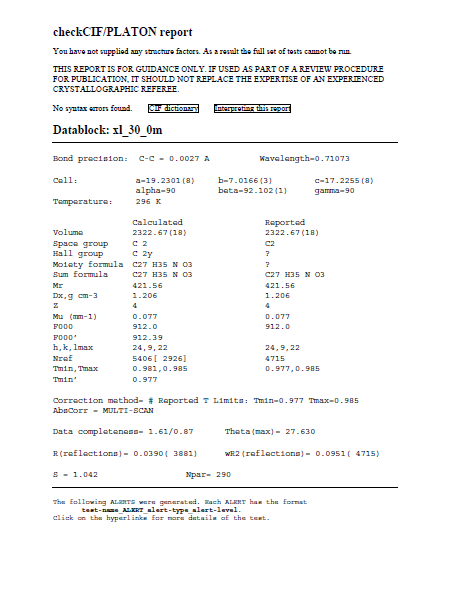

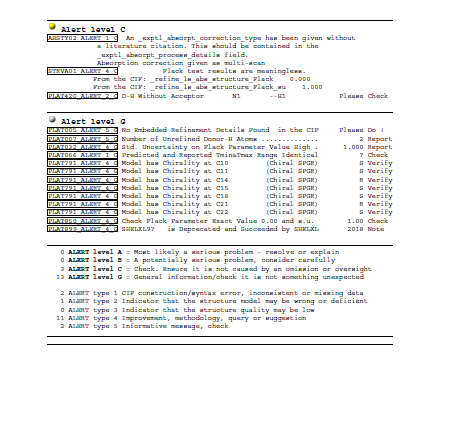

Supplement: Supplementary file 1 [file Data_Sheet_1.DOCX]
